# Diverse Biological Activity of Benzofuroxan/Sterically Hindered Phenols Hybrids

**DOI:** 10.3390/ph16040499

**Published:** 2023-03-28

**Authors:** Elena Chugunova, Elmira Gibadullina, Kirill Matylitsky, Baurat Bazarbayev, Margarita Neganova, Konstantin Volcho, Artem Rogachev, Nurgali Akylbekov, Hoang Bao Tran Nguyen, Alexandra Voloshina, Anna Lyubina, Syumbelya Amerhanova, Victor Syakaev, Alexander Burilov, Nurbol Appazov, Mukhtar Zhanakov, Leah Kuhn, Oleg Sinyashin, Igor Alabugin

**Affiliations:** 1 Arbuzov Institute of Organic and Physical Chemistry, FRC Kazan Scientific Center, Russian Academy of Sciences, Akad. Arbuzov St. 8, 420088 Kazan, Russia; 2 The Department of General Organic and Petrochemical Synthesis Technology, The Kazan National Research Technological University, Karl Marx St., 68, 420015 Kazan, Russia; 3 Department of Medicinal Chemistry, Novosibirsk Institute of Organic Chemistry, Lavrentiev Av. 9, 630090 Novosibirsk, Russia; 4 Zelman Institute for Medicine and Psychology, Novosibirsk State University, Pirogov St. 2, 630090 Novosibirsk, Russia; 5 Laboratory of Engineering Profile “Physical and Chemical Methods of Analysis”, Korkyt Ata Kyzylorda University, Aitekebie Str. 29A, Kyzylorda 120014, Kazakhstan; 6 I. Zhakhaev Kazakh Scientific Research Institute of Rice Growing, Abay Av. 25B, Kyzylorda 120008, Kazakhstan; 7 Faculty of Natural Sciences, L.N. Gumilyov Eurasian National University, Satpayev Str. 2, Astana 010000, Kazakhstan; 8 Department of Chemistry and Biochemistry, Florida State University, 95 Chieftan Way, Tallahassee, FL 32306-4390, USA

**Keywords:** benzofuroxan, sterically hindered phenol, anti-cancer activity, cytotoxicity, apoptosis, ROS production

## Abstract

Combining two pharmacophores in a molecule can lead to useful synergistic effects. Herein, we show hybrid systems that combine sterically hindered phenols with dinitrobenzofuroxan fragments exhibit a broad range of biological activities. The modular assembly of such phenol/benzofuroxan hybrids allows variations in the phenol/benzofuroxan ratio. Interestingly, the antimicrobial activity only appears when at least two benzofuroxan moieties are introduced per phenol. The most potent of the synthesized compounds exhibit high cytotoxicity against human duodenal adenocarcinoma (HuTu 80), human breast adenocarcinoma (MCF-7), and human cervical carcinoma cell lines. This toxicity is associated with the induction of apoptosis via the internal mitochondrial pathway and an increase in ROS production. Encouragingly, the index of selectivity relative to healthy tissues exceeds that for the reference drugs Doxorubicin and Sorafenib. The biostability of the leading compounds in whole mice blood is sufficiently high for their future quantification in biological matrices.

## 1. Introduction

Cancer continues to be one of the most serious challenges facing modern science and medicine. Oncological diseases steadily occupy the second place in the list of causes of human death. According to the World Health Organization, by 2030, 15 million people will be dying from this pathology in the world every year [1].

In our search for new highly effective antitumor drugs, we chose a molecular design that can achieve two main goals: (1) to reduce toxicity in a healthy microenvironment and to provide a targeted effect on tumor tissues; (2) to enhance the antitumor effect by combining in one molecule several pharmacophores with different but complementary mechanisms of action.

**Figure 1:**
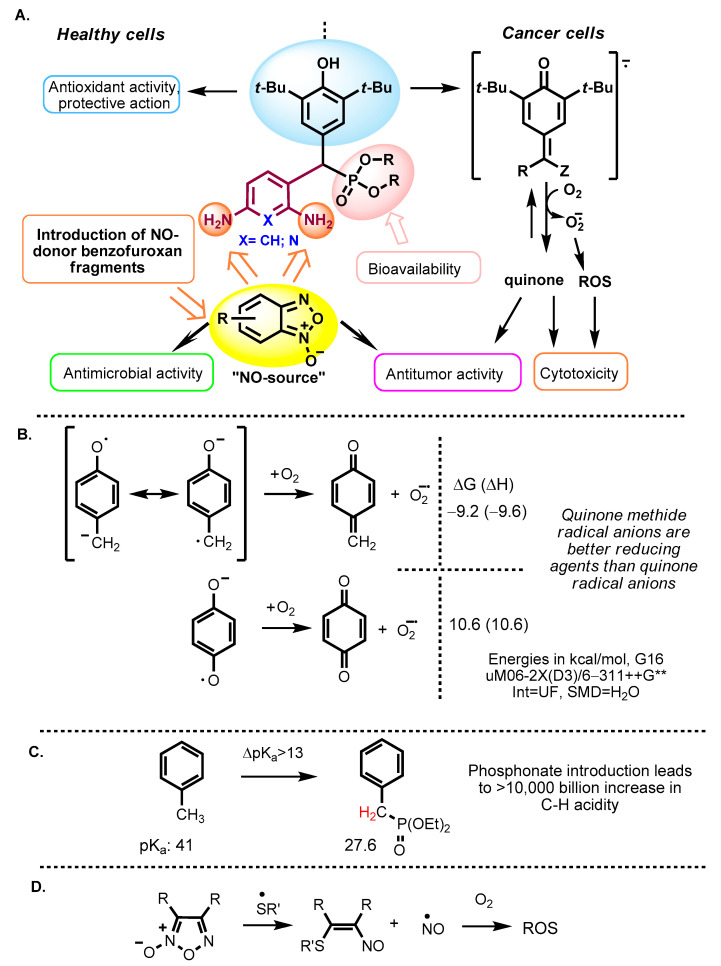
(**A**) General approach to the creation of new types of hybrid antitumor agents based on sterically hindered phenols (SHP) and benzofuroxans. (**B**) Redox cycling between phenols and quinone methides creates radical anions that can reduce molecular oxygen to a superoxide anion. (**C**) Phosphonate group greatly activates benzylic C-H bonds and facilitates quinone methide radical-anion formation. (**D**) Generation of NO and ROS from furoxans. ROS = Reactive Oxygen Species.

The inclusion of a sterically hindered phenol (SHP) fragment helps to address the first goal. In healthy cells, where there is a redox balance, phenols will behave as antioxidants, protecting a normal cell from the harmful effects of other pharmacophoric fragments of the structure. The situation changes in a state of oxidative stress observed in tumor cells when numerous ROS (Reactive Oxygen Species) are formed and various metals, mainly iron and copper, accumulate in the unbound state [2,3,4,5,6]. Under these conditions, the phenols are transformed into highly reactive methylene quinones, which have destructive effects on lipids, proteins and DNA, thereby leading to the tumor cells death (Figure 1). The effect of quinones can be multi-fold. Non-hindered quinones are potent electrophiles that can bind to thiol, amine and hydroxyl groups. More generally, the fast redox cycling between phenols and quinones can lead to the formation of intermediates that can transfer an electron to molecular oxygen, transforming it into a superoxide radical anion and, thus, serving themselves as a potentially catalytic source of ROS.

Such phenol-mediated redox reorientation was shown to be promising for the development of targeted antitumor agents, initiating the mitochondrial pathway of cancer cell apoptosis. The spectrum of biological antitumor activity includes inhibiting metastasis of melanoma and Lewis lung carcinoma [7,8,9], leukemia, colon, liver, ovarian, breast cancers [10], sarcoma 37 and carcinoma [11].

The presence of a benzylic phosphonate group is likely to be important based on the earlier indications [12,13,14] that the introduction of phosphate ester or bisphosphonate ester moieties into anticancer agents (e.g., Cisplatin, Camptothecin and Doxorubicin) can improve drug solubility and antitumor activity. An intriguing possibility is that it can also assist in the formation of quinone methides and their radical anions by activating the C-H bond (Figure 1). It is well known that the phosphonate group can increase C-H acidity by >13 orders of magnitude [15].

Considering the redox activity and ROS generation with SHPs, a logical choice for the second pharmacophore is provided by the benzofuroxan moiety. Benzofuroxans represent an important class of lipophilic thiol-dependent NO donors [16,17]. After the discovery of the key role of nitric oxide as an intracellular regulator of metabolism, benzofuroxans have been playing an increasingly important role in the development of anticancer agents that can generate high levels of NO and inhibit tumor growth in vivo [18,19]. Interestingly, these heterocycles can produce not only nitric oxide (NO•) but also its two redox forms, nitroxyl (HNO) and nitrosonium ion (NO+). From this point of view, the synergy between SHP and furoxan moieties in hybrid systems seem to be an attainable goal.

Since the first reports of the antitumor properties of benzofuroxans [20,21,22,23], cytotoxicity against tumor cell lines of many of their derivatives has been tested [24,25,26,27,28]. A number of benzofuroxan derivatives inhibit the growth of M-HeLa cells in vitro. Furthermore, several of them showed significant activity against P388 lymphatic leukemia and Ehrlich ascitic carcinoma in mice [29]. Interestingly, the antitumor effect of these compounds is due to their dual ability to inhibit DNA synthesis and to cause DNA destruction through a series of single- and double-strand breaks [26]. It is well known that single DNA damage can be fairly easily repaired by the numerous intercellular DNA repair systems. In contrast, multiple damages are much harder to repair and, hence, it is much more likely to lead to cell death. It is noteworthy that the same study [26] emphasizes the positive contribution of electron-withdrawing nitro groups in the benzofuroxan core to cytotoxicity.

In addition, NO is a necessary component of a non-specific defense mechanism against many pathogens, including bacteria, viruses and fungi. A number of publications describe the high fungicidal activity of the so-called “hybrid” benzofuroxan derivatives, which include, in addition to the benzofuroxan moiety, known pharmacophore fragments (amino acids, amino alcohol nitrates, phenols and polyene antibiotics) [30,31]. The activity of a 4,6-dinitro-5,7-dichlorobenzofuroxan derivative against *Trichophyton mentagrophytes* was four times higher than the activity of the antifungal drug Nystatin [32]. Benzofuroxan derivatives were found to be highly active against phytopathogenic fungi (*Rhizoctonia solani*, *Sclerotinia sclerotiorum*, *Fusarium graminearum* and *Phytophthora capsici*) [33] and against antibiotic-resistant *Staphylococcus* bacteria [34].

In summary, our aim is to develop agents with a broad spectrum of biological action. Our basic molecular platform is a substituted sterically hindered phenol, which can be reversibly oxidized to the corresponding methylene quinone in cancer cells but exhibits antioxidant activity in healthy cells. The auxiliary components include a diaminopyridine or diaminophenyl linker for targeted modification, a phosphorus-containing fragment for increasing bioavailability, and benzofuroxan as an additional pharmacophore with the potential to be both the NO-donor and apoptosis-inducing agent (Figure 1).

## 2. Results and Discussion

### 2.1. Chemistry

**Scheme 1:**
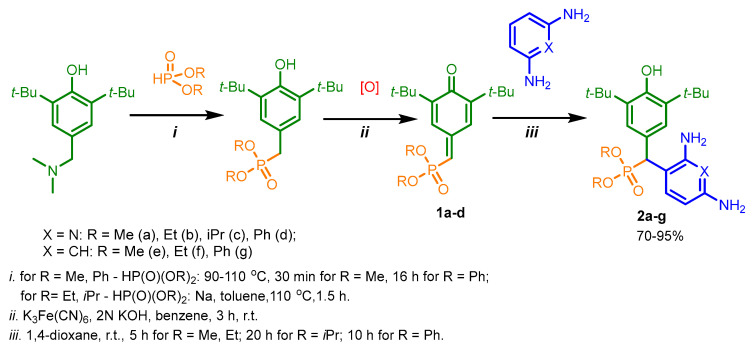
The synthesis of the starting SHPs 2 containing diaminopyridine or diaminophenyl fragments.

Phosphorylated sterically hindered phenols (SHPs) **2** containing diaminopyridine or diaminophenyl fragments were used as a starting point due to their already identified high and selective cytotoxicity [35]. The synthesis of the starting SHPs was carried out in accordance with the original method developed by us earlier (Scheme 1, [35]). The main stages of this method include preparation of the corresponding phosphorylated phenol, its oxidation to methylene quinone **1**, and, as the last step, reaction of the methylene quinone with C-nucleophile, i.e., 2,6-diaminopyridine or 1,3-diaminobenzene. This sequence produces the key compounds **2,** that possess two amino groups suitable for further modifications with heterocyclic fragments.

**Scheme 2:**
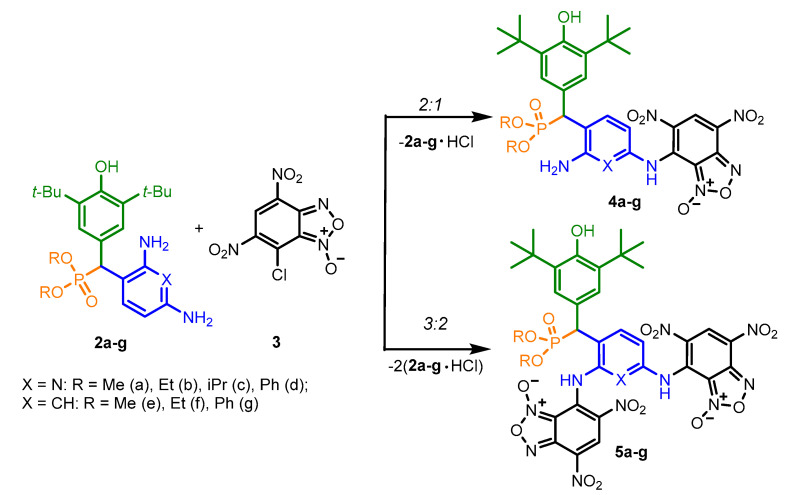
Synthesis of SHP/furoxan hybrids of varying composition.

Finally, the amino groups of the two hindered phosphorylated phenols **2** were used in reactions with nitrochlorobenzofuroxans. This highly reactive heterocycle is superelectrophilic and readily reacts with the appended aniline moieties. The phenol OH group is unreactive under these conditions due to the steric protection provided by the two *tert*-butyl groups. The final products of this synthetic sequence result are previously unknown “hybrid” phosphorus-containing SHPs containing a nitrobenzofuroxan fragment linked to the SHP fragment through a (hetero)aromatic “linker” (Scheme 2).

Depending on the initial ratio of reagents, it is possible to vary the composition of the final products, leading to the formation of compounds with a composition of 2:1 when two molecules of benzofuroxan interact with one molecule of sterically hindered phenol and compounds of the composition of 1:1, when one molecule of benzofuroxan reacts with one molecule of sterically hindered phenol. The excess phosphorus-containing SHP in both cases was used to neutralize the hydrogen chloride formed during the reaction.

**Scheme 3:**
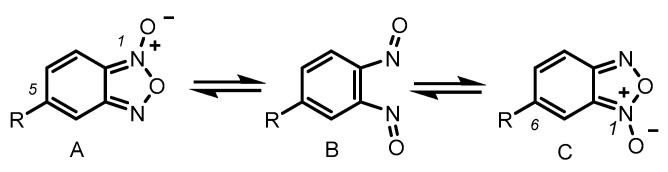
*N*-1-oxide/*N*-3-oxide tautomerism in benzofuroxan derivatives.

As is known in the literature, benzofuroxan rapidly interconverts in solution between two non-symmetric bicyclic structures through the open dinitroso form (so-called *N*-1-oxide/*N*-3-oxide tautomerism, Scheme 3) [36,37]. As a result, the NMR ^1^H and ^13^C spectra of benzofuroxan derivatives taken at room temperatures are often broadened as they correspond to a fast mutual transition of equivalent non-symmetric forms, while the spectra taken at low temperature are non-symmetric forms [38]. In our study, we also observed our products in the spectra, not as individual compounds but as a mixture of tautomers. In some cases, they were separated into the pure form (as in the case of compounds **4e–g**). In other cases, where the content of the second tautomer was insignificant, we limited ourselves to describing only the main structure.

The complete assignment of signals in ^1^H and ^13^C spectra (Figures S1–S45) was accomplished by using the 2D NMR techniques (COSY, ^1^H-^13^C HSQC and ^1^H-^13^C HMBC). Examples of 2D spectra for compounds **4d** and **4e** are presented (Figures S12–S14 and S18–S20). For compounds within the series, the assignment of peaks was conducted by analogy, taking into account the effects of neighboring substituents. Some difficulty is that benzofuroxans exist as two tautomers due to oxygen migration. As we showed earlier [39], the presence of only one proton in the tri-substituted benzofuroxan moiety makes it difficult to use two-dimensional NMR experiments. At the same time, the chemical shifts of carbons in these tautomeric forms do not differ significantly. Nevertheless, the difference between C3a and C7a makes it possible to quite unambiguously attribute the observed shape of the benzofuroxan ring.

The exception is the interaction of benzofuroxan **3** with diaminophenyl derivative **2f** containing diethylphosphonate fragment. We found that regardless of the ratio of reagents employed, there is only a formation of a mono-substitution product **4f.** The 2:1 product cannot be obtained, probably because of a sterically hindered environment for the NH_2_ group located in the *ortho*-position relative to the methyldiethylphosphonate fragment.

### 2.2. Biological Evaluation

For the synthesized compounds, diverse biological properties were studied: antitumor potential with the determination of the mechanisms of action, hemolytic activity, antibacterial properties and biostability.

#### 2.2.1. Anticancer Activity

To start, a new series of synthesized compounds proposed as potential anticancer agents were tested for cytotoxicity against cancer and normal cell lines (Table 1). Data on cytotoxic activity are represented by IC_50_ values (the concentration of compound that causes 50% cell death in the test population).

It is interesting to compare the activity of hybrid molecules **4**–**5** to their building blocks, SHPs **2** and chlorobenzofuroxan **3**. As can be seen from the data given in Table 1, the simple benzofuroxan **3** does not show cytotoxicity at these concentrations. At the same time, the initial SHPs **2** have cytotoxic activity, which, as we noted earlier, motivated us to choose them as a starting point of our design. Gratifying, the hybrid compounds **4**–**5** showed relatively high activity against all cancer lines used in the experiments. In addition, the lead compounds have moderate cytotoxicity against the normal Chang liver cells.

When compared with the starting compounds, the SHP/benzofuroxan hybrid **4c** is 40 times more active than SHP **2c** with respect to M-HeLa and 20 times more active with respect to MCF-7. The activity of compound **5d** exceeds the activity of compound **2d** by 3.7 times with respect to M-HeLa and 7.6 times with respect to MCF-7. However, it should be noted that the cytotoxicity towards normal cells of these hybrid leader compounds also increases, which makes these compounds more toxic. These data suggest that finding the proper balance between efficacy and selectivity remains a challenge. From these data, we have identified two lead compounds, **4c** and **5d**, that show high cytotoxicity against human duodenal adenocarcinoma (HuTu 80), human breast adenocarcinoma (MCF-7) and human cervical carcinoma cell lines. The IC_50_ values of compounds **4c** and **5d** for these lines were either comparable to or exceeded the activity of the reference drugs Doxorubicin and Sorafenib.

The key indicator for evaluating perspective antitumor drugs is the selectivity index (SI), which was calculated as the ratio between the IC_50_ value for normal cells and the IC_50_ value for cancer cells. The selectivity index values for **5d** are shown in Table 1. According to the literature guidelines [40], compounds are considered selective at SI ≥ 3. Therefore, the lead compound **5d** can be considered selective for MCF-7 and M-HeLa cell lines at SI = 4. Note that the reference drugs Doxorubicin and Sorafenib are significantly inferior in selectivity compared to the lead compound.

**Figure 2:**
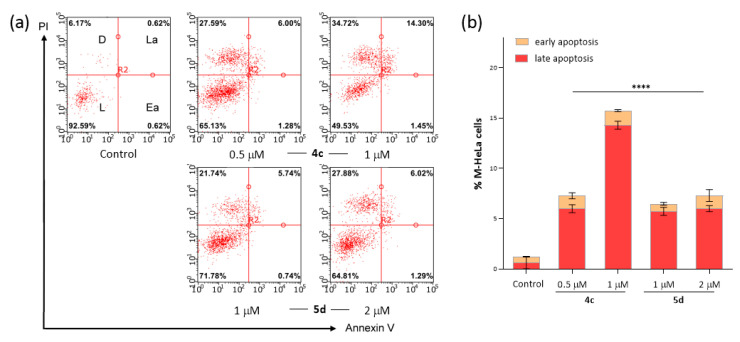
Induction of apoptosis in M-HeLa cancer cells by compounds **4c** and **5d**, evaluated by Flow Cytometry Assay, after cell labeling with propidium iodide (PI) and Annexin V- Alexa Fluor 647 (**a**). L, D, La and Ea labels in the top right control plot correspond to Live, Dead, Late apoptosis and Early apoptosis, respectively. Cells were either unlabeled and untreated (Control) or treated with IC_50_ and IC_50_/2 doses of test compounds for 48 h, as indicated. The quantitative analysis of early and late apoptotic cells after drug exposure is represented in (**b**). Data are presented as mean ± error of the mean (*n* = 3). **** *p* ≤ 0.0001 compared to control (one-way ANOVA). Concentration for **4c** was (IC_50_/2- 0.5 µM and IC_50_—1.0 µM); for **5d** (IC_50_/2—1.0 µM and IC_50_—2.0 µM).

Apoptosis is one of the preferred mechanisms of cytotoxic action for the development of new anticancer agents. Apoptosis-inducing properties of the leading compounds, **4c** and **5d,** were evaluated by flow cytometry at IC_50_/2 and IC_50_ concentrations on the M-HeLa cell line (Figure 2a,b). This assay is convenient for detecting apoptosis and for differentiating its stages. In particular, the viable cells are negative for both PI and Annexin V- Alexa Fluor 647 binding; non-viable, necrotic cells are negative for Annexin V- Alexa Fluor 647 binding and positive for PI uptake; cells in early apoptosis are Annexin V- Alexa Fluor 647 positive and PI negative; cells in late apoptosis are positive for both Annexin V- Alexa Fluor 647 binding and PI uptake.

It can be seen that after 48 h of incubation, the test compounds begin to induce apoptosis in M-HeLa cells. The apoptotic effects are most prominent in the case of the leading compound **4c**, especially at the stage of late apoptosis (Figure 2b).

**Figure 3:**
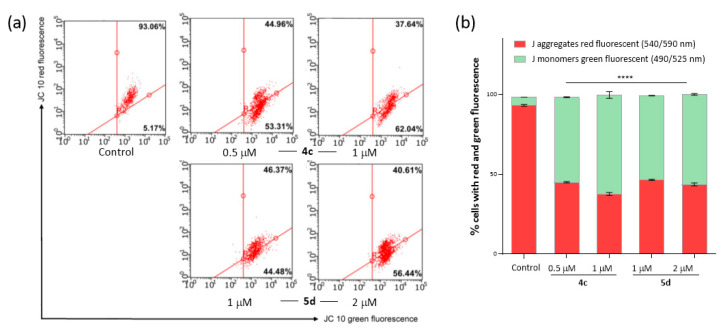
(**a**) Flow cytometry analysis of M-Hela cells treated with compounds **4c** and **5d**. (**b**) Quantitative determination of % cells with red aggregates and green monomers. Data are presented as mean ± error of the mean (*n* = 3). **** *p* ≤ 0.0001 compared to control (one-way ANOVA). Concentration for **4c** was (IC_50_/2—0.5 µM and IC_50_—1.0 µM); for **5d** (IC_50_/2—1.0 µM and IC_50_—2.0 µM).

The possibility of apoptosis through the mitochondrial pathway was assessed by flow cytometry using the JC-10 fluorescent dye (in the Mitochondria Membrane Potential Kit). In normal cells, JC-10 accumulates in the mitochondrial matrix, where it forms aggregates identified via their red fluorescence. However, in apoptotic cells, JC-10 diffuses out of the mitochondria, converts to its monomeric form, and emits green fluorescence, which is recorded by a flow cytometer. After treatment with compounds **4c** and **5d** at concentrations of IC_50_/2 and IC_50_, we observed the dissipation of the mitochondrial membrane potential of M-HeLa cells, which became more pronounced in the case of compound **4c** (Figure 3a). Figure 3b shows that the intensity of green fluorescence significantly increased relative to the control. These results suggest that the mechanism of action of the studied compounds is associated with the induction of apoptosis, which occurs along the internal mitochondrial pathway.

**Figure 4:**
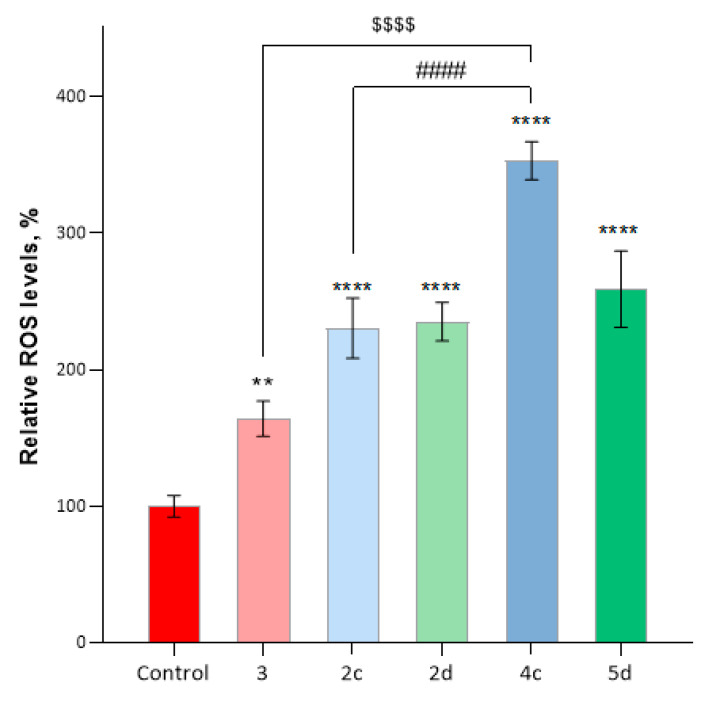
Induction of ROS production by compounds **2c**, **2d**, **3**, **4c** and **5d**. The data were obtained with flow cytometry and the CellROX^®^ Deep Red kit. Concentration for compounds was equivalent to IC_50_ cytotoxicity on M-HeLa cells. Data are presented as mean ± SD (*n* = 3). ** *p* < 0.01, **** *p* < 0.0001 compared to control; #### *p* ≤ 0.0001, compared to **2c**; $$$$ *p* ≤ 0.0001 and compared to **3** (one-way ANOVA).

Apoptosis can be induced in various ways, including an increase in the production of reactive oxygen species (ROS) in the cell, with subsequent oxidative stress and destruction of membrane lipids, proteins and nucleic acids. Thus, chemical compounds that disturb the redox balance and lead to the production and accumulation of ROS are potential agents for targeting the transformed cells. To understand the possible synergy between the different components of these hybrid agents, we compared the ROS production for compounds **4c**, **5d**, starting phenols **2c**, **2d** and furoxan **3** at the concentration of IC50 cytotoxicity on M-HeLa cells (Figure 4). A significant increase in CellROX^®^ Deep Red fluorescence intensity indicates an increase in ROS production. This increase is especially pronounced in the presence of compound **4c** where ROS production significantly exceeds that for the original phenol **2c** and furoxan **3**. Hence, it is reasonable to suggest that activity of compound **4c** benefits from the synergy between the methylene quinones (as producers of superoxide) and furoxans (as the NO donors).

Thus, the phenol-benzofuroxan hybrids exhibit high cytotoxicity. This biological activity is primarily associated with the induction of apoptosis, occurring via the internal mitochondrial pathway and an increase in ROS production.

Additionally, we evaluated the hemolytic activity (i.e., the ability to destroy human erythrocytes) [41,42,43]and biostability in whole mice blood for the leader compounds [44]. The tested compounds do not show hemolytic activity (HC_50_ >100 µM) and are metabolized relatively slowly (data are presented in supporting information, Figure S46).

#### 2.2.2. Antimicrobial Activity

The synthesized compounds were also tested for antibacterial activity against a number of gram-positive *Staphylococcus aureus* ATCC 6538P FDA 209P (*Sa*), *Bacillus cereus* ATCC 10702 (*Bc*), *Enterococcus faecalis* ATCC 29212 (*Ef*), gram-negative bacteria *Escherichia coli* ATCC 25922 (*Ec*) and *Pseudomonas aeruginosa* ATCC 9027 (*Pa*), including against methicillin-resistant strains of *Staphylococcus aureus MRSA-1* and *MRSA-2*. Methicillin-resistant strains of *S. aureus* were provided to us by the Republican Clinical Hospital (Kazan, Russia) from patients with chronic tonsillitis and sinusitis and were highly resistant: MRSA-1—to β-lactams and fluoroquinolones and MRSA-2—only to β-lactams. Antifungal activity was studied on *Candida albicans* 10231. Neither sterically hindered phenols **2** and benzofuroxan **3** nor their 1:1 hybrids **4** show activity against fungi and bacteria. On the other hand, the introduction of the second benzofuroxan fragment leads to the rise of antimicrobial activity (Table 2). Compounds **5a**, **5c** and **5d** display selective antimicrobial activity against gram-positive bacteria *S. aureus*, *B. cereus*, *E. faecalis* (at the level of the reference drug Chloramphenicol) and strain *MRSA 1*. These compounds are less active against *MRSA-2*. All studied compounds were inactive toward gram-negative bacteria and the yeast *Candida albicans* 10231.

## 3. Materials and Methods

### 3.1. Chemistry

IR spectra were recorded on an IR Fourier spectrometer Tensor 37 (Bruker Optik GmbH, Ettlingen, Germany) in the 400–3600 cm^−1^ range in KBr. The ^1^H- and ^13^C-NMR spectra were recorded on a Bruker AVANCE 400 spectrometer (Bruker BioSpin, Rheinstetten, Germany) operating at 400 MHz (for ^1^H NMR), 101 MHz (for ^13^C NMR) and 162 MHz (for ^31^P NMR) and Brucker spectrometers AVANCE*III*-500 (Bruker Corporation, Rheinstetten, Germany) operating at 500 MHz (for ^1^H NMR) and 126 MHz (for ^13^C MMR). Chemical shifts were measured in δ (ppm) with reference to the solvent (δ = 7.27 ppm and 77.00 ppm for CDCl_3_; δ = 2.06 ppm and 28.94 ppm for (CD_3_)_2_CO, δ = 2.56 ppm and 39.52 ppm for DMSO-d_6_ for ^1^H and ^13^C NMR, respectively). Elemental analysis was performed on a CHNS-O Elemental Analyser EuroEA3028-HT-OM (EuroVector S.p.A., Milan, Italy). ESI-TOF-MS spectra were recorded on a Bruker AmazonX instrument (Bruker Daltonix GmbH, Bremen, Germany). The melting points were determined on JK-MAM-4 Melting-point Apparatus with Microscope (JINGKE SCIENTIFIC INSTRUMENT CO, Shanghai, China). The progress of reactions and the purity of products were monitored by TLC on Sorbfil UV-254 plates (Sorbpolimer, Krasnodar, Russia); the chromatograms were developed under UV light.

7-Chloro-4,6-dinitrobenzofuroxan **3** was synthesized according to the literature [45].

Reaction between sterically hindered phenols **2a**–**g** and 7-chloro-4,6-dinitrobenzofuroxan **3**. To a solution of 7-chloro-4,6-dinitrobenzofuroxan **3** (0.8 mmol) in 5 mL of CHCl_3_ at room temperature was added a solution of sterically hindered phenols **2** (1.6 mmol (for compounds **4**) or 1.2 mmol (for compounds **5**)) in 5 mL of CHCl_3_. The reaction was carried out at room temperature and under magnetic stirring, and the conversion was monitored through TLC analysis (eluent: toluene/ethyl acetate, 2:1). The mixture was stirred at room temperature overnight; the crude mixture was precipitated in hexane (10 mL), the obtained solid was filtered off, washed with cold water (100 mL) and dried under vacuum (0.06 mm Hg) at 40 °C temperature to constant weight. In any case, a mixture of compounds **4** and **5** was obtained, depending on the conditions, with a high content of one of them. The crude product was purified by column chromatography (eluent in each case was selected individually) to give the target compound (the second product in this case was isolated in an insignificant amount).


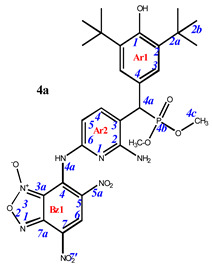


**7-((6-amino-5-((3,5-di-*tert*-butyl-4-hydroxyphenyl)(dimethoxyphosphoryl)methyl)pyridin-2-yl)amino)-4,6-dinitrobenzo[*c*][1,2,5]oxadiazole 1-oxide (4a).** Purple powder, yield 85%. M.p.: 270–271 °C. IR (*ν*, cm^–1^): 694 (P–C), 1228 (P=O), 1359 (NO_2_ symm), 1563 (NO_2_ asymm), 1623 (furoxan ring), 3448 (NH_2_) and 3621 (OH). ^1^H NMR (500 MHz, Acetone-*d*_6_): δ 9.10 (s, 1H, H6-Bz1), 8.13 (dd, *J* = 8.1 Hz, *J*(PH) = 1.5 Hz, 1H, H4-Ar2), 7.44 (d, *J*(PH) = 1.8 Hz, 2H, H3-Ar1), 6.73 (brs, 1H, H5-Ar2), 6.11 (s, 1H, OH-Ar1), 4.67 (d, *J*(PH) = 26.8 Hz, 1H, H4a-Ar1), 3.70 and 3.61 (d, *J* = 10.7 Hz, 6H, CH3) and 1.47 (s, 18H, H2b-Ar1). ^13^C{^1^H} NMR (126 MHz, Acetone-*d*_6_) δ 156.7 (C2-Ar2), 154.9 (C1-Ar1), 151.2 (C6-Ar2), 148.1 (C4-Bz), 143.7 (C4-Ar2), 139.1 (C2-Ar1), 139.0 (C7a-Bz), 134.4 (C6-Bz), 128.3 (C7-Bz), 127.7 (d, *J*(PC) = 7.3 Hz, C3-Ar1), 127.4 (C3-Ar2), 125.3 (C5-Bz), 115.0 (C3a-Bz), 114.0 (C4-Ar1), 104.9 (C5-Ar2), 54.5 and 54.2 (d, *J*(PC) = 7.2 Hz, CH3), 44.9 (d, *J*(PC) = 138.7 Hz, C4a-Ar1), 36.0 (C2a-Ar1) and 31.4 (C2b-Ar1). ^31^P NMR (162 MHz, Acetone-d_6_) δ 27.30. Found: C, 51.15; H, 5.24; N, 14.83; P, 4.77. Anal. calcd (%) for C_28_H_34_N_7_O_10_P: C, 50.99; H, 5.20; N, 14.87; P, 4.70. HRMS (ESI) *m/z* for C_28_H_34_N_7_O_10_P: calc. 659.21 [M]^+^, found 658.15 [M-H]^+^.


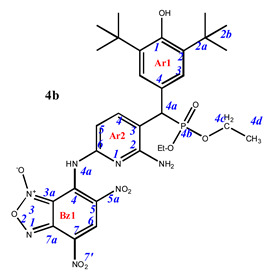


**7-**((**6-amino-5-**((**3,5-di-*tert*-butyl-4-hydroxyphenyl**)(**diethoxyphosphoryl**)**methyl**)**pyridin-2-yl**)**amino**)**-4,6-dinitrobenzo[c][1,2,5]oxadiazole 1-oxide** (**4b**)**.** Purple powder, yield 78%. M.p.: 142–143 °C. IR (*ν*, cm^–1^): 703 (P–C), 1226 (P=O), 1365 (NO_2_ symm), 1563 (NO_2_ asymm), 1620 (furoxan ring) and 3440 (NH_2_); 3616 (OH). Minor amounts of the other tautomer are present in the spectra. ^1^H NMR (500 MHz, Acetone-*d*_6_): δ 9.08 (s, 1H, H6-Bz1), 8.14 (dd, *J* = 8.1 Hz, *J*(PH) = 1.5 Hz, 1H, H4-Ar2), 7.44 (d, *J*(PH) = 1.8 Hz, 2H, H3-Ar1), 6.66 (brs, 1H, H5-Ar2), 4.63 (d, *J*(PH) = 26.9 Hz, 1H, H4a-Ar1), 4.06 (m, 4H, CH2), 1.47 (s, 18H, H2b-Ar1), 1.24 and 1.17 (tr, *J* = 7.2 Hz, 6H, CH3). ^13^C{^1^H} NMR (126 MHz, Acetone-*d*_6_) δ 156.1 (C2-Ar2), 154.7 (C1-Ar1), 151.8 (C6-Ar2), 148.1 (C4-Bz), 144.1 (C4-Ar2), 140.2 (C7a-Bz), 139.0 (C2/C6-Ar1), 133.9 (C6-Bz), 127.9 (C7-Bz), 127.8 (d, *J*(PC) = 7.3 Hz, C3-Ar1), 127.4 (C3-Ar2), 124.2 (C5-Bz), 115.0 (C3a-Bz), 114.0 (C4-Ar1), 104.9 (C5-Ar2), 64.2 and 53.9 (m, CH2), 45.0 (d, *J*(PC) = 138.7 Hz, C4a-Ar1), 35.9 (C2a-Ar1), 31.3 (C2b-Ar1) and 17.4 and 17.3 (m, CH3). ^31^P NMR (162 MHz, Acetone-d_6_) δ 25.01. Found: C, 52.45; H, 5.52; N, 14.33; P, 4.57. Anal. calcd (%) for C_30_H_38_N_7_O_10_P: C, 52.40; H, 5.57; N, 14.26; P, 4.50. HRMS (ESI) *m/z* for C_30_H_38_N_7_O_10_P: calc. 687.24 [M]^+^, found 686.18 [M-H]^+^.


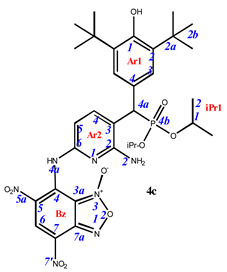


**7-**((**6-amino-5-**((**3,5-di-*tert*-butyl-4-hydroxyphenyl**)(**diisopropoxyphosphoryl**)**methyl**)**pyridin-2-yl**)**amino**)**-4,6-dinitrobenzo[*c*][1,2,5]oxadiazole 1-oxide** (**4c**)**.** Claret powder, yield 69%. M.p.: 119–120 °C. IR (*ν*, cm^–1^): 705 (P–C), 1206 (P=O), 1364 (NO_2_ symm), 1569 (NO_2_ asymm), 1621 (furoxan ring) and 3444 (NH_2_); 3620 (OH). ^1^H NMR (500 MHz, Acetone-d_6_): δ 11.34 (s, 1H, NH- or H4a-Bz), 9.08 (s, 1H, H6-Bz), 8.12 (dd, *J* = 8.2 Hz, *J*(PH) = 1.7 Hz, 1H, H4-Ar2), 7.46 (d, *J*(PH) = 1.8 Hz, 2H, H3-Ar1), 6.67 (d, *J* = 7.9 Hz, 1H, H5-Ar2), 6.12 (s, 1H, OH-Ar1), 4.76 (brs, 2H, NH2-Ar2), 4.68 and 4.57 (m, 8H, H1-iPr), 4.56 (d, *J*(PH) = 26.9 Hz, 1H, H4a-Ar1), 1.47 (s, 18H, H2b-Ar1), 1.30, 1.29, 1.16 and 0.99 (d, *J* = 6.2 Hz, 12H, H2-iPr). ^13^C{^1^H} NMR (126 MHz, Acetone-d_6_) δ 156.3 (d, *J*(PC) = 10.1 Hz, C2-Ar2), 154.8 (d, *J*(PC) = 14.3 Hz, C1-Ar1), 151.5 (C6-Ar2), 148.2 (C4-Bz), 143.8 (d, *J*(PC) = 6.9 Hz, C4-Ar2), 139.9 (C7a-Bz), 137.0 (d, *J*(PC) = 15.6 Hz, C2-Ar1), 133.7 (C6-Bz), 128.2 (d, *J*(PC) = 5.6 Hz, C4-Ar1), 128.0 (d, *J*(PC) = 7.5 Hz, C3-Ar1), 127.7 (C7-Bz), 124.1 (C5-Bz), 115.5 (C3-Ar2), 113.9 (C3a-Bz), 104.4 (C5-Ar2), 73.0 and 72.5 (d, *J*(PC) = 7.2 Hz, C1-iPr), 46.0 (d, *J*(PC) = 140.4 Hz, C4a-Ar1), 35.9 (C2a-Ar1), 31.3 (C2b-Ar1), 25.2, 25.0 24.6 and 24.4 (d, *J*(PC) = 5.4 Hz, C1-iPr). ^31^P NMR (162 MHz, Acetone-d_6_) δ 23.69. Found: C, 53.75; H, 5.86; N, 13.64; P, 4.22. Anal. calcd (%) for C_32_H_42_N_7_O_10_P: C, 53.70; H, 5.92; N, 13.70; P, 4.33. HRMS (ESI) *m/z* for C_32_H_42_N_7_O_10_P: calc. 715.27 [M]^+^, found 716.27 [M+H]^+^, 714.25 [M-H]^+^.


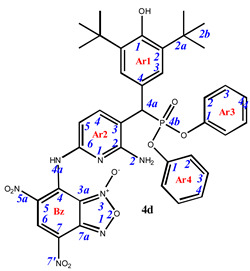


**7-**((**6-amino-5-**((**3,5-di-*tert*-butyl-4-hydroxyphenyl**)(**diphenoxyphosphoryl**)**methyl**)**pyridin-2-yl**)**amino**)**-4,6-dinitrobenzo[*c*][1,2,5]oxadiazole 1-oxide** (**4d**)**.** Dark cherry powder, yield 76%. M.p.: 156–157 °C. IR (*ν*, cm^–1^): 690 (P–C), 1211 (P=O), 1378 (NO_2_ symm), 1565 (NO_2_ asymm), 1592 (C=C_arom_), 1622 (furoxan ring), 3437 (NH_2_). ^1^H NMR (500 MHz, CDCl_3_): δ 10.69 (s, 1H, NH- or H4a-Bz), 9.18 (s, 1H, H6-Bz), 8.03 (d, *J* = 7.9 Hz, 1H, H4-Ar2), 7.27 (tr, *J* = 8.5 Hz, 2H, H3/H5-Ar4), 7.27 (s, 2H, H3-Ar1), 7.21 (tr, *J* = 8.5 Hz, 2H, H3/H5-Ar3), 7.16 (tr, *J* = 8.5 Hz, 1H, H4-Ar4), 7.10 (tr, *J* = 8.5 Hz, 1H, H4-Ar3), 6.95 (d, *J* = 8.5 Hz, 2H, H2/H6-Ar4 [Ar4 cis- to 4a of Ar1]), 6.80 (d, *J* = 8.5 Hz, 2H, H2/H6-Ar3), 6.63 (d, *J* = 7.9 Hz, 1H, H5-Ar2), 5.28 (s, 1H, OH-Ar1), 4.84 (s, 2H, NH2-Ar21), 4.61 (d, *J*(PH) = 26.7 Hz 1H, H4a-Ar1) and 1.40 (s, 18H, H2b-Ar1). ^13^C{^1^H} NMR (126 MHz, CDCl_3_) δ 156.5 (d, *J*(PC) = 10.1 Hz, C2-Ar2), 154.0 (C1-Ar1), 150.8 (d, *J*(PC) = 9.7 Hz, C1-Ar3), 150.3 (d, *J*(PC) = 9.7 Hz, C1-Ar4 [Ar4 cis- to 4a of Ar1] ), 148.2 (C6-Ar2), 145.8 (C4-Bz), 141.5 (d, *J*(PC) = 6.9 Hz, C4-Ar2), 137.0 (C2-Ar1), 136.1 (C7a-Bz), 130.8 (C6-Bz), 129.9 (C3-Ar3), 129.8 (C3-Ar4), 127.11 (C7-Bz), 126.5 (C5-Bz), 126.7 (d, *J*(PC) = 7.5 Hz, C3-Ar1), 125.9 (C4-Ar3), 125.3 (C4-Ar4), 123.3 (d, *J*(PC) = 5.6 Hz, C4-Ar1), 120.7 (d, *J*(PC) = 4.1 Hz, C2-Ar3), 120.3 (d, *J*(PC) = 4.1 Hz, C2-Ar4), 114.9 (C3-Ar2), 111.7 (C3a-Bz), 106.1 (C5-Ar2), 46.1 (d, *J*(PC) = 140.4 Hz, C4a-Ar1) and 34.6 (C2a-Ar1), 30.4 (C2b-Ar1). ^31^P NMR (162 MHz, CDCl_3_) δ 19.09. Found: C, 58.29; H, 4.81; N, 12.45; P, 3.89. Anal. calcd (%) for C_38_H_38_N_7_O_10_P: C, 58.24; H, 4.89; N, 12.51; P, 3.95. HRMS (ESI) *m/z* for C_38_H_38_N_7_O_10_P: calc. 783.24 [M]^+^, found 782.20 [M-H]^+^.


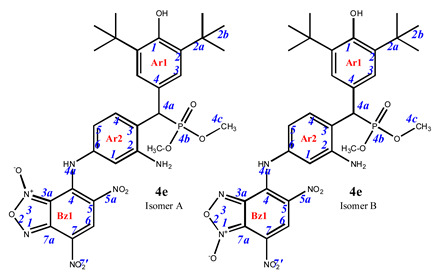


**7-**((**3-amino-4-**((**3,5-di-*tert*-butyl-4-hydroxyphenyl**)(**dimethoxyphosphoryl**)**methyl**)**phenyl**)**amino**)**-4,6-dinitrobenzo[*c*][1,2,5]oxadiazole 1-oxide** (**4e**)**.** Maroon powder, yield 72%. M.p.: 138–139 °C. IR (*ν*, cm^–1^): 681 (P–C), 1237 (P=O), 1376 (NO_2_ symm), 1569 (NO_2_ asymm), 1590 (C=C_arom_), 1621 (furoxan ring), 3415 (NH_2_) and 3625 (OH). Exists in solution as two isomers. Major isomer A (85%): ^1^H NMR (500 MHz, CDCl_3_): δ 11.17 (brs, NH-Ar2), 9.22 (s, 1H, H6-Bz1), 7.44 (dd, *J* = 8.2 Hz, *J*(PH) = 1.6 Hz, 1H, H4-Ar2), 7.22 (d, *J*(PH) = 1.5 Hz, 2H, H3-Ar1), 6.54 (d, *J* = 1.7 Hz, 1H, H1-Ar2), 6.50 (dd, *J* = 8.2 Hz, *J* = 1.7 Hz, 1H, H5-Ar2), 5.22 (s, 1H, OH-Ar1), 4.56 (d, *J*(PH) = 26.5 Hz, 1H, H4a-Ar1), 3.62 and 3.66 (d, *J* = 10.7 Hz, 6H, CH3) and 1.43 (s, 18H, H2b-Ar1). ^13^C{^1^H} NMR (126 MHz, CDCl_3_) δ 153.5 (C1-Ar1), 146.8 (C2-Ar2), 146.0 (C4-Bz), 138.1 (C7a-Bz), 137.8 (C6-Ar2), 136.4 (C2-Ar1), 132.3 (d, *J*(PC) = 6.3 Hz, C4-Ar2), 131.4 (C6-Bz), 126.5 (d, *J*(PC) = 7.6 Hz, C3-Ar1), 125.3 (C7-Bz), 125.0 (d, *J*(PC) = 5.2 Hz, C4-Ar1), 124.5 (C5-Bz), 123.6 (C3-Ar2), 112.0 (C5-Ar2), 110.2 (C1-Ar2), 109.9 (C3a-Bz), 54.0 and 53.5 (d, *J*(PC) = 7.1 Hz, CH3), 45.6 (d, *J*(PC) = 139.2 Hz, C4a-Ar1), 34.6 (C2a-Ar1) and 30.5 (C2b-Ar1). ^31^P NMR (162 MHz, CDCl_3_) δ 27.28. Isomer B (15%), since the concentration is low, only part of the peaks can be correlated: ^1^H NMR (500 MHz, CDCl_3_): δ 11.49 (s, NH-Ar2), 8.93 (s, 1H, H6-Bz1), 7.51 (dd, *J* = 8.2 Hz, *J*(PH) = 1.6 Hz, 1H, H4-Ar2), 7.25 (d, *J*(PH) = 1.5 Hz, 2H, H3-Ar1), 6.76 (d, *J* = 1.7 Hz, 1H, H1-Ar2), 6.73 (dd, *J* = 8.2 Hz, *J* = 1.7 Hz, 1H, H5-Ar2), 5.22 (s, 1H, OH-Ar1), 4.61 (d, *J*(PH) = 26.5 Hz, 1H, H4a-Ar1), 3.66 and 3.62 (d, *J* = 10.7 Hz, 6H, CH3) and 1.43 (s, 18H, H2b-Ar1). ^31^P NMR (162 MHz, CDCl_3_) δ 28.27. Found: C, 52.95; H, 5.29; N, 12.68; P, 4.65. Anal. calcd (%) for C_29_H_35_N_6_O_10_P: C, 52.89; H, 5.36; N, 12.76; P, 4.70. HRMS (ESI) *m/z* for C_29_H_35_N_6_O_10_P: calc. 658.22 [M]^+^ and found 657.16 [M-H]^+^.


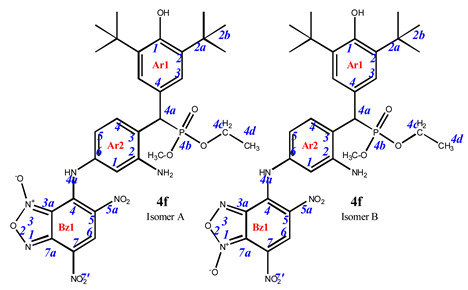


**7-**((**3-amino-4-**((**3,5-di-*tert*-butyl-4-hydroxyphenyl**)(**diethoxyphosphoryl**)**methyl**)**phenyl**)**amino**)**-4,6-dinitrobenzo[*c*][1,2,5]oxadiazole 1-oxide** (**4f**)**.** Dark cherry powder, yield 78%. M.p.: 201–202 °C. IR (*ν*, cm^–1^): 695 (P–C), 1239 (P=O), 1376 (NO_2_ symm), 1570 (NO_2_ asymm), 1621 (furoxan ring), 3250 (NH_2_) and 3439 (OH). Exists in solution as two isomers. Major isomer A (94%): ^1^H NMR (400 MHz, DMSO-d_6_): δ 8.91 (s, 1H, H6-Bz1), 7.54 (dd, *J* = 8.4 Hz, *J*(PH) = 1.4 Hz, 1H, H4-Ar2), 7.24 (d, *J*(PH) = 1.8 Hz, 2H, H3-Ar1), 6.61 (s, 1H, H1-Ar2), 6.55 (dd, *J* = 8.3 Hz, *J* = 1.7 Hz, 1H, H5-Ar2), 4.63 (d, *J*(PH) = 26.7 Hz, 1H, H4a-Ar1), 3.86 (m, 6H, CH3), 1.35 (s, 18H, H2b-Ar1) and 1.09 (m, 4H, CH2). ^13^C NMR (101 MHz, DMSO-d_6_) δ 153.6, 147.9, 139.6, 139.0, 138.3, 133.9, 131.4 (d, *J*(PC) = 5.5 Hz), 129.9, 129.2, 127.6 (d, *J*(PC) = 5.3 Hz), 127.0 (d, *J*(PC) = 7.0 Hz), 126.4 (d, *J*(PC) = 13.4 Hz), 121.4, 112.8, 110.6, 109.8, 62.9 (dd, *J*(PC) = 20.3, 6.8 Hz), 35.6, 31.4, 22.0 and 17.1 (d, *J*(PC) = 3.5 Hz). ^31^P NMR (162 MHz, DMSO-d_6_) δ 27.89. Found: C, 53.62; H, 5.49; N, 12.45; P, 4.69. Anal. calcd (%) for C_30_H_37_N_6_O_10_P: C, 53.57; H, 5.54; N, 12.49; P, 4.60.


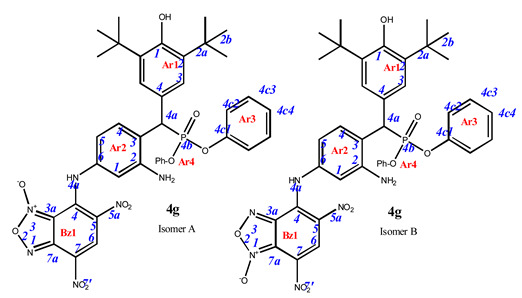


**7-**((**3-amino-4-**((**3,5-di-*tert*-butyl-4-hydroxyphenyl**)(**diphenoxyphosphoryl**)**methyl**)**phenyl**)**amino**)**-4,6-dinitrobenzo[*c*][1,2,5]oxadiazole 1-oxide** (**4g**)**.** Purple powder, yield 81%. M.p.: 139–140 °C. IR (*ν*, cm^–1^): 689 (P–C), 1238 (P=O), 1377 (NO_2_ symm), 1567 (NO_2_ asymm), 1590 (C=C_arom_), 1620 (furoxan ring), 3437 (NH_2_) and 3625 (OH). Exists in solution as two isomers. Isomer A (80%): ^1^H NMR (500 MHz, CDCl_3_): δ 11.12 (br.s, NH-Ar2), 9.22 (s, 1H, H6-Bz1), 7.67 (dd, *J* = 8.2 Hz, *J*(PH) = 1.6 Hz, 1H, H4-Ar2), 7.31 (d, *J*(PH) = 1.5 Hz, 2H, H3-Ar1), 7.26 and 7.22 (tr, *J* = 8.0 Hz, 4H, H3/H5 -Ar3 and Ar4), 7.10 and 7.15 (tr, *J* = 8.0 Hz, 2H, H2/H6-Ar3 and Ar4), 6.92 and 6.82 (d, *J* = 8.0 Hz, 4H, H2/H6-Ar3 and Ar4), 6.54 (d, *J* = 1.7 Hz, 1H, H1-Ar2), 6.53 (dd, *J* = 8.2 Hz, *J* = 1.7 Hz, 1H, H5-Ar2), 5.25 (s, 1H, OH-Ar1), 4.84 (d, *J*(PH) = 26.4 Hz, 1H, H4a-Ar1), 1.40 (s, 18H, H2b-Ar1). ^13^C{^1^H} NMR (126 MHz, CDCl_3_) δ 153.8 (C1-Ar1), 150.7 and 150.4 (d, *J*(PC) = 9.7 Hz, C4c1 -Ar3 and Ar4), 147.1 (C2-Ar2), 146.0 (C4-Bz), 138.0 (C7a-Bz), 138.0 (C6-Ar2), 136.7 (C2-Ar1), 132.2 (d, *J*(PC) = 6.4 Hz, C4-Ar2), 131.4 (C6-Bz), 129.9 and 129.8 (C4c3/C4c5 -Ar3 and Ar4), 127.1 (d, *J*(PC) = 9.5 Hz, C3-Ar1), 125.5 and 125.2 (C4c4 -Ar3 and Ar4), 125.3 (C7-Bz), 125.0 (C5-Bz),124.1 (d, *J*(PC) = 5.2 Hz, C4-Ar1), 122.9 (C3-Ar2), 120.8 and 120.4 (d, *J*(PC) = 4.2 Hz, C4c2/C4c6 -Ar3 and Ar4), 112.0 (C5-Ar2), 110.2 (C1-Ar2), 110.0 (C3a-Bz), 45.9 (d, *J*(PC) = 139.2 Hz, C4a-Ar1), 34.6 (C2a-Ar1) and 30.4 (C2b-Ar1). ^31^P NMR (162 MHz, CDCl_3_) δ 18.50. Isomer B (20%), since the concentration is low, only part of the peaks can be correlated: ^1^H NMR (500 MHz, CDCl_3_): δ 11.48 (brs, NH-Ar2), 8.93 (s, 1H, H6-Bz1), 7.76 (dd, *J* = 8.2 Hz, *J*(PH) = 1.6 Hz, 1H, H4-Ar2), 7.33 (d, *J*(PH) = 1.5 Hz, 2H, H3-Ar1), 7.26 and 7.22 (tr, *J* = 8.0 Hz, 4H, H3/H5 -Ar3 and Ar4), 7.15 and 7.10 (tr, *J* = 8.0 Hz, 2H, H2/H6-Ar3 and Ar4), 6.92 and 6.82 (d, *J* = 8.0 Hz, 4H, H2/H6-Ar3 and Ar4), 6.79 (dd, *J* = 8.2 Hz, *J* = 1.7 Hz, 1H, H5-Ar2), 6.74 (d, *J* = 1.7 Hz, 1H, H1-Ar2), 5.19 (s, 1H, OH-Ar1), 4.88 (d, *J*(PH) = 26.4 Hz, 1H, H4a-Ar1) and 1.40 (s, 18H, H2b-Ar1). ^31^P NMR (162 MHz, CDCl_3_) δ 20.09. Found: C, 59.91; H, 4.95; N, 10.69; P, 4.01. Anal. calcd (%) for C_39_H_39_N_6_O_10_P: C, 59.84; H, 5.02; N, 10.74; P, 3.96. HRMS (ESI) *m/z* for C_39_H_39_N_6_O_10_P: calc. 782.25 [M]^+^ and found 781.21 [M-H]^+^.


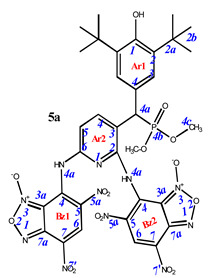


**7,7’-**((**3-**((**3,5-di-*tert*-butyl-4-hydroxyphenyl**)(**dimethoxyphosphoryl**)**methyl**)**pyridine-2,6-diyl**)**bis**(**azanediyl**))**bis**(**4,6-dinitrobenzo[*c*][1,2,5]oxadiazole 1-oxide**) (**5a**)**.** Bright red powder, yield 91%. M.p.: 160–161 °C. IR (*ν*, cm^–1^): 689 (P–C), 1213 (P=O), 1378 (NO_2_ symm), 1575 (NO_2_ asymm), 1594 (C=Carom), 1626 (furoxan ring), 3437 (NH_2_), 3632 (OH). ^1^H NMR (500 MHz, Acetone-d_6_): δ 11.05 (s, 2H, NH or H4a -Bz1 and -Bz2), 9.08 (s, 1H, H6-Bz2), 8.61 (s, 1H, H6-Bz1), 8.36 (d, *J* = 8.4 Hz, 1H, H4-Ar2), 7.58 (2H, H3-Ar1), 7.10 (brs, 1H, H5-Ar2), 5.94 (s, 1H, OH-Ar1), 5.65 (d, *J*(PH) = 12.4 Hz, 1H, H4a-Ar1), 3.66 and 3.61 (d, *J* = 10.3 Hz, 6H, CH3), 1.46 (s, 18H, H2b-Ar1). ^13^C{^1^H} NMR (Acetone-d_6_, 126 MHz) δ 157.4 (C2-Ar2), 157.3 (C6-Ar2), 154.5 (C1-Ar1), 149.5 (C4-Bz2), 148.1 (C4-Bz1), 141.6 (C4-Ar2), 141.5 (C7a-Bz1), 138.8 (C2-Ar1), 138.7 (C7a-Bz1), 134.4 (C6-Bz1), 132.8 (C6-Bz2), 130.0 (C5-Bz1), 129.8 (C4-Ar1), 129.5 (C5-Bz2), 128.6 (C7-Bz1), 128.0 (C7-Bz2), 127.9 (d, *J*(PC) = 7.5 Hz, C3-Ar1), 115.7 (C5-Ar2), 114.4 (C3a-Bz1), 113.7 (C3a-Bz2), 111.0 (C3-Ar2), 54.4 (CH3), 43.2 (d, *J*(PC) = 140.0 Hz, C4a-Ar1), 35.9 (C2a-Ar1), 31.4 (C2b-Ar1). ^31^P NMR (162 MHz, CDCl_3_) δ 27.44. Found: C, 46.27; H, 3.84; N, 17.48; P, 3.49. Anal. calcd (%) for C_34_H_34_N_11_O_16_P: C, 46.21; H, 3.88; N, 17.44; P, 3.51. HRMS (ESI) *m/z* for C_34_H_34_N_11_O_16_P: calc. 883.19 [M]^+^, found 882.12 [M-H]^+^.


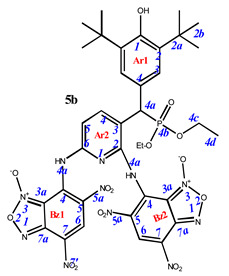


**7,7’-**((**3-**((**3,5-di-*tert*-butyl-4-hydroxyphenyl**)(**diethoxyphosphoryl**)**methyl**)**pyridine-2,6-diyl**)**bis**(**azanediyl**))**bis**(**4,6-dinitrobenzo[*c*][1,2,5]oxadiazole 1-oxide**) (**5b**)**.** Red powder, yield 88%. M.p.: >250 °C. IR (*ν*, cm^–1^): 696 (P–C), 1211 (P=O), 1378 (NO_2_ symm), 1571 (NO_2_ asymm), 1624 (furoxan ring) and 3436 (OH). Minor amounts of the other tautomer are present in the spectra. ^1^H NMR (500 MHz, Acetone-*d*_6_): δ 9.05 (s, 1H, H6-Bz2), 8.63 (brs, 1H, H6-Bz1), 8.40 (m, 1H, H4-Ar2), 7.54 (d, *J*(PH) = 0.6 Hz, 2H, H3-Ar1), 7.07 (d, *J* = 8.4 Hz, 1H, H5-Ar2), 5.95 (s, 1H, OH-Ar1), 5.53 (d, *J*(PH) = 25.3 Hz, 1H, H4a-Ar1), 4.02 (m, 4H, H4c-Ar1), 1.45 (s, 18H, H2b-Ar1) and 1.33 and 1.20 (d, *J* = 6.6 Hz, 6H, CH3 or H4d-Ar1). ^13^C{^1^H} NMR (Acetone-*d*_6_, 126 MHz) δ 154.9 (C1-Ar1), 149.6 (C2-Ar2), 149.5 (C6-Ar2), 148.2 (C4-Bz2 and C4-Bz1), 142.5 (d, *J*(PC) = 4.7 Hz, C4-Ar2), 141.8 (C7a-Bz1), 138.7 (C2-Ar1), 138.6 (C7a-Bz1), 134.5 (C6-Bz2), 1330 (C6-Bz1), 129.4 (C5-Bz1), 128.4 (C5-Bz2), 128.3 (C7-Bz1), 128.2 (C7-Bz2), 128.0 (d, *J*(PC) = 8.0 Hz, C3-Ar1), 127.1 (d, *J*(PC) = 4.3 Hz, C4-Ar1), 115.7 (d, *J*(PC) = 5.7 Hz, C3-Ar2), 114.3 (C3a-Bz1), 113.6 (C3a-Bz2), 111.0 (C5-Ar2), 63.7 (m, C4c-Ar1), 44.0 (d, *J*(PC) = 140.3 Hz, C4a-Ar1), 35.9 (C2a-Ar1), 31.4 (C2b-Ar1) and 17.3 (m, C4d-Ar1). ^31^P NMR (162 MHz, Acetone-*d*_6_) δ 25.97. Found: C, 47.48; H, 4.27; N, 16.88; P, 3.45. Anal. calcd (%) for C_36_H_38_N_11_O_16_P: C, 47.43; H, 4.20; N, 16.90; P, 3.40. HRMS (ESI) *m/z* for C_36_H_38_N_11_O_16_P: calc. 911.22 [M]^+^ and found 910.16 [M-H]^+^.


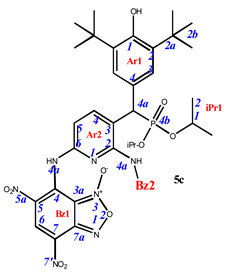


**7,7’-**((**3-**((**3,5-di-*tert*-butyl-4-hydroxyphenyl**)(**diisopropoxyphosphoryl**)**methyl**)**pyridine-2,6-diyl**)**bis**(**azanediyl**))**bis**(**4,6-dinitrobenzo[*c*][1,2,5]oxadiazole 1-oxide**) (**5c**)**.** Brick red powder, yield 78%. M.p.: >250 °C. IR (*ν*, cm^–1^): 695 (P–C), 1210 (P=O), 1360 (NO_2_ symm), 1565 (NO_2_ asymm), 1625 (furoxan ring) and 3444 (OH). ^1^H NMR (500 MHz, Acetone-d_6_): δ 11.30 (s, 1H, NH- or H4a-Bz2), 10.64 (s, 1H, NH- or H4a-Bz1), 9.07 (s, 1H, H6-Bz2), 8.90 (s, 1H, H6-Bz1), 8.15 (dd, *J* = 8.4 Hz, *J*(PH) = 1.0 Hz, 1H, H4-Ar2), 7.56 (d, *J*(PH) = 0.6 Hz, 2H, H3-Ar1), 7.53 (d, *J* = 8.4 Hz, 1H, H5-Ar2), 6.22 (s, 1H, OH-Ar1), 4.98 (d, *J*(PH) = 25.3 Hz, 1H, H4a-Ar1), 4.83 and 4.68 (sept, *J*(PH) = 6.3 Hz, 8H, H1-iPr), 1.49 (s, 18H, H2b-Ar1), 1.40, 1.38, 1.18 and 1.09 (d, *J* = 6.2 Hz, 12H, H2-iPr). ^13^C{^1^H} NMR (126 MHz, Acetone-d_6_,) δ 155.0 (C1-Ar1), 149.9 (C2-Ar2), 149.8 (C6-Ar2), 147.4 (C4-Bz2), 147.3 (C4-Bz1), 144.0 (d, *J*(PC) = 4.7 Hz, C4-Ar2), 139.5 (C7a-Bz1), 139.3 (C2-Ar1), 138.3 (C7a-Bz1), 132.1 (C6-Bz2), 132.0 (C6-Bz1), 130.9 (C5-Bz1), 130.0 (C5-Bz2), 129.7 (C7-Bz1), 128.4 (d, *J*(PC) = 8.0 Hz, C3-Ar1), 128.2 (C7-Bz2), 127.1 (d, *J*(PC) = 4.3 Hz, C4-Ar1), 125.9 (d, *J*(PC) = 5.7 Hz, C3-Ar2), 115.2 (C5-Ar2), 114.2 (C3a-Bz1), 113.6 (C3a-Bz2), 73.5 and 73.0 (d, *J*(PC) = 7.0 Hz, C1-iPr), 46.5 (d, *J*(PC) = 140.2 Hz, C4a-Ar1), 36.0 (C2a-Ar1), 31.3 (C2b-Ar1), 25.2, 25.0, 24.7 and 24.4 (d, *J*(PC) = 5.3 Hz, C1-iPr). ^31^P NMR (162 MHz, Acetone-*d*_6_) δ 24.34. Found: 48.54; H, 4.54; N, 16.32; P, 3.35. Anal. calcd (%) for C_38_H_42_N_11_O_16_P: C, 48.57; H, 4.50; N, 16.39; P, 3.30. HRMS (ESI) *m/z* for C_38_H_42_N_11_O_16_P: calc. 939.25 [M]^+^ and found 938.24 [M-H]^+^.


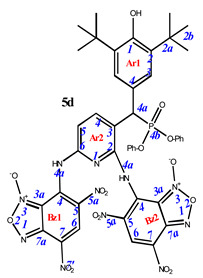


**7,7’-**((**3-**((**3,5-di-*tert*-butyl-4-hydroxyphenyl**)(**diphenoxyphosphoryl**)**methyl**)**pyridine-2,6-diyl**)**bis**(**azanediyl**))**bis**(**4,6-dinitrobenzo[*c*][1,2,5]oxadiazole 1-oxide**) (**5d**)**.** Brick red powder, yield 75%. M.p.: >250 °C. IR (*ν*, cm^–1^): 695 (P–C), 1209 (P=O), 1378 (NO_2_ symm), 1569 (NO_2_ asymm), 1622 (furoxan ring) and 3444 (OH). ^1^H NMR (400 MHz, DMSO-*d*_6_) δ 8.87 (s, 1H), 8.67 (s, 1H), 8.34 (d, *J* = 8.4 Hz, 1H), 8.28 (s, 1H), 7.30 (d, *J* = 8.1 Hz, 2H), 7.17 (m, 5H), 7.07 (m, 1H), 6.99 (d, *J* = 8.3 Hz, 2H), 6.87 (br.s, 1H), 6.64 (d, *J* = 8.2 Hz, 2H), 5.26 (m, 1H) and 1.24 (s, 18H). ^13^C NMR (126 MHz, DMSO-*d*_6_) δ 162.8, 161.7, 153.8, 150.4, 148.4, 147.7, 139.6, 135.2, 134.6, 130.4, 130.2, 130.2, 130.1, 129.9, 129.8, 127.6, 126.6, 125.6, 125.3, 120.9, 120.8, 120.4, 115.1, 111.9, 111.4, 36.2, 34.9 and 30.5.^31^P NMR (162 MHz, DMSO-d_6_) δ 18.80. Found: %: C, 52.49; H, 3.77; N, 15.35; P, 3.01. Anal. calcd (%) for C_44_H_38_N_11_O_16_P: C, 52.44; H, 3.80; N, 15.29; P, 3.07.


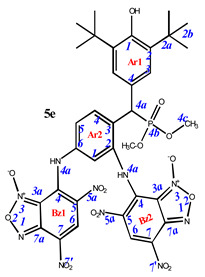


**7,7’-**((**4-**((**3,5-di-*tert*-butyl-4-hydroxyphenyl**)(**dimethoxyphosphoryl**)**methyl**)**-1,3-phenylene**)**bis**(**azanediyl**))**bis**(**4,6-dinitrobenzo[*c*][1,2,5]oxadiazole 1-oxide**) (**5e**)**.** Red powder, yield 68%. M.p.: >250 °C. IR (*ν*, cm^–1^): 689 (P–C), 1212 (P=O), 1378 (NO_2_ symm), 1578 (NO_2_ asymm), 1597 (C=Carom), 1624 (furoxan ring) and 3449 (OH). ^1^H NMR (400 MHz, DMSO-*d*_6_) δ 9.00 (s, 1H, H6-Bz2), 8.91 (s, 1H, H6-Bz1), 7.32 (s, 2H, H3-Ar1), 7.18 (s, 1H, Ar2), 7.09 (s, 1H, Ar2), 6.90 (s, 1H, Ar2), 5.07 (d, *J*(PH) = 24.4 Hz, 1H, H4a-Ar1), 3.62 and 3.58 (d, *J* = 10.4 Hz, 6H, CH3) and 1.35 (s, 18H, H2b-Ar1). ^13^C{^1^H} NMR (DMSO-*d*_6_, 126 MHz) δ 161.8, 153.6, 153.3, 148.5, 147.3, 147.3, 139.7, 139.5, 139.4 (d, *J*(PC) = 7.7 Hz), 138.1, 135.3, 132.9, 131.1, 127.7, 126.9, 126.9, 126.8, 126.6, 126.4, 115.2, 112.6, 111.5, 53.5 (d, *J*(PC) = 35.4 Hz), 35.1, 30.9 (d, *J*(PC) = 8.1 Hz) and 30.6. ^31^P NMR (162 MHz, DMSO-*d*_6_) δ 28.65. Found: C, 47.69; H, 4.07; N, 15.82; P, 3.56. Anal. calcd (%) for C_35_H_35_N_10_O_16_P: C, 47.63; H, 4.00; N, 15.87; P, 3.51. HRMS (ESI) *m/z* for C_35_H_35_N_10_O_16_P: calc. 882.20 [M]^+^ and found 881.12 [M-H]^+^.


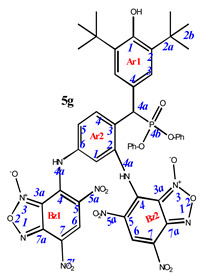


**7,7’-**((**4-**((**3,5-di-*tert*-butyl-4-hydroxyphenyl**)(**diphenoxyphosphoryl**)**methyl**)**-1,3-phenylene**)**bis**(**azanediyl**))**bis**(**4,6-dinitrobenzo[*c*][1,2,5]oxadiazole 1-oxide**) (**5g**)**.** Red powder, yield 55%. M.p.: 196–197 °C. Minor amounts of the other tautomer are present in the spectra. ^1^H NMR (600 MHz, DMSO-*d*_6_) δ 9.06 (s, 2H), 7.83 (dd, *J* = 8.3, *J* (PH) = 1.5 Hz, 1H), 7.39 (s, 2H), 7.32 (m, 2H), 7.19 (m, 4H), 7.10 (m, 1H), 6.93 (d, *J* = 8.5 Hz, 2H), 6.63 (m, 2H), 6.59 (d, *J* = 8.3 Hz, 1H), 5.18 (d, *J*(PH) = 27.9 Hz, 1H) and 1.31 (s, 18H). ^13^C NMR (101 MHz, DMSO-*d*_6_) δ 162.2, 154.3, 151.3 (d, *J* = 9.7 Hz), 150.9 (d, *J* = 10.3 Hz), 148.9, 140.1, 135.8, 131.8 (d, *J* = 5.2 Hz), 130.7, 130.5, 130.4, 130.3, 128.1, 127.3 (d, *J* = 7.8 Hz), 126.2, 126.1, 125.8, 121.4 (d, *J* = 3.7 Hz), 121.3, 121.1 (d, *J* = 3.7 Hz), 120.5, 116.2, 115.7, 111.9, 111.5, 110.4, 35.5 and 31.2. ^31^P NMR (162 MHz, DMSO-*d*_6_) δ 20.38. Found: C, 53.59; H, 3.88; N, 13.90; P, 3.05. Anal. calcd (%) for C_45_H_39_N_10_O_16_P: C, 53.68; H, 3.90; N, 13.91; P, 3.08. HRMS (ESI) *m/z* for C_45_H_39_N_10_O_16_P: calc. 1006.23 [M]^+^ and found 1005.19 [M-H]^+^.

### 3.2. Biology

#### 3.2.1. Cells and Materials

For the experiments, we used tumor cell cultures: M-HeLa clone 11 (epithelioid carcinoma of the cervix, subline HeLa., clone M-HeLa); T 98G—human glioblastoma; PANC-1, human pancreatic carcinoma; HuTu 80, human duodenal adenocarcinoma; MCF7—human breast adenocarcinoma (pleural fluid); A549, human lung carcinoma; WI38, VA 13 subline 2RA, human embryonic lung from the collection of the Institute of Cytology, Russian Academy of Sciences (St. Petersburg); PC3—prostate adenocarcinoma cell line from ATCC (American Type Cell Collection, USA; CRL 1435; human liver cells (Chang liver) from the collection and the Research Institute of Virology of the Russian Academy of Medical Sciences (Moscow). The cells were cultured in a standard Eagle’s nutrient medium manufactured at the Chumakov Institute of Poliomyelitis and Virus Encephalitis (PanEco company, Moscow, Russia), and supplemented with 10% fetal calf serum (Biosera, France) and 1% nonessential amino acids (PanEco company, Russia).

#### 3.2.2. Cytotoxicity Assay

The cytotoxic effect on cells was determined using the colorimetric method of cell proliferation—the MTT test. NADP-H-dependent cellular oxidoreductase enzymes can, under certain conditions, reflect the number of viable cells. These enzymes are able to reduce the tetrazolium dye, (MTT)—3-(4,5-dimethylthiazol-2-yl)-2,5-diphenyl-tetrazolium bromide, to insoluble blue-violet formazan, which crystallizes inside the cell. The amount of formazan formed is proportional to the number of cells with active metabolism [46]. Cells were seeded on a 96-well Nunc plate at a concentration of 5 × 103 cells per well in a volume of 100 μL of medium and cultured in a CO_2_ incubator at 37 °C until a monolayer was formed. The process of cell monolayer formation took 24 h. Then, the nutrient medium was removed, and 100 µL of solutions of the test drug in the given dilutions were added to the wells, which were prepared directly in the nutrient medium with the addition of 5% DMSO to improve solubility. After 48 h of incubation of the cells with the tested compounds, the nutrient medium was removed from the plates, and 100 µL of the nutrient medium without serum with MTT at a concentration of 0.5 mg/mL was added and incubated for 4 h at 37 °C. Formazan crystals were added 100 µL of DMSO to each well. Optical density was recorded at 540 nm on an Invitrologic microplate reader (Russia). The experiments for all compounds were repeated three times.

#### 3.2.3. Flow Cytometry Assay

**Cell Culture.** M-HeLa cells at 1 × 10^6^ cells/well in a final volume of 2 mL were seeded into six-well plates. After 48 h of incubation, various concentrations of compounds **4c** and **5d** were added to wells.

**Cell Apoptosis Analysis.** The cells were harvested at 2000 rpm for 5 min and, then washed twice with ice-cold PBS, followed by resuspension in binding buffer. Next, the samples were incubated with 5 μL of annexin V- Alexa Fluor 647 (Sigma-Aldrich, St. Louis, MO, USA) and 5 μL of propidium iodide for 15 min at room temperature in the dark. Finally, the cells were analyzed by flow cytometry (Guava easy Cyte, Merck, Rahway, NJ, USA) within 1 h. The experiments were repeated three times.

**Mitochondrial Membrane Potential.** Cells were harvested at 2000 rpm for 5 min and then washed twice with ice-cold PBS, followed by resuspension in JC-10 (10 µg/mL) and incubation at 37 °C for 10 min. After the cells were rinsed three times and suspended in PBS, the JC-10 fluorescence was observed by flow cytometry (Guava easy Cyte, Merck, Rahway, NJ, USA).

**Detection of Intracellular ROS.** M-HeLa cells were incubated with compounds at concentrations of IC_50_ for 48 h. ROS generation was investigated using flow cytometry assay and CellROX^®^ Deep Red flow cytometry kit. For this M-HeLa cells were harvested at 2000 rpm for 5 min and then washed twice with ice-cold PBS, followed by resuspension in 0.1 mL of medium without FBS, to which was added 0.2 μL of CellROX^®^ Deep Red and incubated at 37 °C for 30 min. After three times washing the cells and suspending them in PBS, the production of ROS in the cells was immediately monitored using flow cytometer (Guava easy Cyte, Merck, Rahway, NJ, USA).

#### 3.2.4. Antimicrobial Activity

Antimicrobial activity of test compounds was determined by serial micro dilutions in 96-well plates using Mueller-Hinton broth for bacterial culture and Sabouraud broth for yeast culture [47]. Cultures of gram-positive bacteria were used in the experiment: *Staphylococcus aureus* ATCC 6538 P FDA 209P, *Bacillus cereus* ATCC 10702 NCTC 8035, *Enterococcus faecalis* ATCC 29212; Gram-negative bacteria: *Escherichia coli* ATCC 25922, *Pseudomonas aeruginosa* ATCC 9027 and yeast: *Candida albicans* ATCC 10231, purchased from the State Collection of Pathogenic Microorganisms and Cell Cultures “SCPM-Obolensk”. Methicillin-resistant strains of *S. aureus* (*MRSA*) were isolated from patients with chronic tonsillitis *(MRSA-1*) and sinusitis (*MRSA-2*) in the bacteriological laboratory of the Republican Clinical Hospital (Kazan, Russia). The experiments were carried out in triplicate.

#### 3.2.5. Statistical Analysis

IC_50_ values were estimated using the Quest Graph IC50 Calculator (AAT Bioquest, Inc., Sunnyvale, CA, USA) (Version 2022) (accessed on 25 June 2022) [48].

### 3.3. Biostability Studies

#### 3.3.1. Preparation of Spikes and Samples

A substance of 1 mg was dissolved in methanol to achieve the solution of 1 mg/mL. By a series of further dilutions, a sample containing 5000 ng/mL in the same solvent was prepared. A total of 50 μL of the prepared solution was added to 450 μL of whole blood, resulting in a spike containing 500 ng/mL of the compound in the matrix.

Aliquot of 10 μL was taken from the spike and added to a 100 μL of the precipitation solution consisting of a mixture of 0.2 M ZnSO_4_ in water and methanol (2:8, *v*/*v*). The sample was then vortex mixed for 20–30 s, incubated for 10–15 min, vortex mixed again and centrifuged for 10 min at 13,400 rpm (Eppendorf MiniSpin). Supernatant (100 μL) was transferred into a vial and analyzed.

#### 3.3.2. Apparatus and LC-MS/MS Conditions

Analyses were carried out using a Shimadzu LC-20AD Prominence chromatograph (Shimadzu, Tokyo, Japan) equipped with a binary gradient pump, cooled autosampler SIL-20AC and column oven. A column packed with a reversed-phase sorbent ProntoSil 120-AQC18 (2 × 75 mm, 5 µm, EcoNova, Novosibirsk, Russia) was used for chromatographic separations. Mobile phase was water (eluent A) and MeOH (eluent B). The following gradient was used: 0 min—10% B; 1 min—90% B; 4.6 min—90%; 4.7 min—100% B; 6.0 min—100% B, followed by the equilibration of the column. Flow rate was 330 µL/min; injection volume was 10 µL. Mass spectrometric detection was performed on an ABSCIEX 6500 QTRAP mass spectrometer (AB SCIEX, Framingham, MA, USA) using negative electrospray ionization. The following parameters were set for the detection: scan mode—MRM, curtain gas (CUR) = 30 psi, collision-induced dissociation gas (CAD) = Medium, ion source voltage (IS) = 5500 V, gas drier temperature (TEM) = 250 °C, sprayer gas (GS1) = 15 psi, drier gas (GS2) = 20 psi, entrance potential (EP) = 10 V and dwell time = 80 msec. Detection parameters for agents **4c** and **5d** are shown in Table S1 (Supporting Information). The instruments were controlled, and the data were collected using Analyst 1.6.3 software (AB SCIEX); data processing was performed using MultiQuant 2.1 software (AB SCIEX).

### 3.4. Computations

Computations were calculated using Gaussian16 [49] with a M06-2X/6-311++G** level of theory [50]. Grimme’s dispersion correction (D3) was included [51]. Solvation was modeled using the solvation model based on density (SMD = H_2_O) [52].

## 4. Conclusions

The synthesis of novel sterically hindered phenols containing benzofuroxan fragments obtained via aromatic nucleophilic substitution reaction of 7-chloro-4,6-dinitrobenzofuroxan is presented. Depending on the initial ratio of reagents, it is possible to vary the composition of the final products, leading to the formation of compounds with a composition of 2:1 or compounds 1:1. Antimicrobial activity and antitumor potential were studied for the phenols/benzofuroxan hybrids. Most substances exhibit high cytotoxicity against human duodenal adenocarcinoma (HuTu 80), human breast adenocarcinoma (MCF-7) and human cervical carcinoma cell lines. The IC_50_ values of compounds **4c** and **5d** for these lines ranged from 0.9 to 5.9 µM and were either comparable to or exceeded the activity of Doxorubicin and Sorafenib. Moreover, the selectivity indices for healthy cells for the compound **5d** also exceed those for the reference drugs. A study of the mechanisms of cytotoxicity suggests that the latter can be associated with the induction of apoptosis along the internal mitochondrial pathway and an increase in ROS production. Encouragingly, all tested compounds do not show hemolytic activity (HC_50_ >100 µM). The biostability of the leading compounds was evaluated in the whole blood of mice, where the substances remained unchanged for two h. This is a positive sign for their future quantitative determination in biological matrices. When studying antimicrobial activity, we note an interesting trend that the effect only appears when at least two benzofuroxan moieties are introduced per phenol.

Overall, the combination of sterically hindered phenol and benzofuroxan in one molecule leads to a number of positive effects, including increased ROS production and greater cytotoxicity. Compounds **4c** and **5d** can be considered a promising basis for the development of antitumor drugs.

## Figures and Tables

**Table 1:** In vitro cytotoxic effects for the tested SHP-benzofuroxan hybrids.

Compounds	IC_50_, µM									
	Cancer Cell Lines								Normal Cell Lines	
	HuTu 80	PC3	PANC-1	MCF-7	M-HeLa	T98G	A549	A375	WI38	Chang Liver
**3**	>100	nd	>100	nd	94.1 ± 8.6	nd	nd	nd	nd	>100
**2a**	> 100	nd	nd	31.0 ± 2.6	16.1 ± 1.3	nd	nd	nd	nd	>100
**2b**	86.6 ± 6.8	nd	nd	34.2 ± 3.0	17.1 ± 1.3	nd	nd	nd	nd	>100
**2c**	>100	nd	nd	21.8 ± 1.7	38.0 ± 2.8	nd	nd	nd	nd	>100
**2d**	63.2 ± 5.6	nd	nd	16.0 ± 1.2	7.4 ± 0.7	nd	nd	nd	nd	52 ± 3.5
**2e**	nd	nd	nd	>100	45.3 ± 3.2	nd	nd	nd	nd	>100
**4a**	5.2 ± 0.4	14.0 ± 1.2	20.0 ± 1.7	7.6 ± 0.6	4.2 ± 0.3	12.4 ± 1.2	13.5 ± 1.2	13.5 ± 1.2	7.2 ± 0.6	12.0 ± 1.2
**4b**	5.4 ± 0.3	11.0 ± 0.8	5.3 ± 0.4	3.1 ± 0.2	2.9 ± 0.2	8.3 ± 0.7	9.3 ± 0.8	11.4 ± 0.9	5.3 ± 0.4	7.0 ± 1.5
**4c**	0.9 ± 0.07	1.2 ± 1.0	11.2 ± 0.9	1.1 ± 0.1	0.9 ± 0.07	2.2 ± 0.1	0.9 ± 0.08	2.9 ± 0.2	2.0 ± 0.1	2.1 ± 0.7
**4d**	13.1 ± 1.1	11.3 ± 0.9	11.9 ± 0.9	9.0 ± 0.7	6.3 ± 0.5	7.9 ± 0.7	9.0 ± 0.7	17.0 ± 1.4	11.3 ± 1.1	21.0 ± 2.4
**4e**	17.1 ± 1.3	12.3 ± 1.1	17.4 ± 1.3	21.5 ± 1.8	12.0 ± 1.0	40.2 ± 3.1	48.3 ± 3.7	53.1 ± 4.1	25.7 ± 2.2	14.3 ± 1.3
**5a**	11.0 ± 0.9	7.7 ± 0.6	13.5 ± 1.1	12.1 ± 1.1	7.7 ± 0.6	26.8 ± 2.2	48.4 ± 3.5	21.6 ± 1.8	11.4 ± 0.9	15.1 ± 1.7
**5c**	3.9 ± 0.3	6.8 ± 0.5	5.0 ± 0.3	4.5 ± 0.4	2.4 ± 0.2	7.7 ± 0.6	7.7 ± 0.6	9.0 ± 0.8	5.0 ± 0.4	4.1 ± 0.8
**5d**	4.9 ± 0.3	5.9 ± 0.4	2.8 ± 0.2 (SI = 3.3)	2.1 ± 0.1 (SI = 4.4)	2.0 ± 0.1 (SI = 4.6)	4.8 ± 0.4	5.2 ± 0.4	5.9 ± 0.5	8.4 ± 0.7	9.2 ± 1.9
DOX	0.2 ± 0.01	1.4 ± 0.1	2.2 ± 0.1	0.4 ± 0.03	2.1 ± 0.2	2.0 ± 0.1	0.7 ± 0.05	0.3 ± 0.02	0.4 ± 0.02	0.5 ± 0.04
SF	6.2 ± 0.5	11.3 ± 0.9	12.0 ± 1.1	27.5 ± 2.3	25.0 ± 1.9	8.6 ± 0.7	25.2 ± 2.2	6.8 ± 0.5	6.6 ± 0.5	21.7 ± 1.7

HuTu-80 is a duodenal adenocarcinoma; PC3 is a human prostate adenocarcinoma; PANC-1 is a human pancreatic cancer cell line; MCF-7 is a human breast adenocarcinoma (pleural fluid); M-HeLa is a human cervix epitheloid carcinoma; T98G is a glioblastoma cell line; A549 is an adenocarcinomic human alveolar basal epithelial cell line; A375 is human amelanotic melanoma cell line; WI 38 is an human embryonic lung; Chang liver is a line of human liver cells; DOX—doxorubicin, SF—sorafenib. The experiments were repeated three times. SI—selectivity index values relative to Chang liver; nd—not determined. Cells were incubated with substances for 48 h.

**Table 2:** Antimicrobial activity of studied compounds.

Compounds	Minimum Inhibitory Concentration (MIC), µg/mL				
	*Sa*	*Bc*	*Ef*	*MRSA-1*	*MRSA-2*
**2a**	*-*	*-*	*-*	*-*	*-*
**2c**	*-*	*-*	*-*	*-*	*-*
**2d**	*-*	*-*	*-*	*-*	*-*
**3**	*-*	*-*	*-*	*-*	*-*
**4a**	*-*	*-*	*-*	*-*	*-*
**4c**	*-*	*-*	*-*	*-*	*-*
**4d**	*-*	*-*	*-*	*-*	*-*
**5a**	125 ± 11	62.5 ± 5.3	125 ± 10	250 ± 20	250 ± 20
**5c**	31.3 ± 2.3	31.3 ± 2.5	62.5 ± 5.3	62.5 ± 5.5	250 ± 20
**5d**	15.6 ± 1.2	62.5 ± 5.4	62.5 ± 5.2	62.5 ± 5.3	250 ± 19
Chloramphenicol	31.3 ± 2.2	62.5 ± 5.4	62.5 ± 5.2	nd	nd
Ketoconazole	-	-	-	-	-
	Minimum bactericidal and fungicidal concentrations (MBC), (MFC) µg/ml				
**5a**	250 ± 19	-	-	250 ± 21	-
**5c**	-	250 ± 20	-	-	-
**5d**	125 ± 10	-	-	250 ± 20	250 ± 19
Chloramphenicol	-	-	-	-	-
Ketoconazole	-	-	-	-	-

Average of three values measured; ± standard deviation (SD);—means non-active; nd—not determined.

## Data Availability

Data is contained within the article and supplementary material.

## Supplementary Materials

The following supporting information can be downloaded at: https://www.mdpi.com/article/10.3390/ph16040499/s1, Figures S1–S45 (p. 2–46)—copies of NMR spectra of all synthesized compounds; Figure S46 Dependence of substances 4c and 5d peak area on the chromatograms of the initial value (5 min); Table S1 (p. 47–48)—biostability studies; p. 49–51—computational data.

## Sample Availability

Samples of all described compounds are available from the author E. Chugunova.
