# Supplementary material for: Diverse Biological Activity of Benzofuroxan/Sterically Hindered Phenols Hybrids
Source: Pharmaceuticals (Basel). 2023 Mar 28;16(4):499. doi: 10.3390/ph16040499 (PMC10145285; doi:10.3390/ph16040499)
Supplement: Supplementary file 1 [file pharmaceuticals-16-00499-s001.zip › pharmaceuticals-2268981-supplementary.pdf]

SUPPLEMENTARY MATERIALS

Diverse biological activity of benzofuroxan/sterically hindered phenols hybrids

Elena Chugunova [1,*], Elmira Gibadullina [1], Kirill Matylitsky [1], Baurat Bazarbayev [2], Margarita Neganova [1], Konstantin Volcho [3], Artem Rogachev [3,4], Nurgali Akylbekov [5], Nguyen Hoang Bao Tran [2], Alexandra Voloshina [1], Anna Lyubina [1], Syumbelya Gumerova [1], Victor Syakaev [1], Alexander Burilov [1], Nurbol Appazov [5,6], Mukhtar Zhanakov [7], Leah Kuhn [8], Oleg Sinyashin [1] and Igor Alabugin [1,8]

[1] Arbuzov Institute of Organic and Physical Chemistry, FRC Kazan Scientific Center, Russian Academy of Sciences, Akad. Arbuzov st. 8, Kazan, 420088, Russia

[2] The Kazan National Research Technological University, Karl Marx st., 68, Kazan, 420015, Russia

[3] Department of Medicinal Chemistry, Novosibirsk Institute of Organic Chemistry, Lavrentiev av. 9, Novosibirsk 630090, Russia

[4] Novosibirsk State University, Pirogov St. 2, Novosibirsk 630090, Russia

[5] Laboratory of Engineering Profile "Physical and Chemical Methods of Analysis", Korkyt Ata Kyzylorda University, Aitekebie str. 29A, Kyzylorda 120014, Kazakhstan

[6] I. Zhakhaev Kazakh Scientific Research Institute of Rice Growing, Abay av. 25B, Kyzylorda 120008, Kazakhstan

[7] L.N. Gumilyov Eurasian National University, Satpayev str. 2, Astana, Kazakhstan

[8] Florida State University, Department of Chemistry and Biochemistry, 95 Chieftan Way, Tallahassee, Fl, 32306-3290, USA

* Correspondence: chugunova.e.a@gmail.com (EC); Tel.: +7-843-272-7324; neganova83@mail.ru (MN); Tel.: +7-843-272-7324.

Contents

Copies of NMR spectra 2-46

Biostability studies..47-48

Computational data..49-51

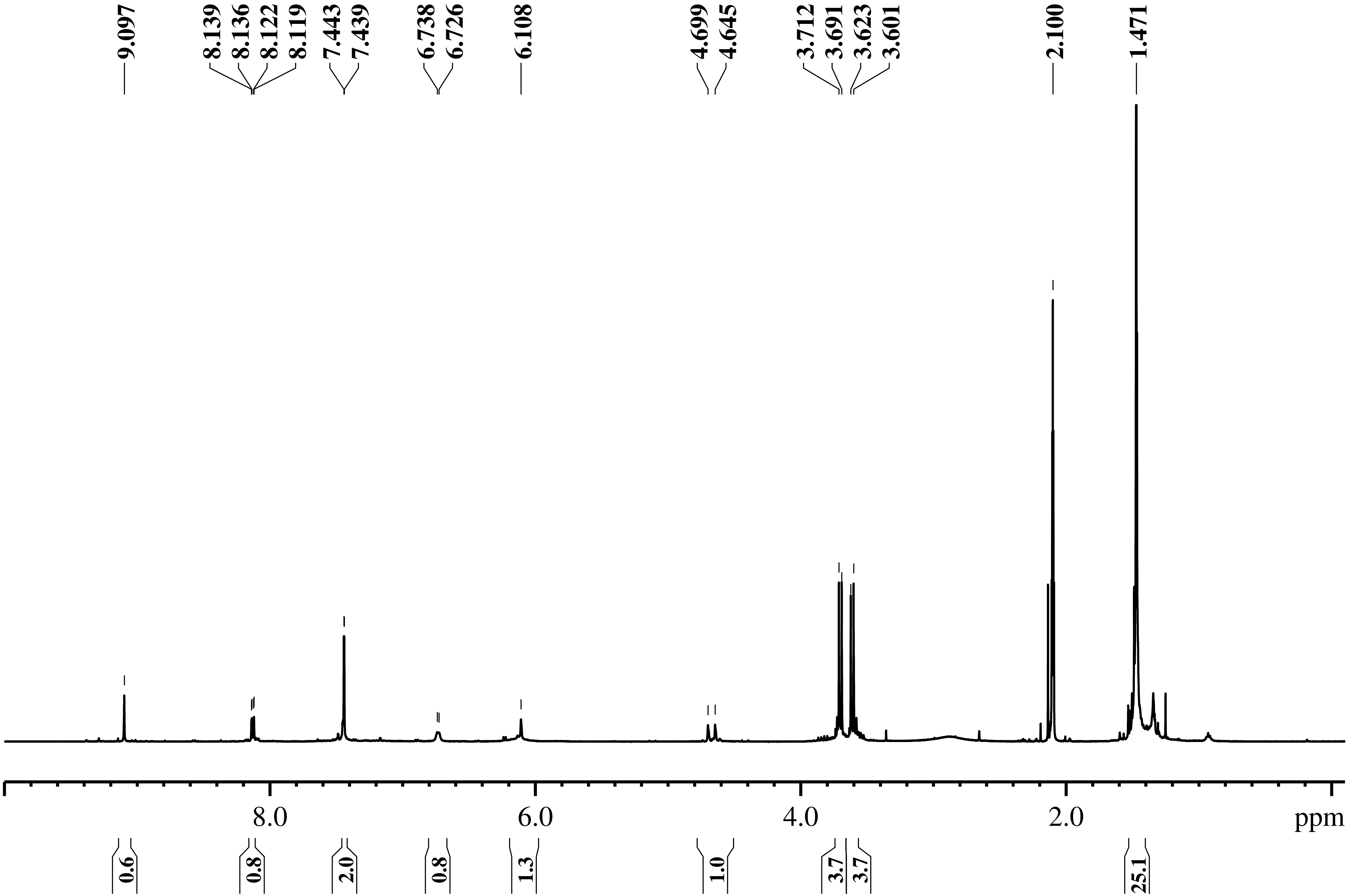

Figure S1. ^{1}H NMR (acetone-d_6, 500 MHz, 303 K) of compound **4a**

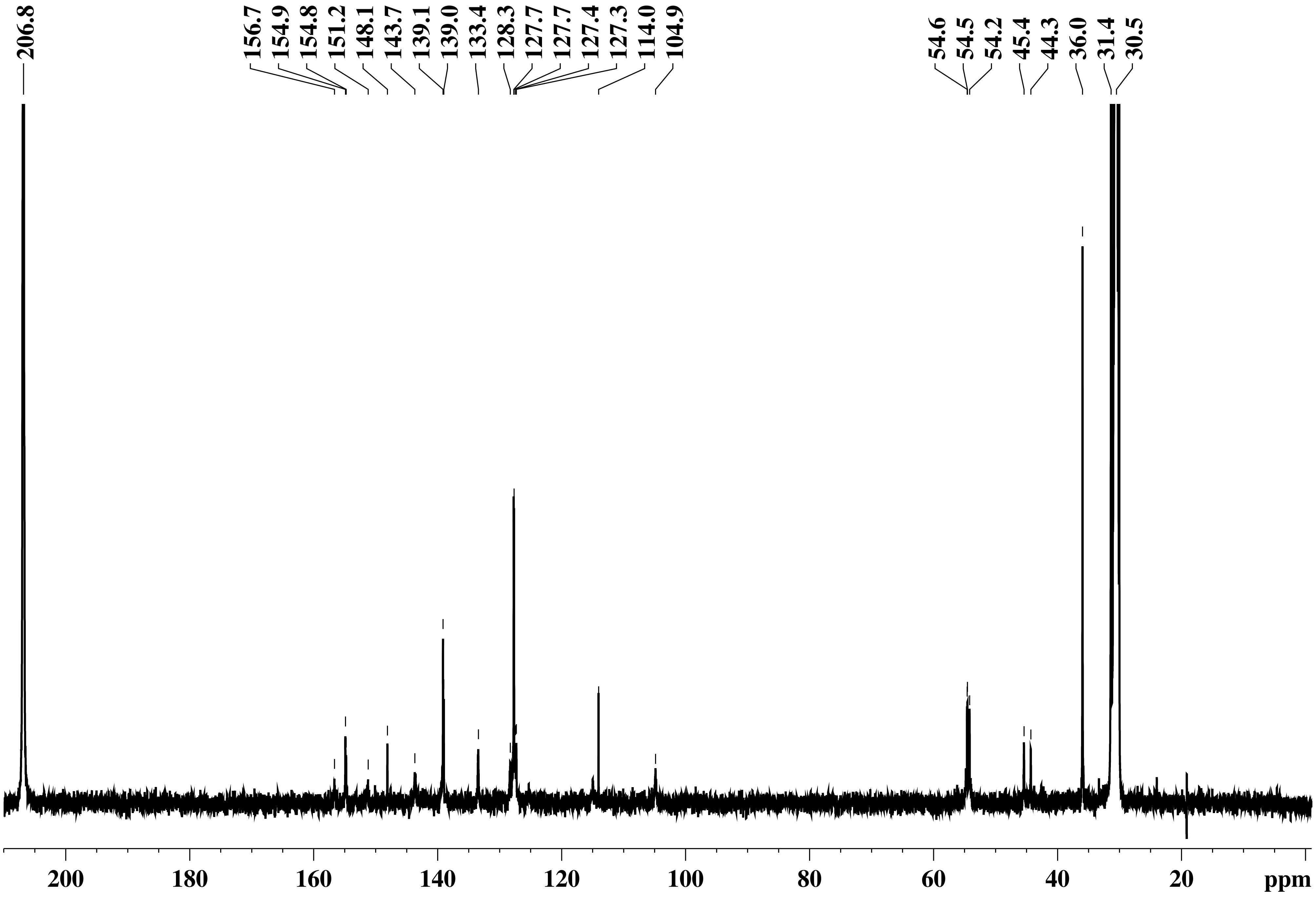

Figure S2. ^{13}C NMR (acetone-d_6, 126 MHz, 303 K) of compound **4a**

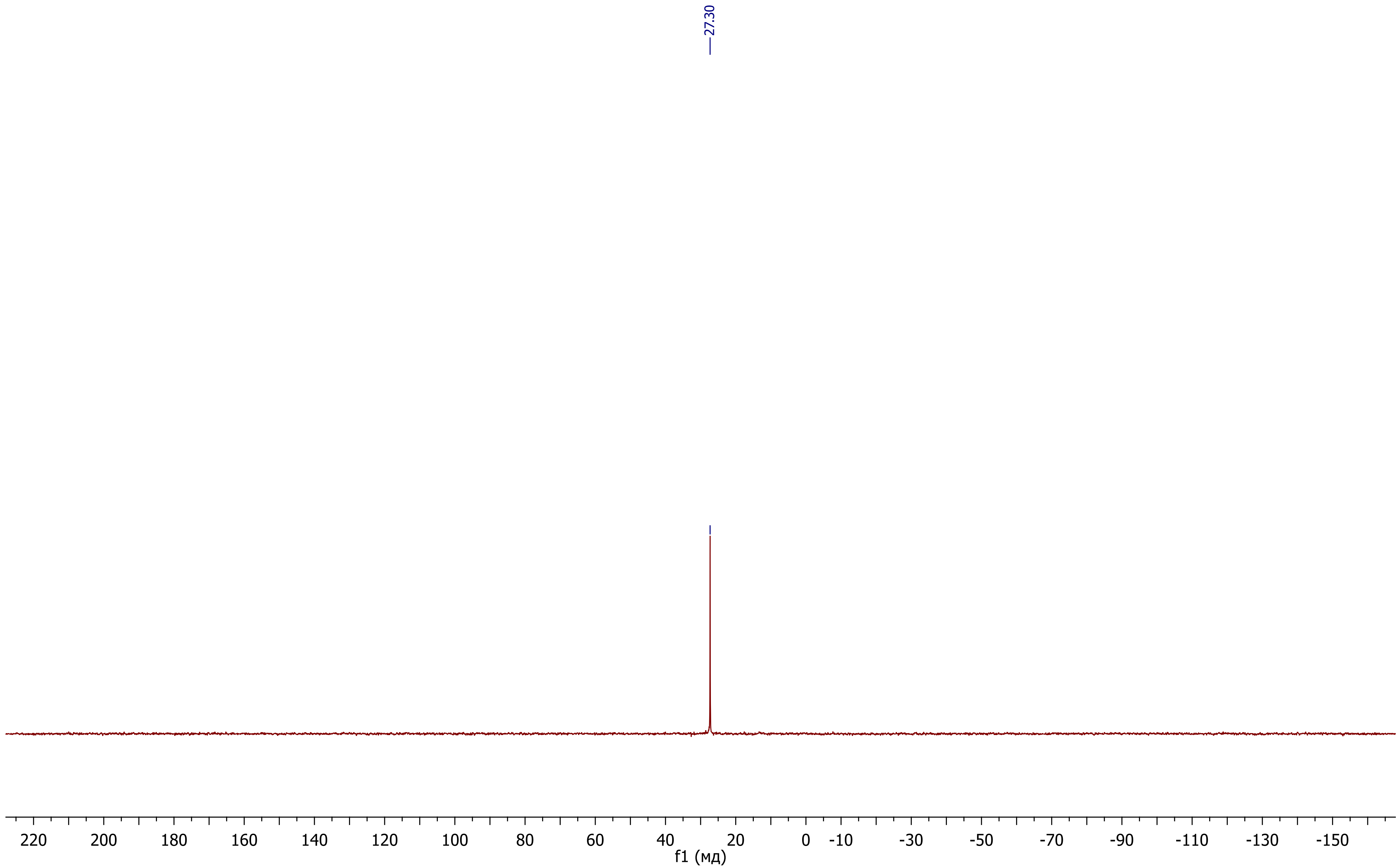

Figure S3. ^{31}P NMR (acetone-d_6, 162 Hz, 303 K) of compound **4a**

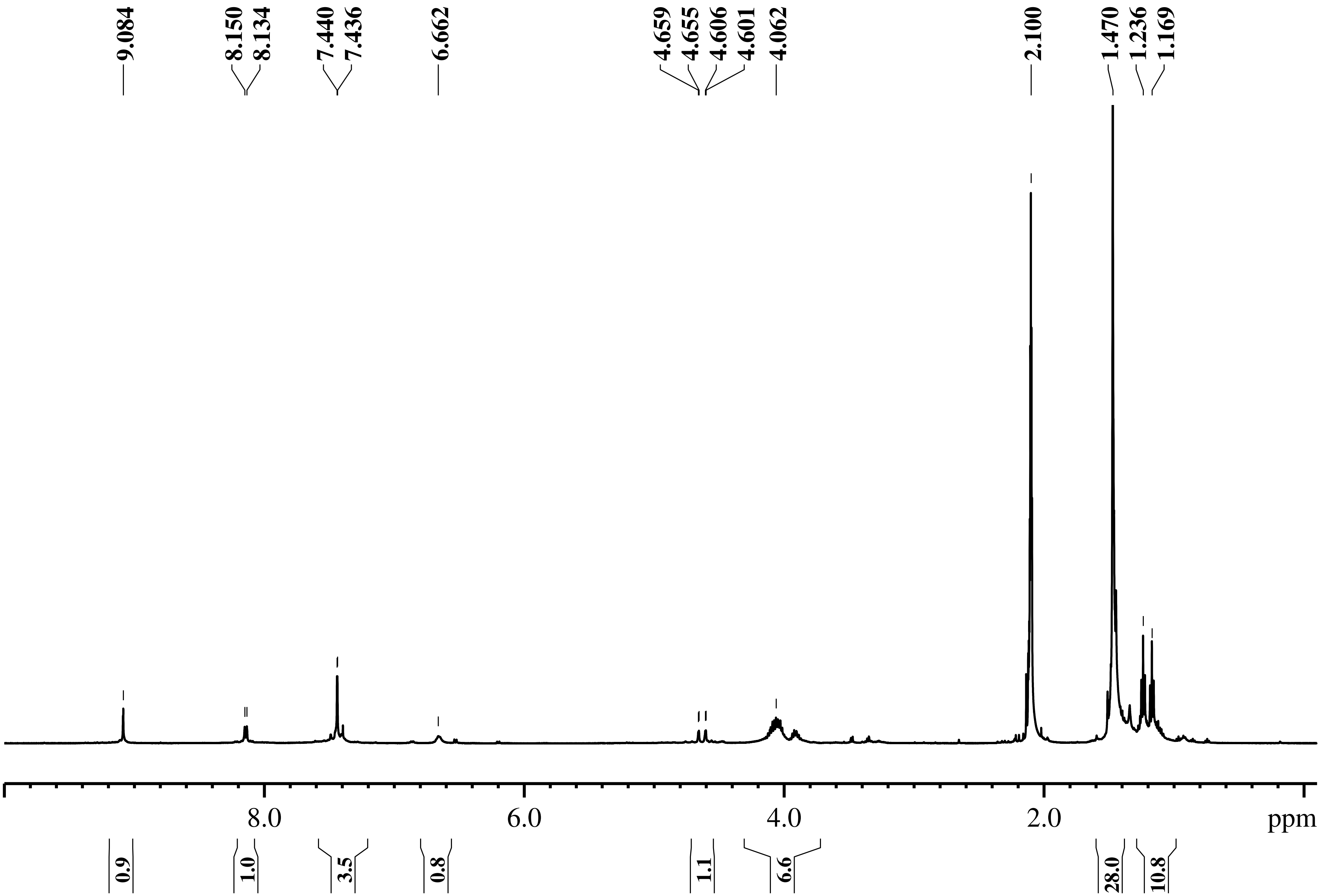

Figure S4. ^{1}H NMR (acetone-d_6, 500 MHz, 303 K) of compound **4b**

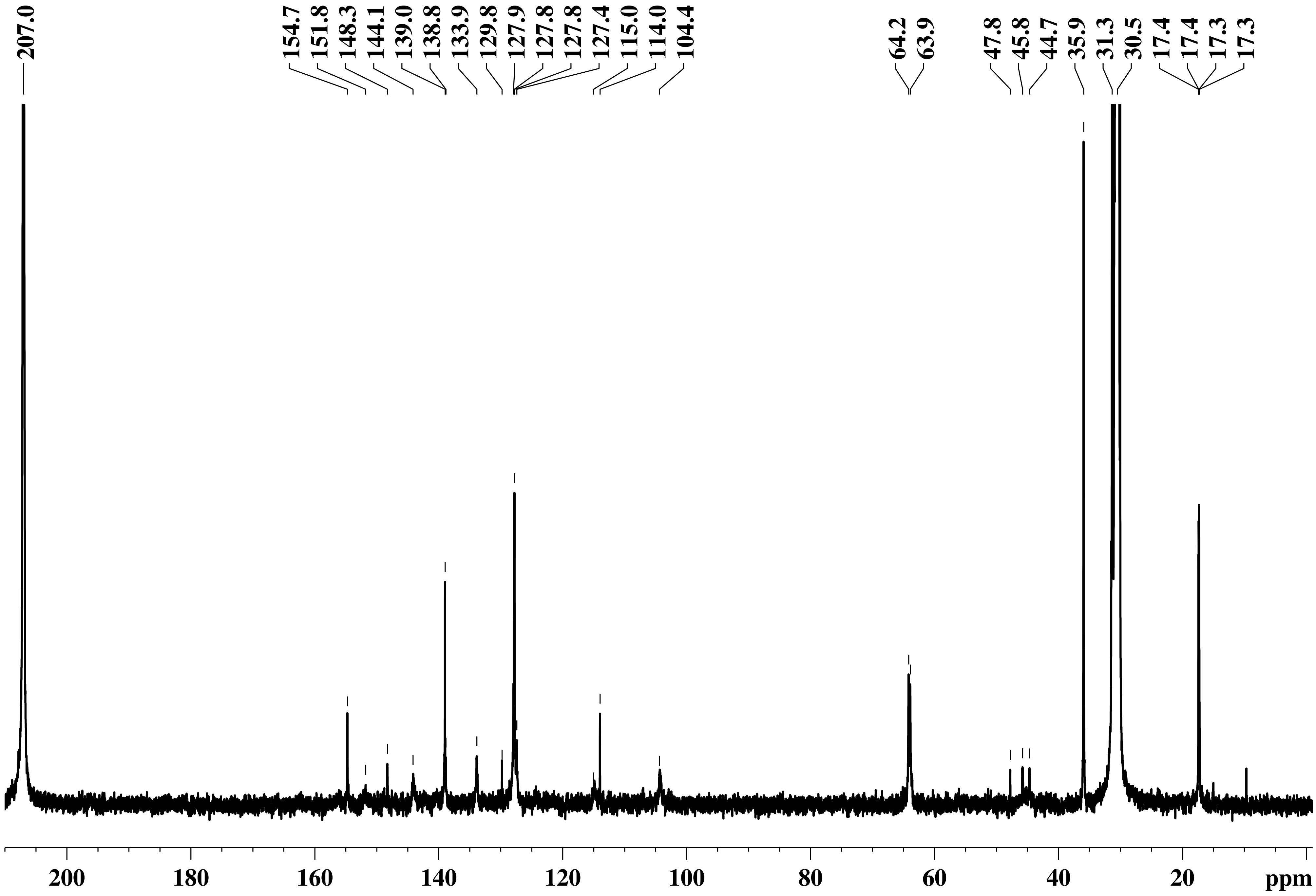

Figure S5. ^{13}C NMR (acetone-d_6, 126 MHz, 303 K) of compound **4b**

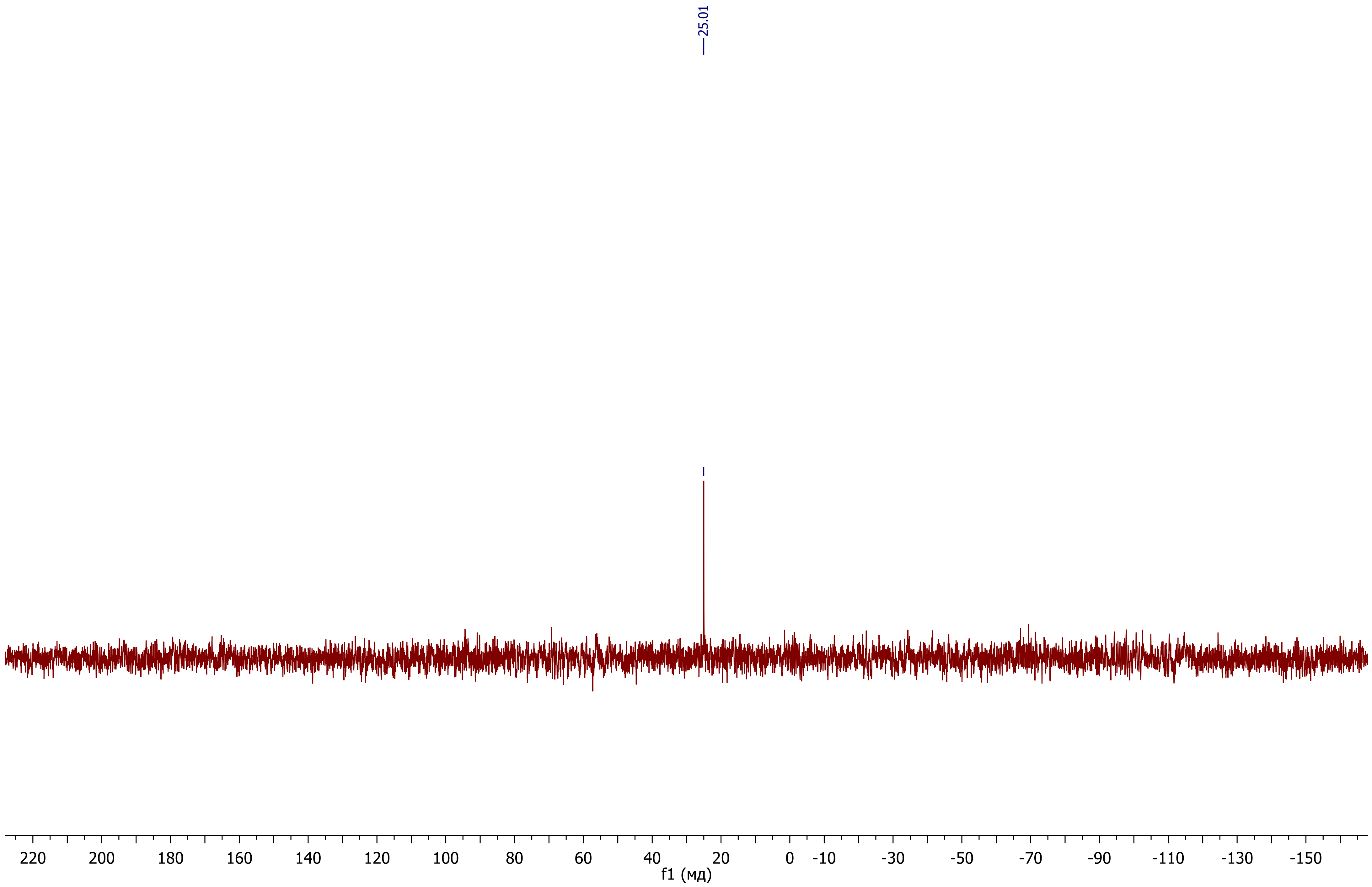

Figure S6. ^{31}P NMR (acetone-d_6, 162 Hz, 303 K) of compound **4b**

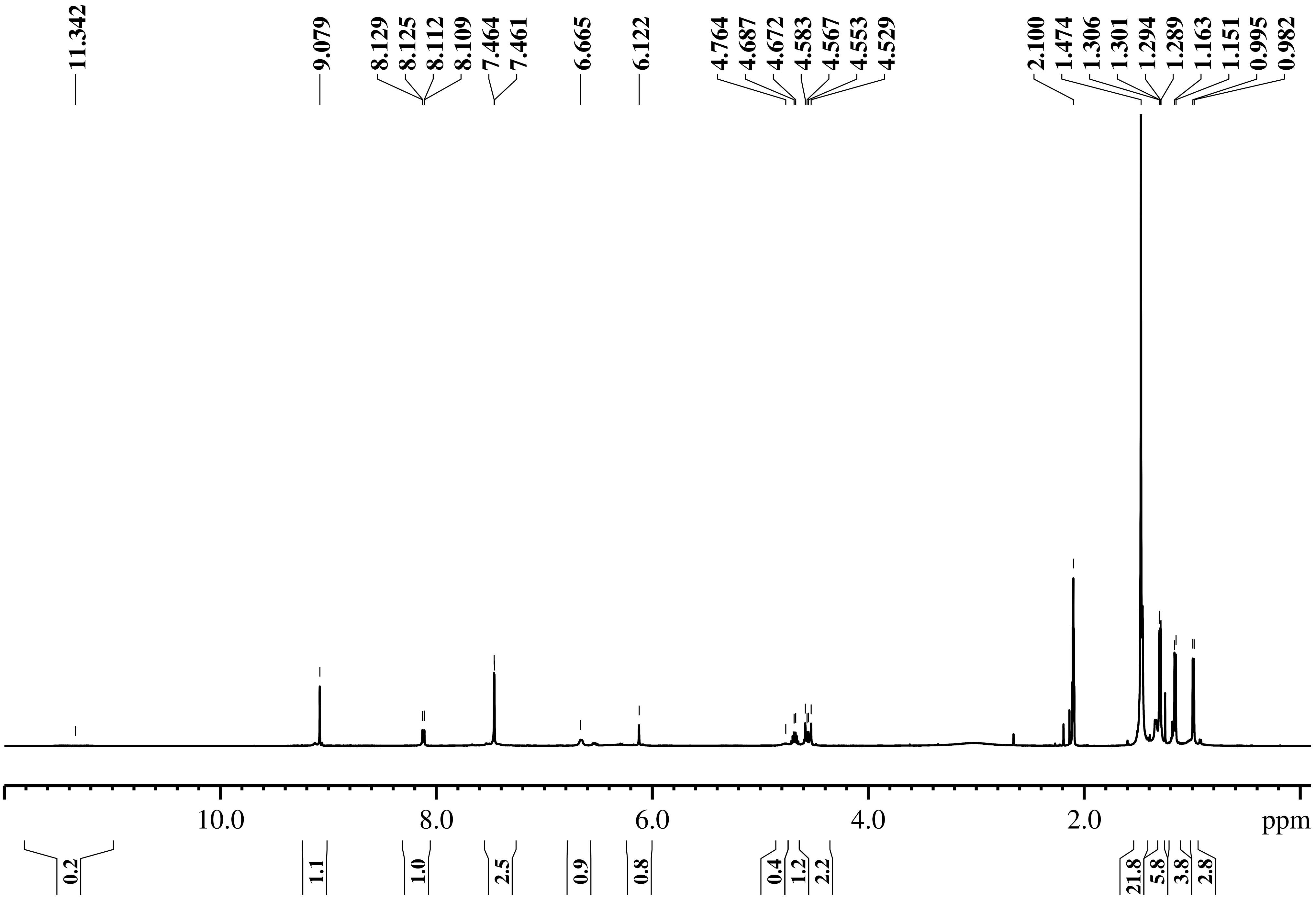

Figure S7. ^{1}H NMR (acetone-d_6, 500 MHz, 303 K) of compound **4c**

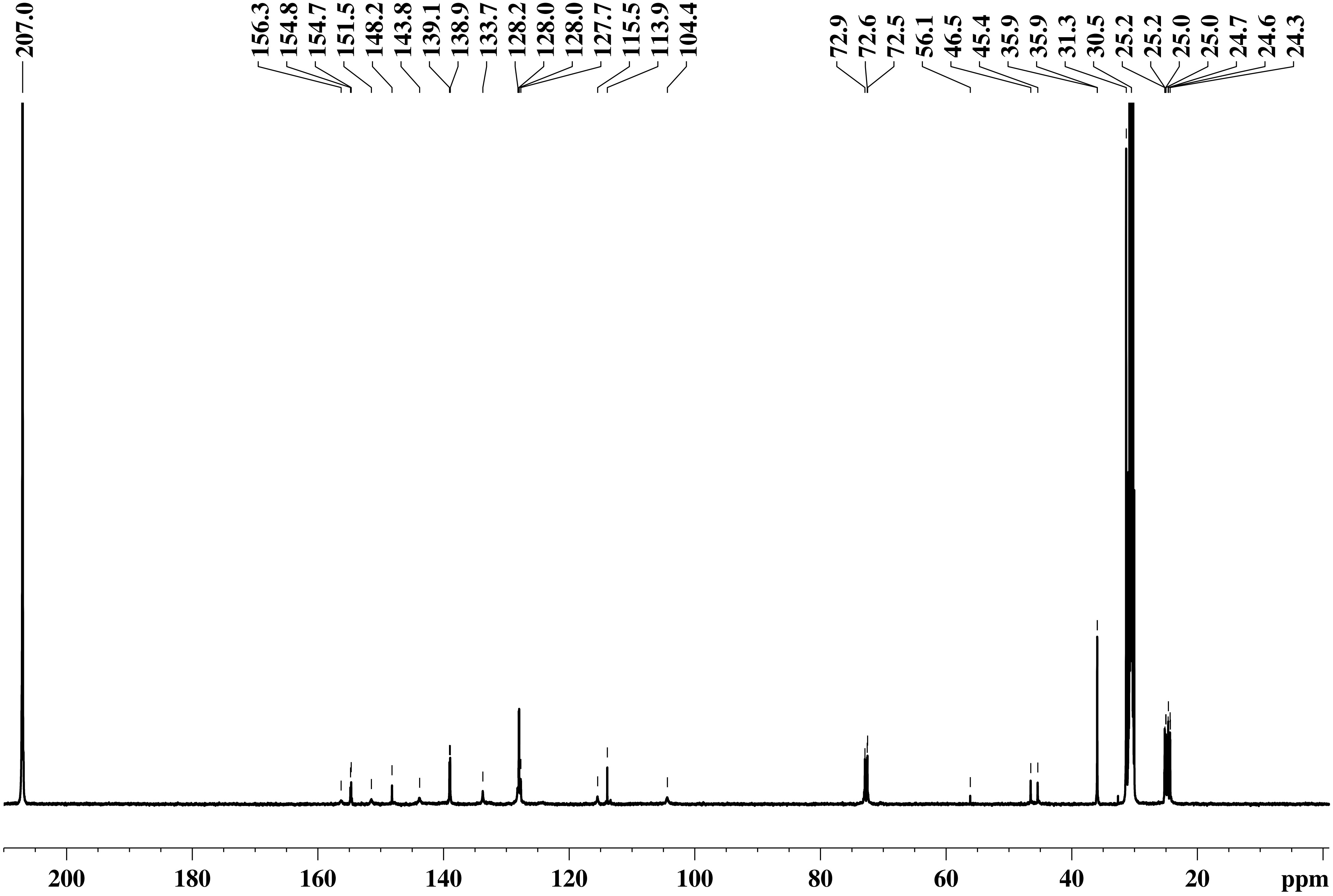

Figure S8. ^{13}C NMR (acetone-d_6, 126 MHz, 303 K) of compound **4c**

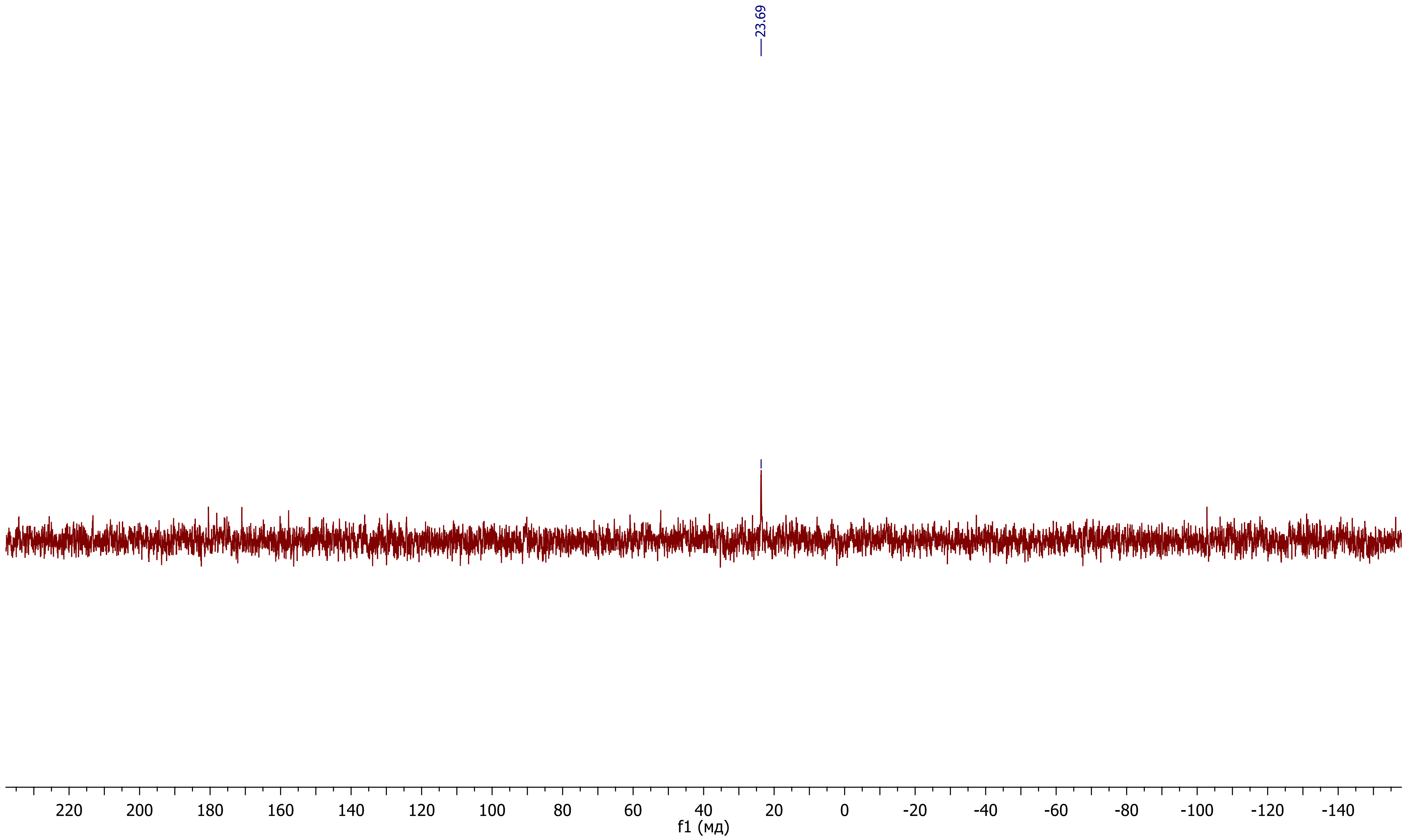

Figure S9. ^{31}P NMR (acetone-d_6, 162 Hz, 303 K) of compound **4c**

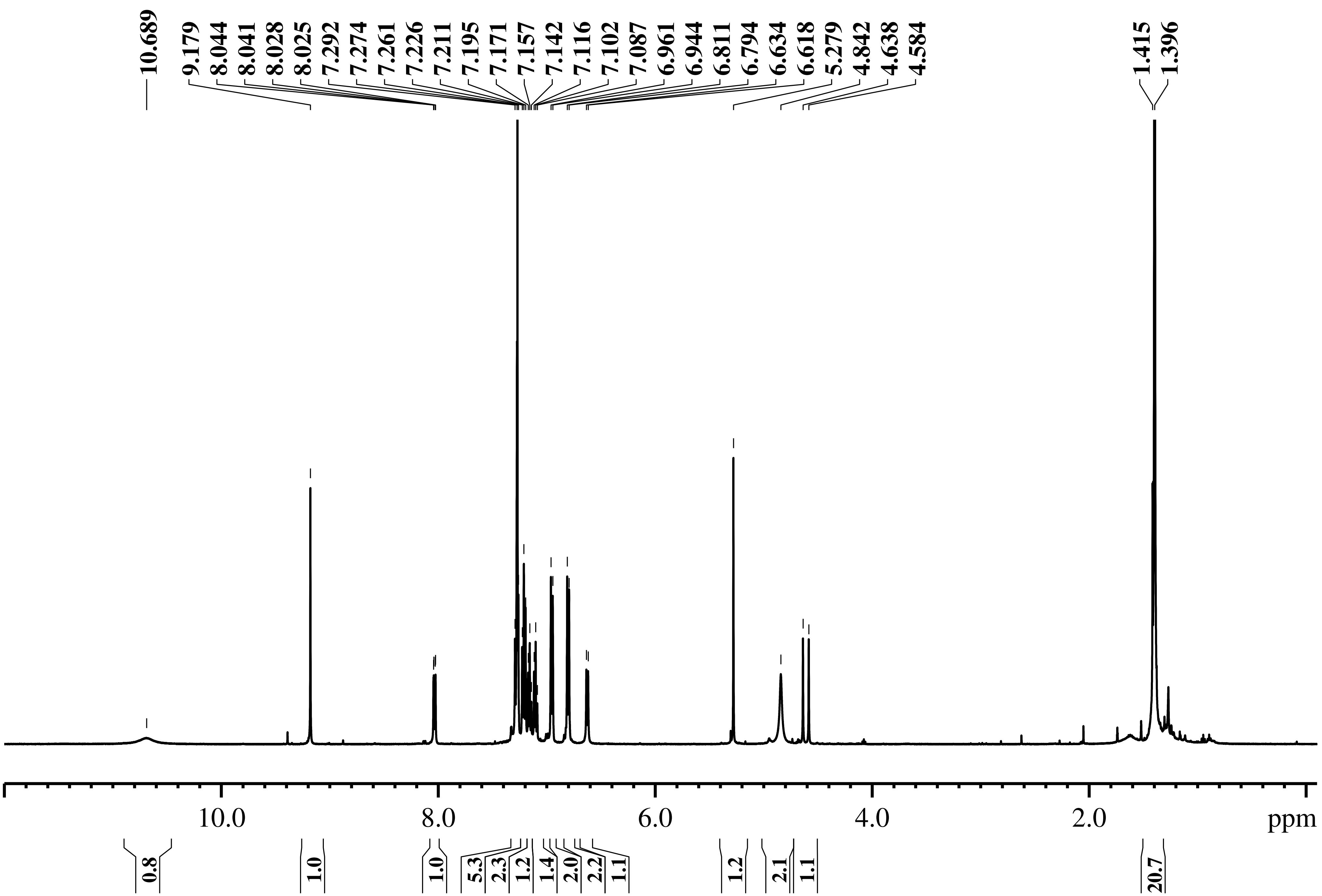

Figure S10. ^{1}H NMR ($CDCl_3$, 500 MHz, 303 K) of compound **4d**

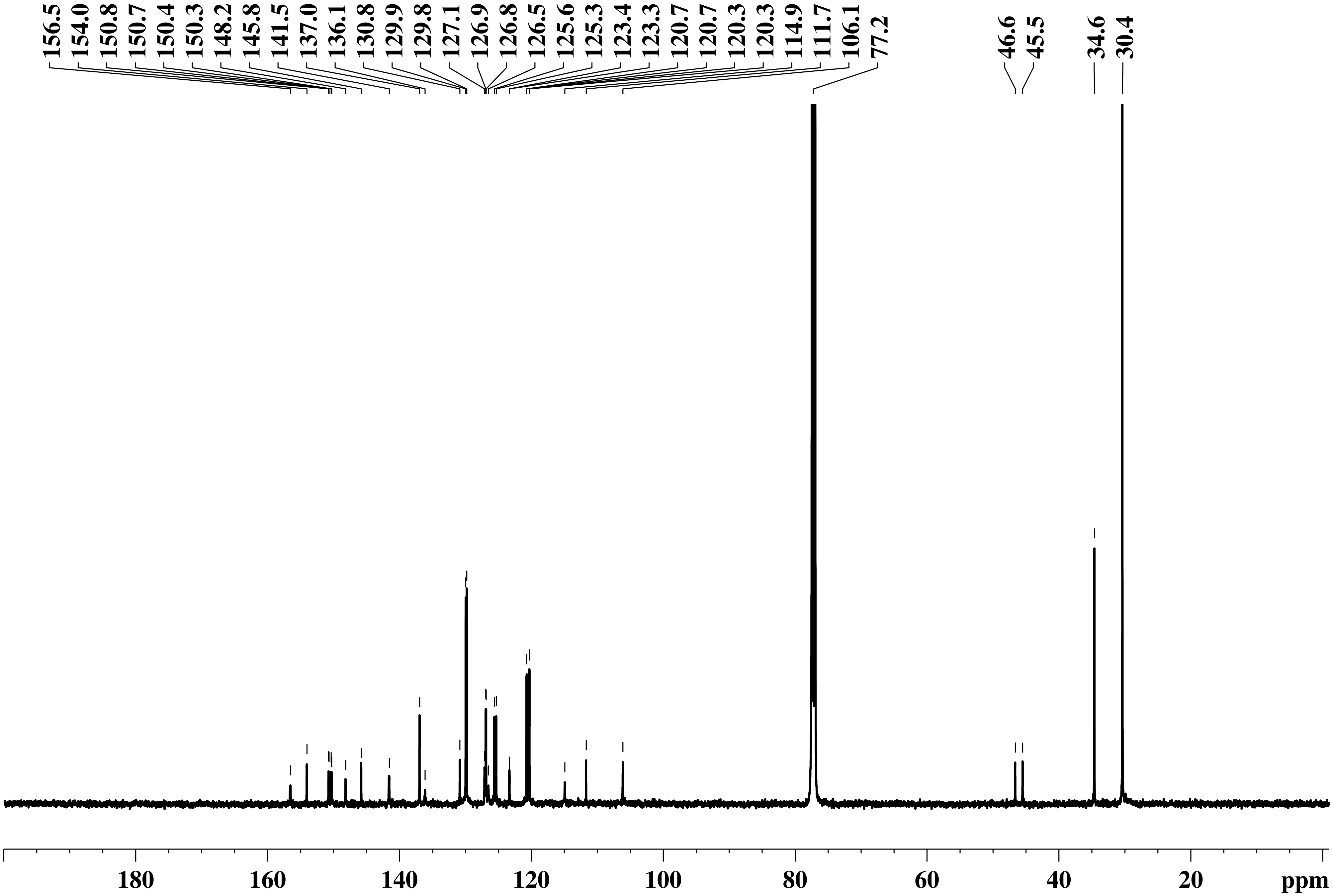

Figure S11. ^{13}C NMR ($CDCl_3$, 126 MHz, 303 K) of compound **4d**

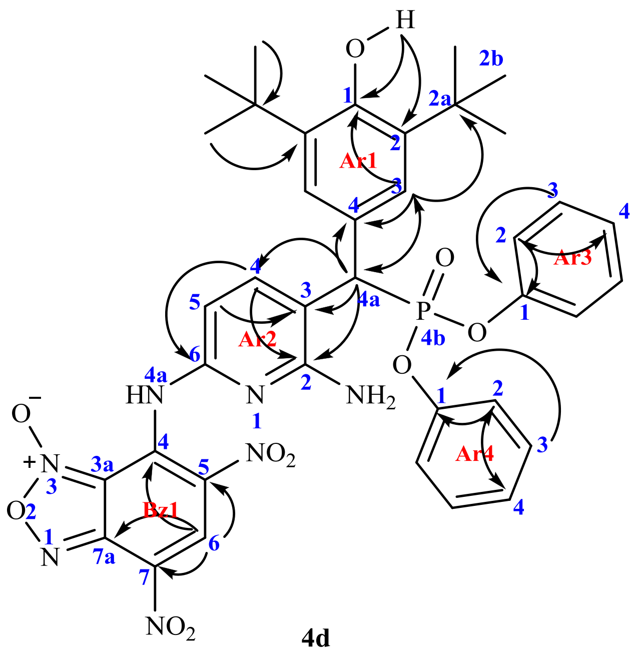

4d

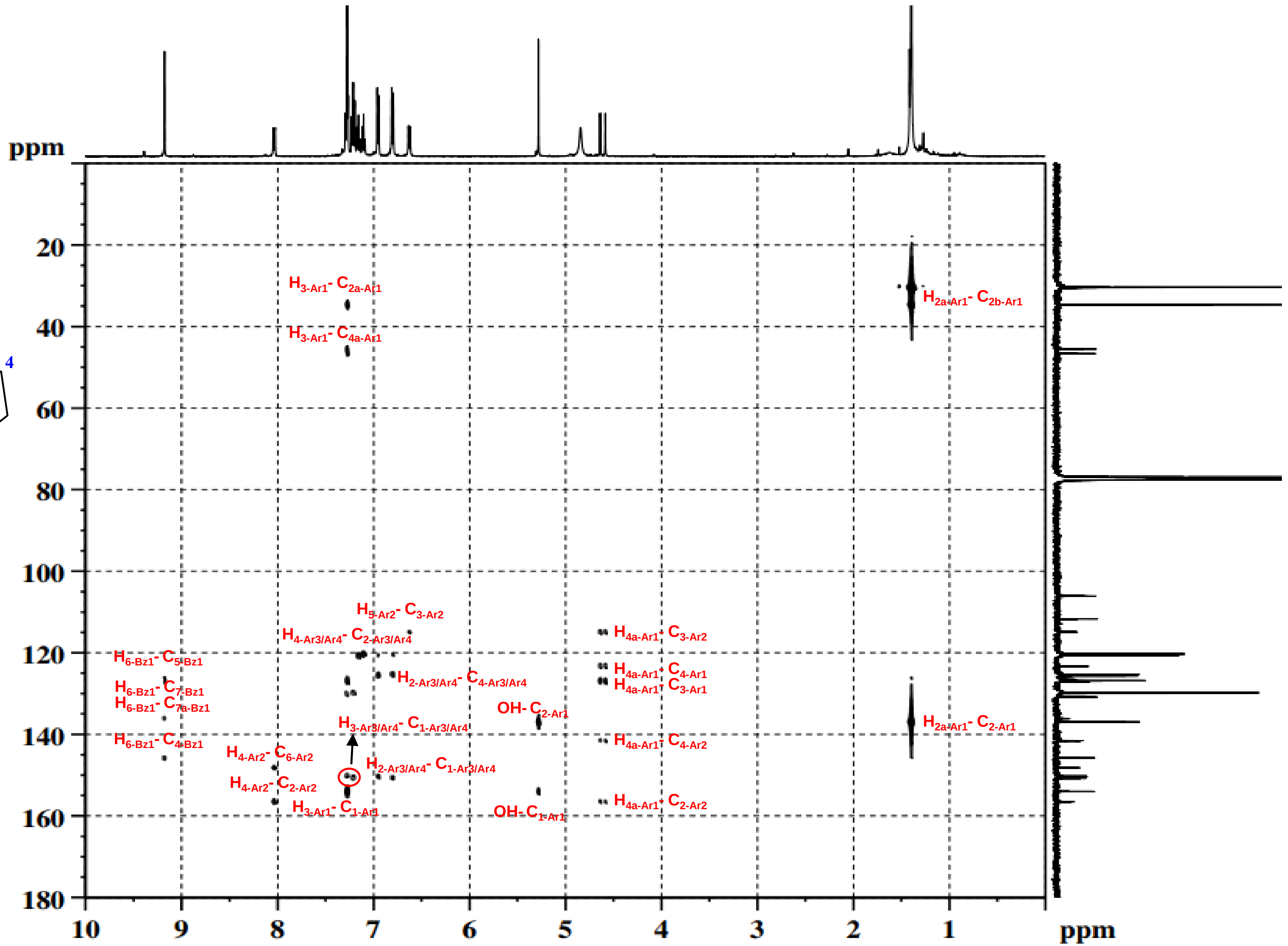

Figure S12. ^{1}H-^{13}C HMBC($CDCl_3$, 126 MHz, 303 K) of compound **4d**

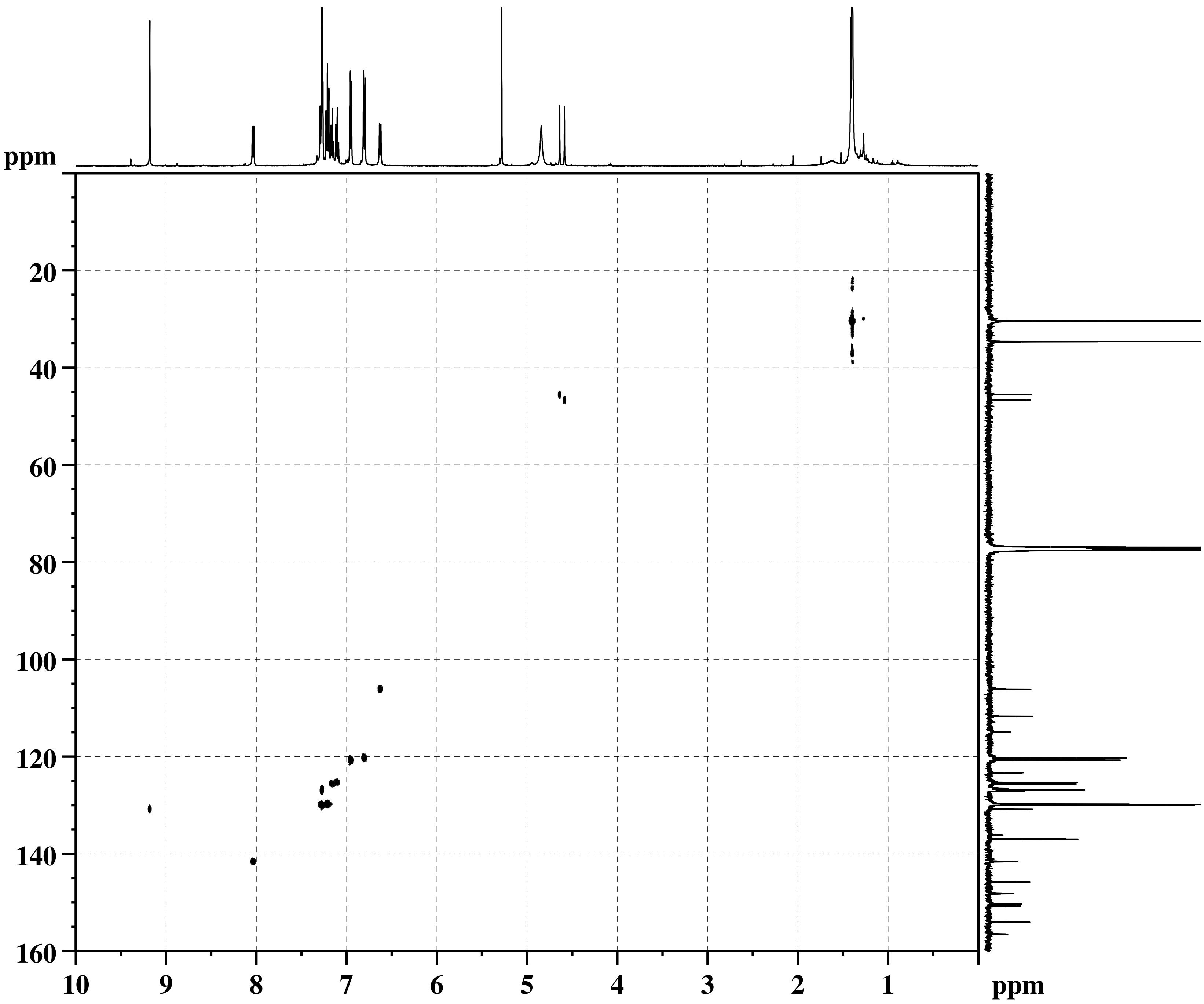

Figure S13. ^{1}H-^{13}C HSQC (CDCl$_3$, 126 MHz, 303 K) of compound **4d**

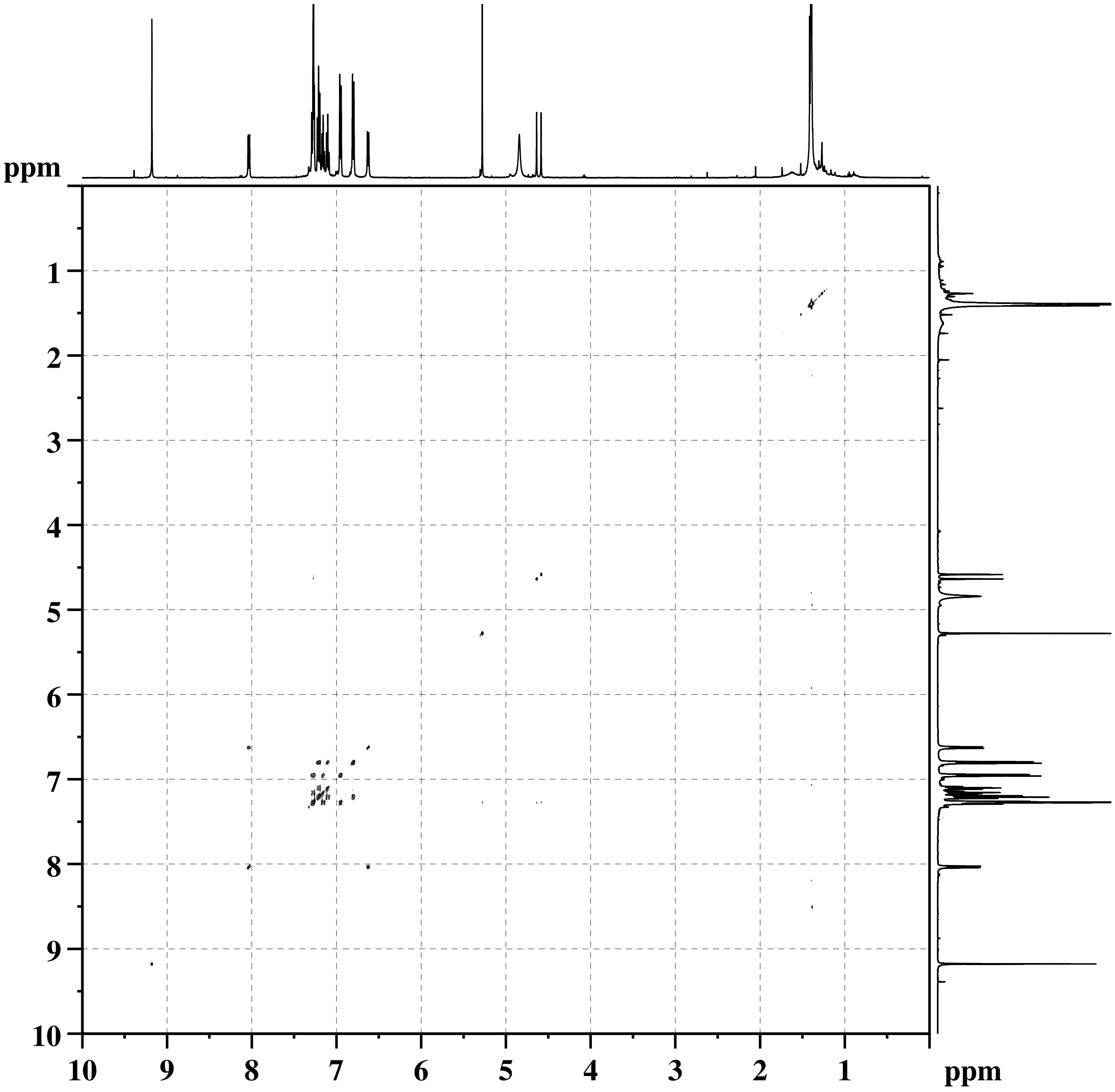

Figure S14. COSY (CDCl$_3$, 126 MHz, 303 K) of compound **4d**

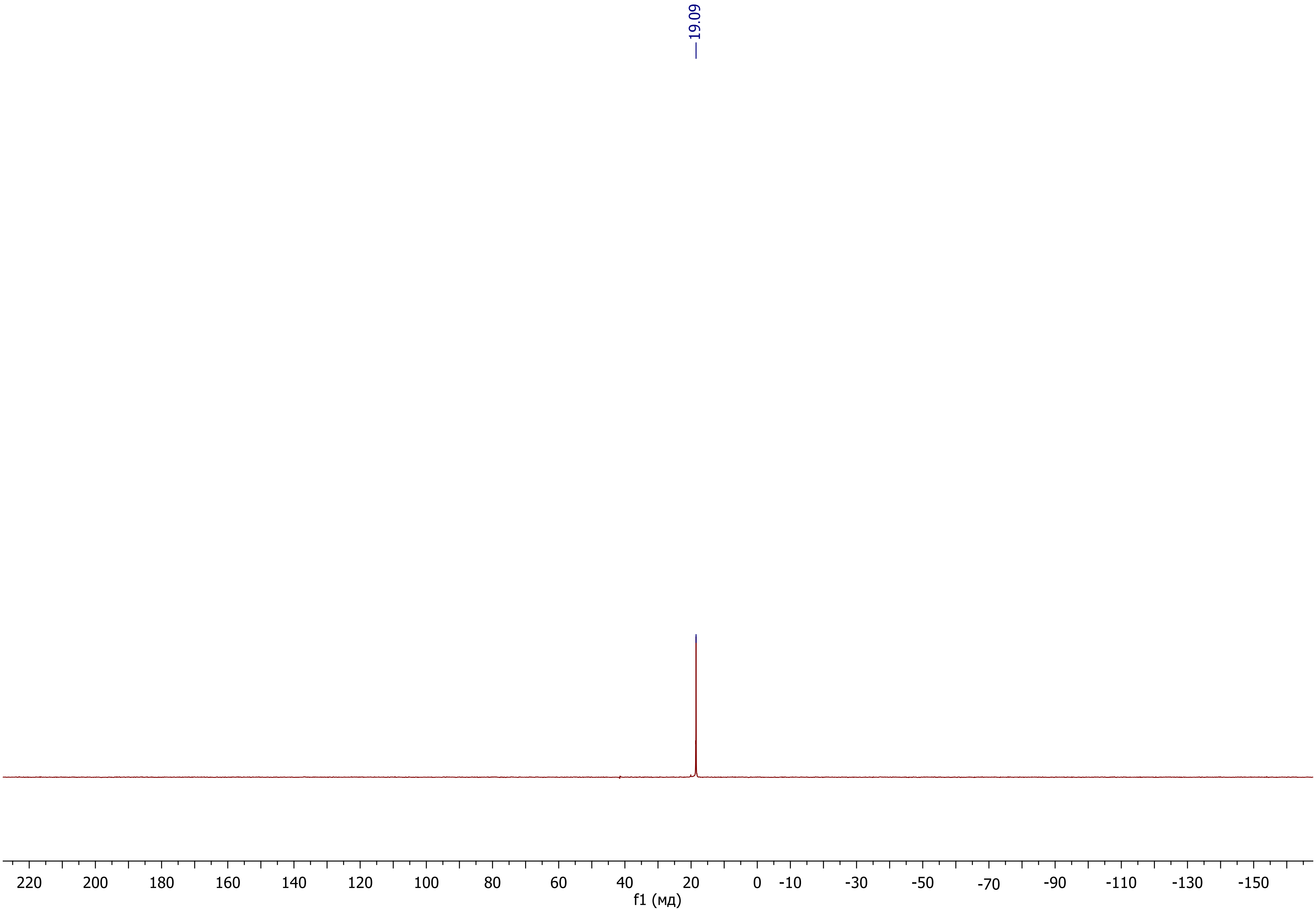

Figure S15. ^{31}P NMR (acetone-d_6, 162 Hz, 303 K) of compound **4d**

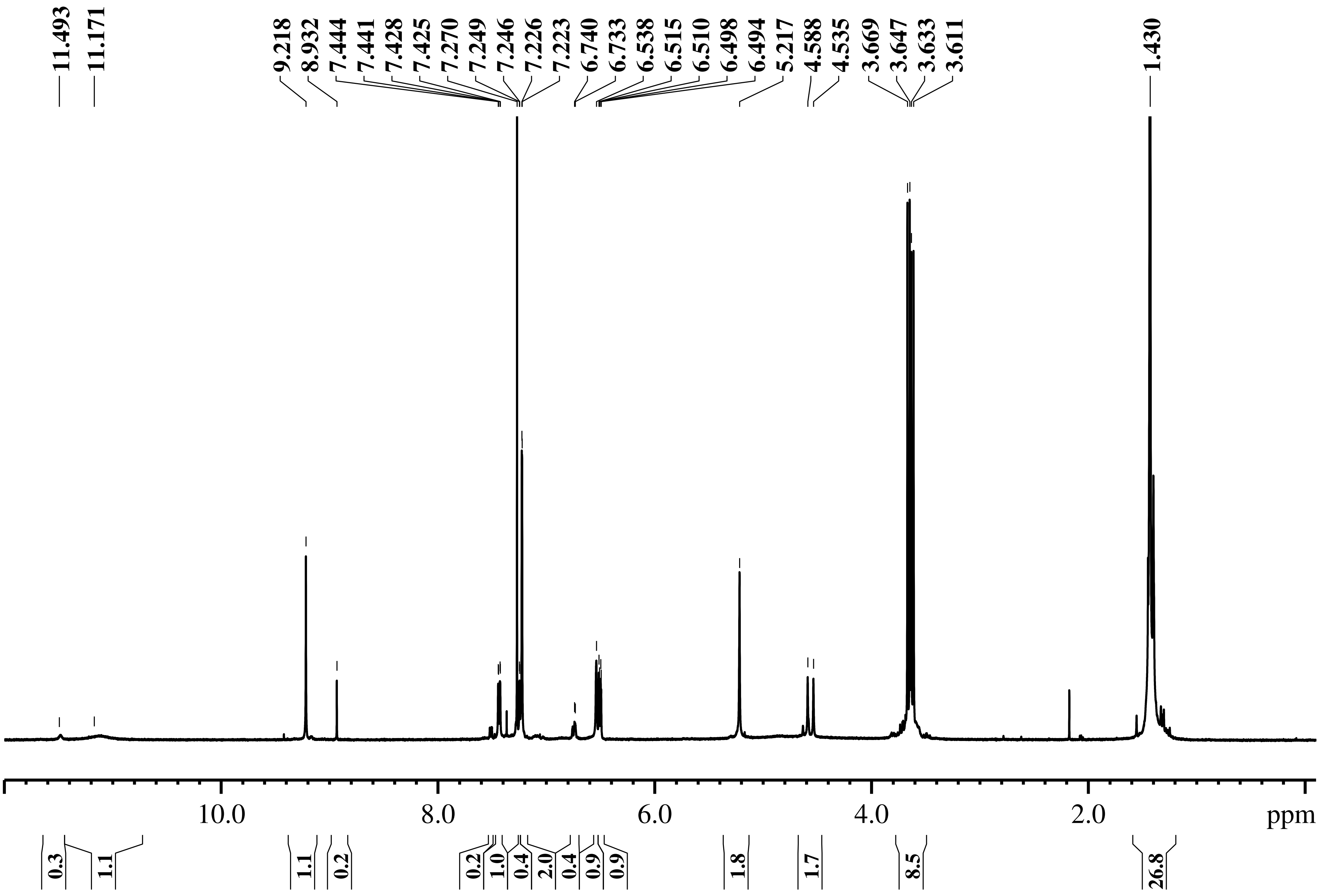

Figure S16. ^{1}H NMR ($CDCl_3$, 500 MHz, 303 K) of compound **4e**

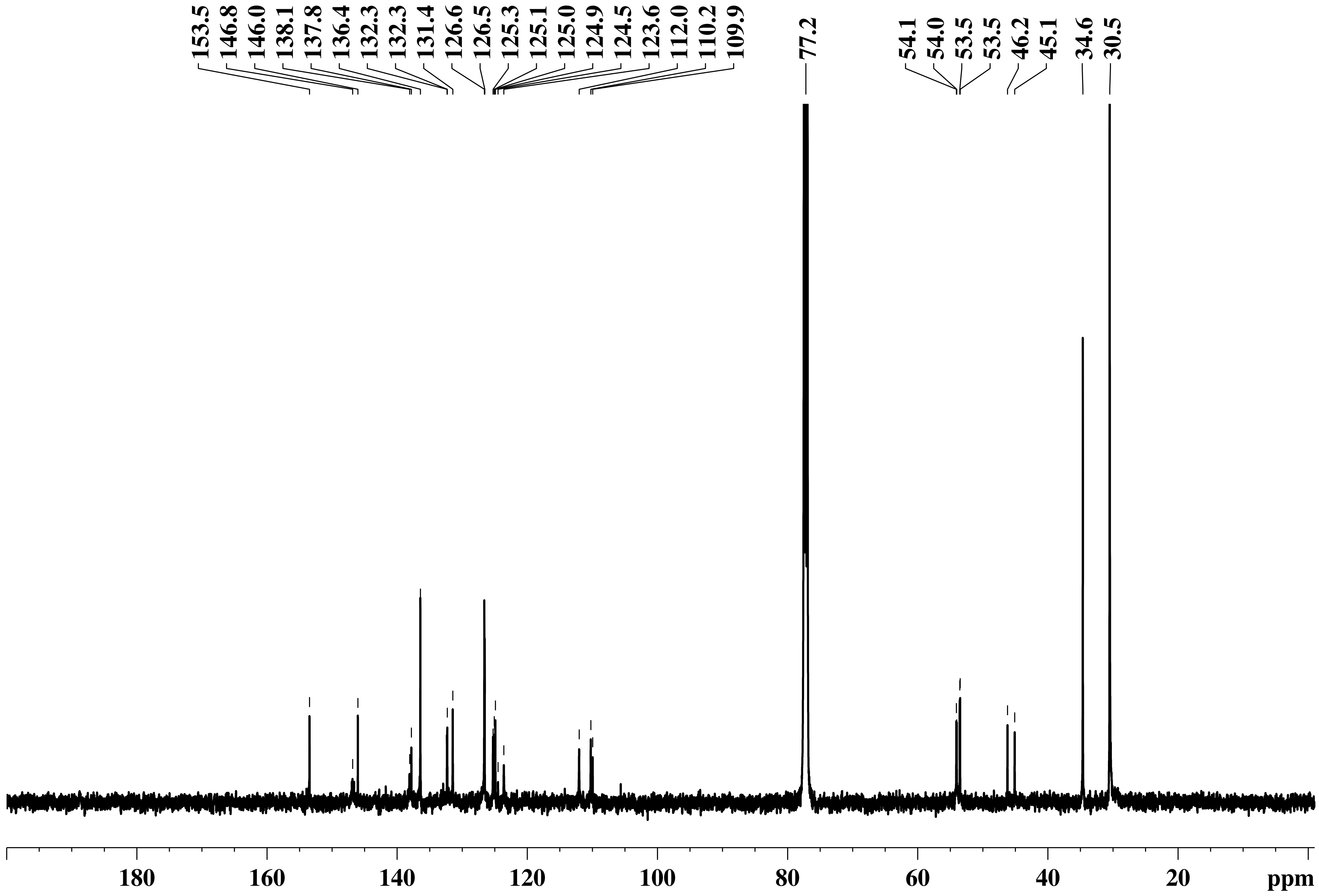

Figure S17. ^{13}C NMR ($CDCl_3$, 126 MHz, 303 K) of compound **4e**

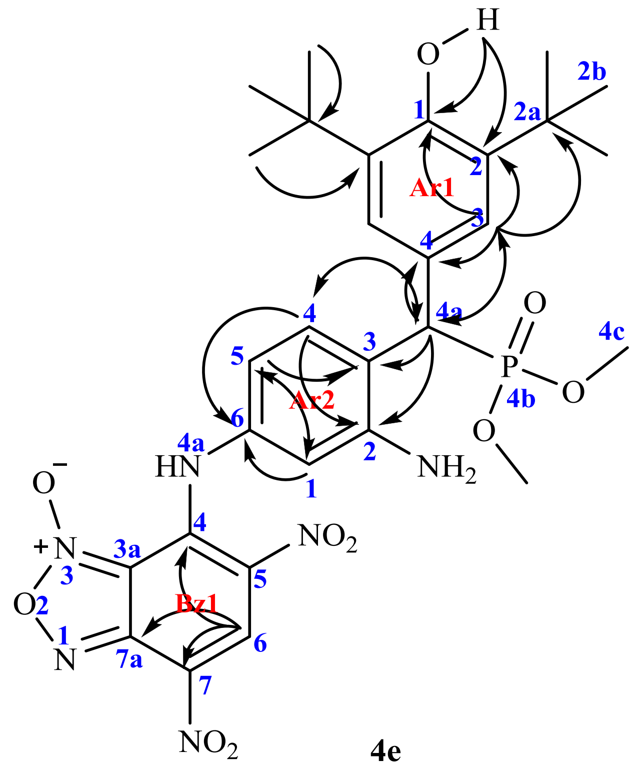

4e

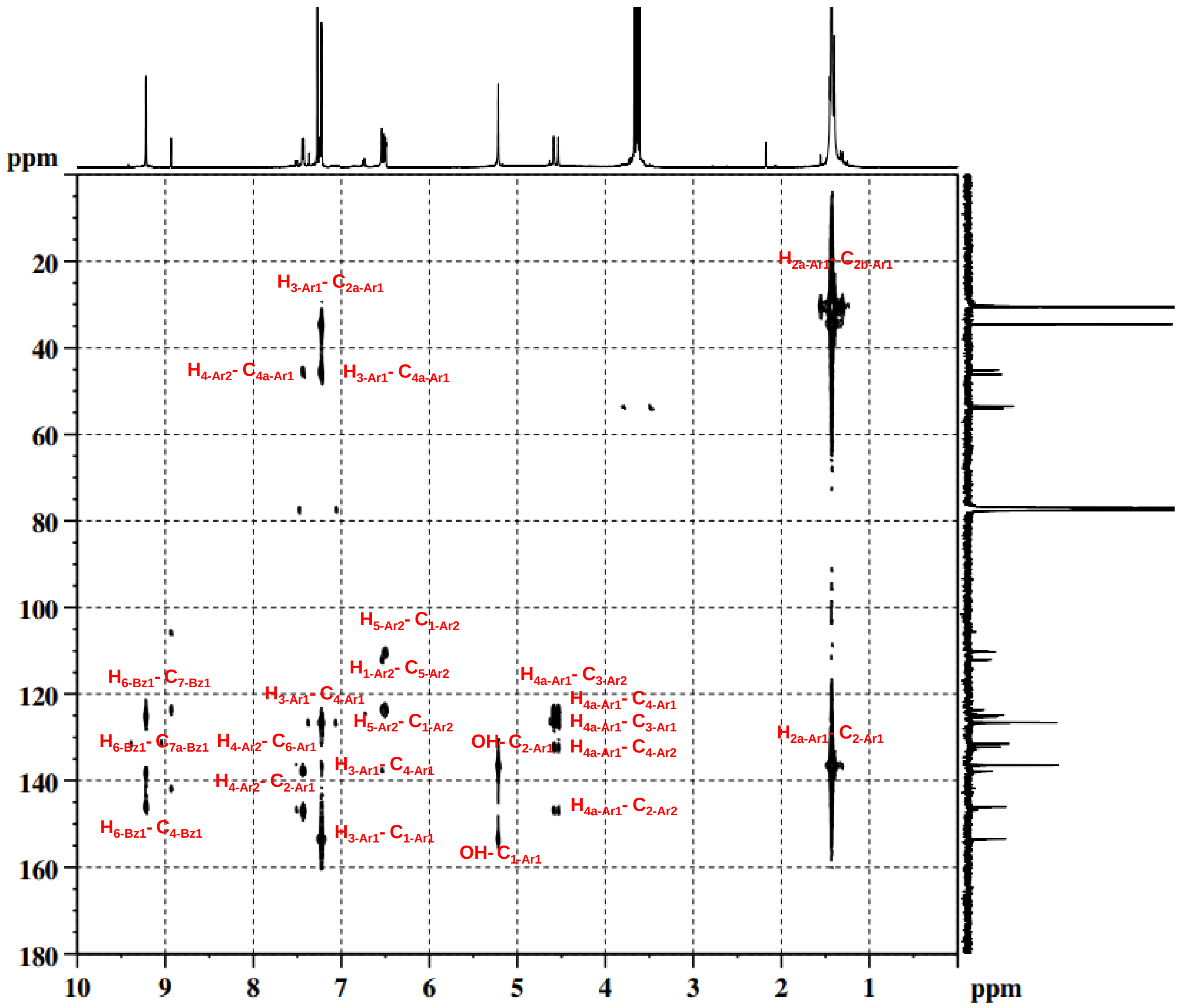

Figure S18. ^{1}H-^{13}C HMBC ($CDCl_3$, 126 MHz, 303 K) of compound **4e**

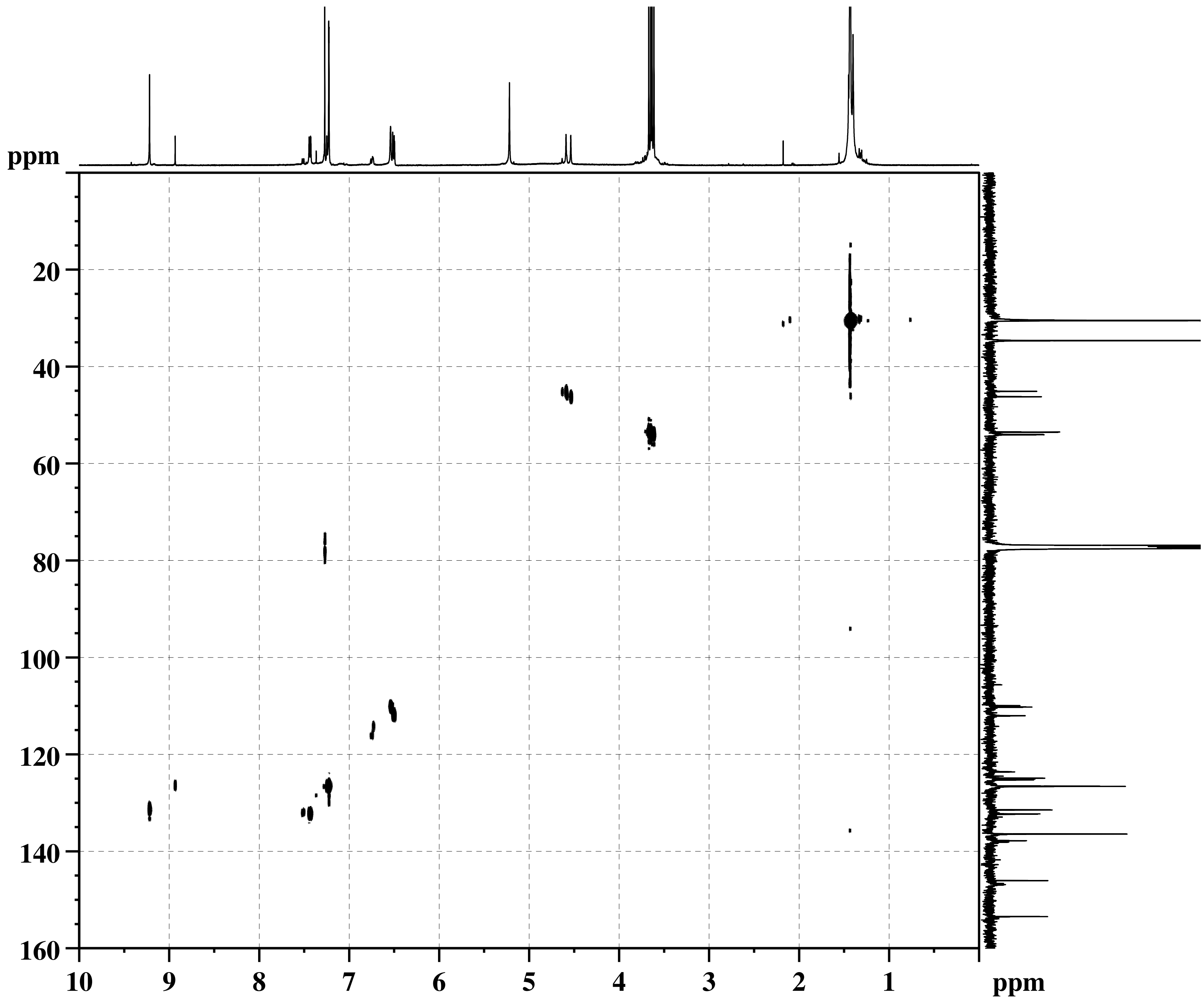

Figure S19. ^{1}H-^{13}C HSQC (CDCl$_3$, 126 MHz, 303 K) of compound **4e**

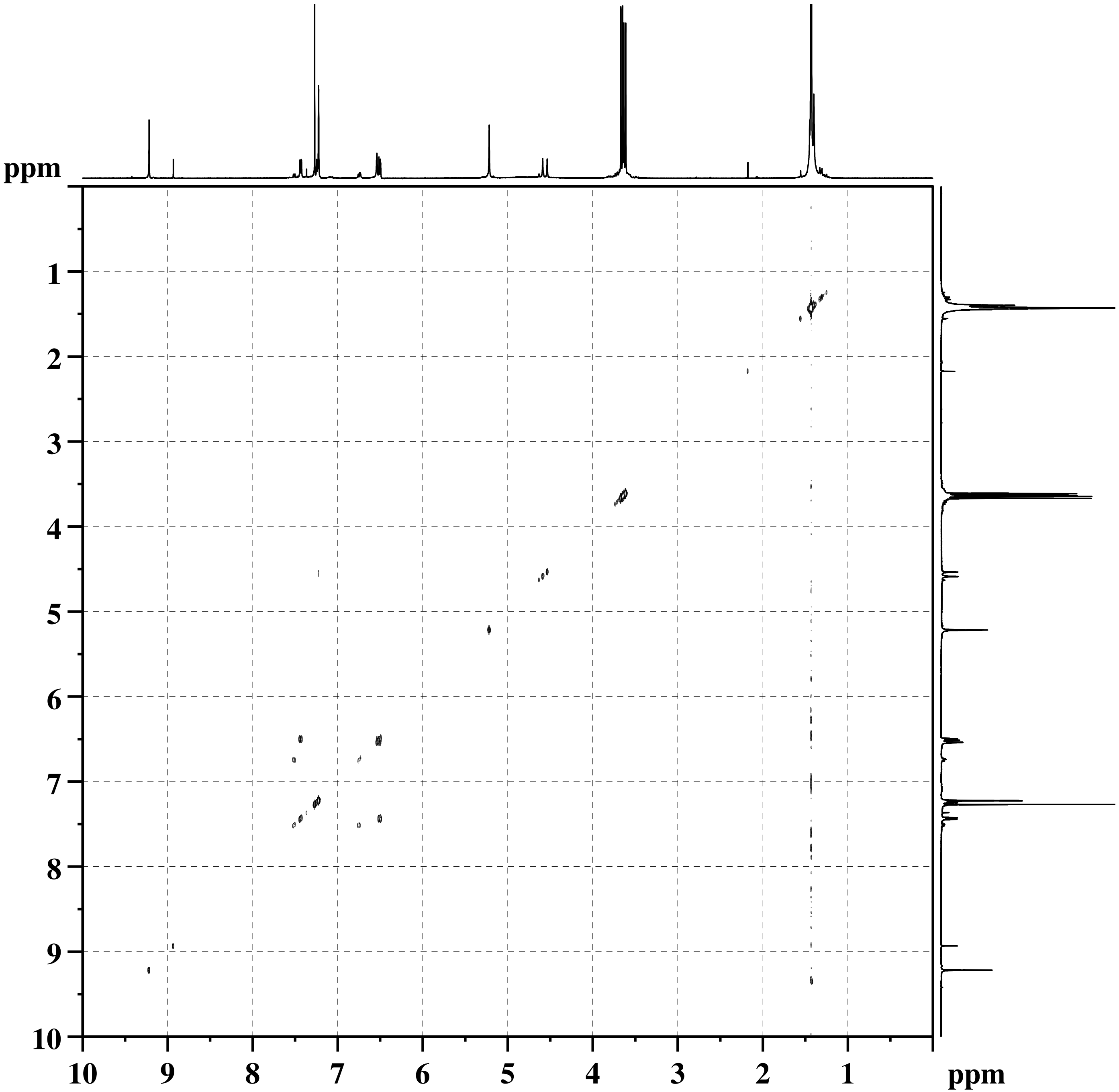

Figure S20. COSY (CDCl$_3$, 126 MHz, 303 K) of compound **4e**

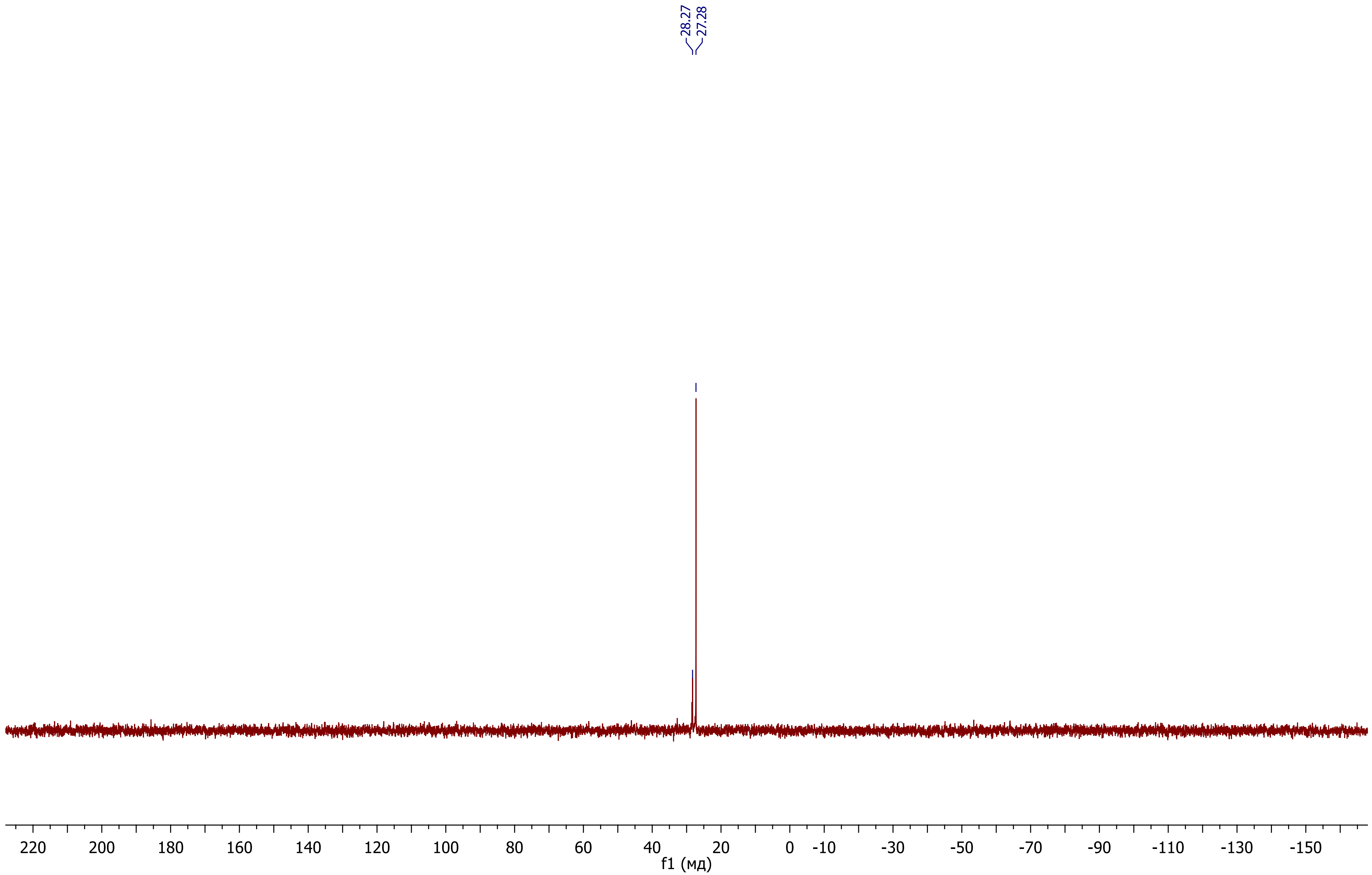

Figure S21. ^{31}P NMR ($CDCl_3$, 162 Hz, 303 K) of compound **4e**

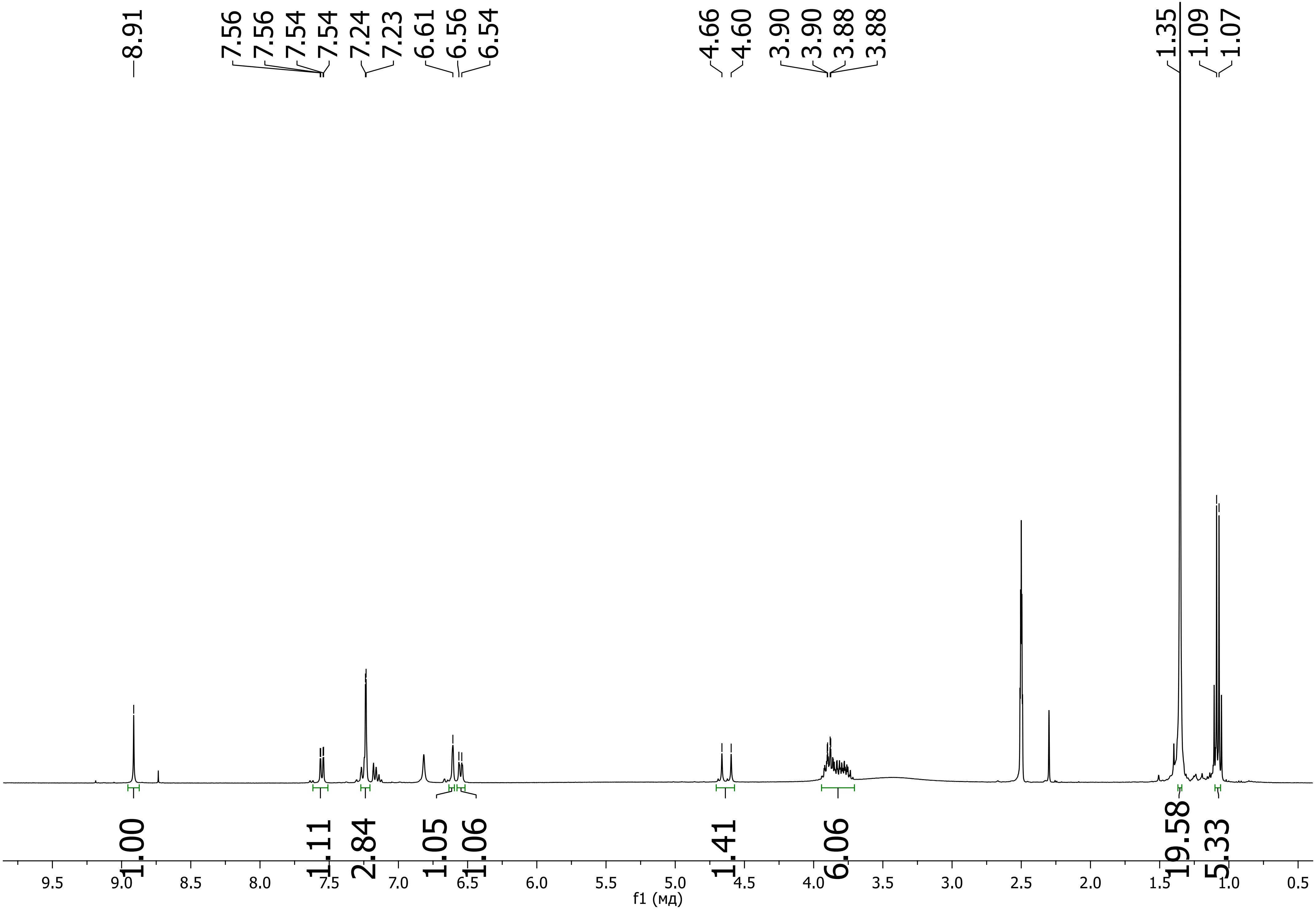

Figure S22. ^{1}H NMR (DMSO-d_6, 400 MHz, 303 K) of compound **4f**

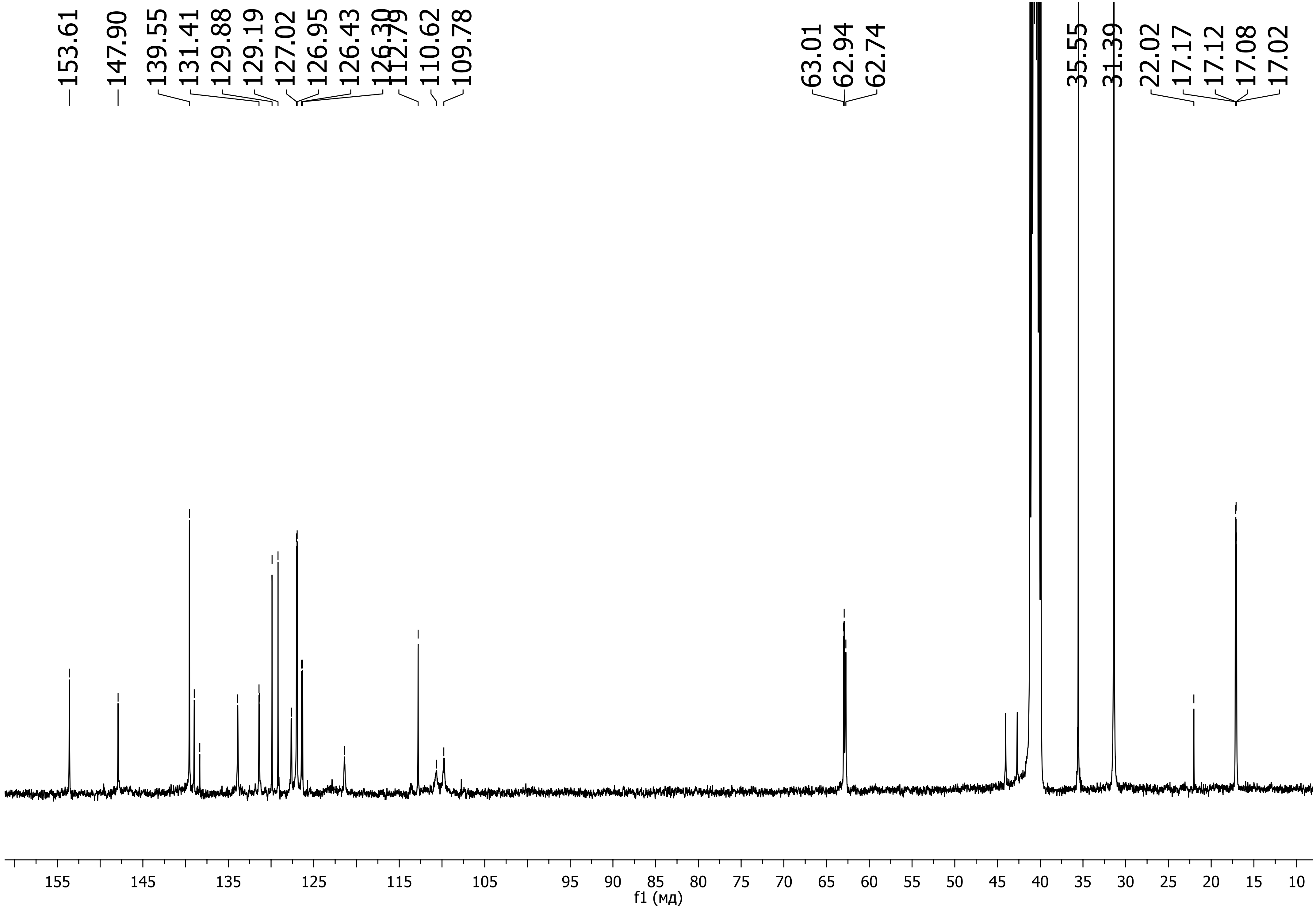

Figure S23. ^{13}C NMR (DMSO-d_6, 101 MHz, 303 K) of compound **4f**

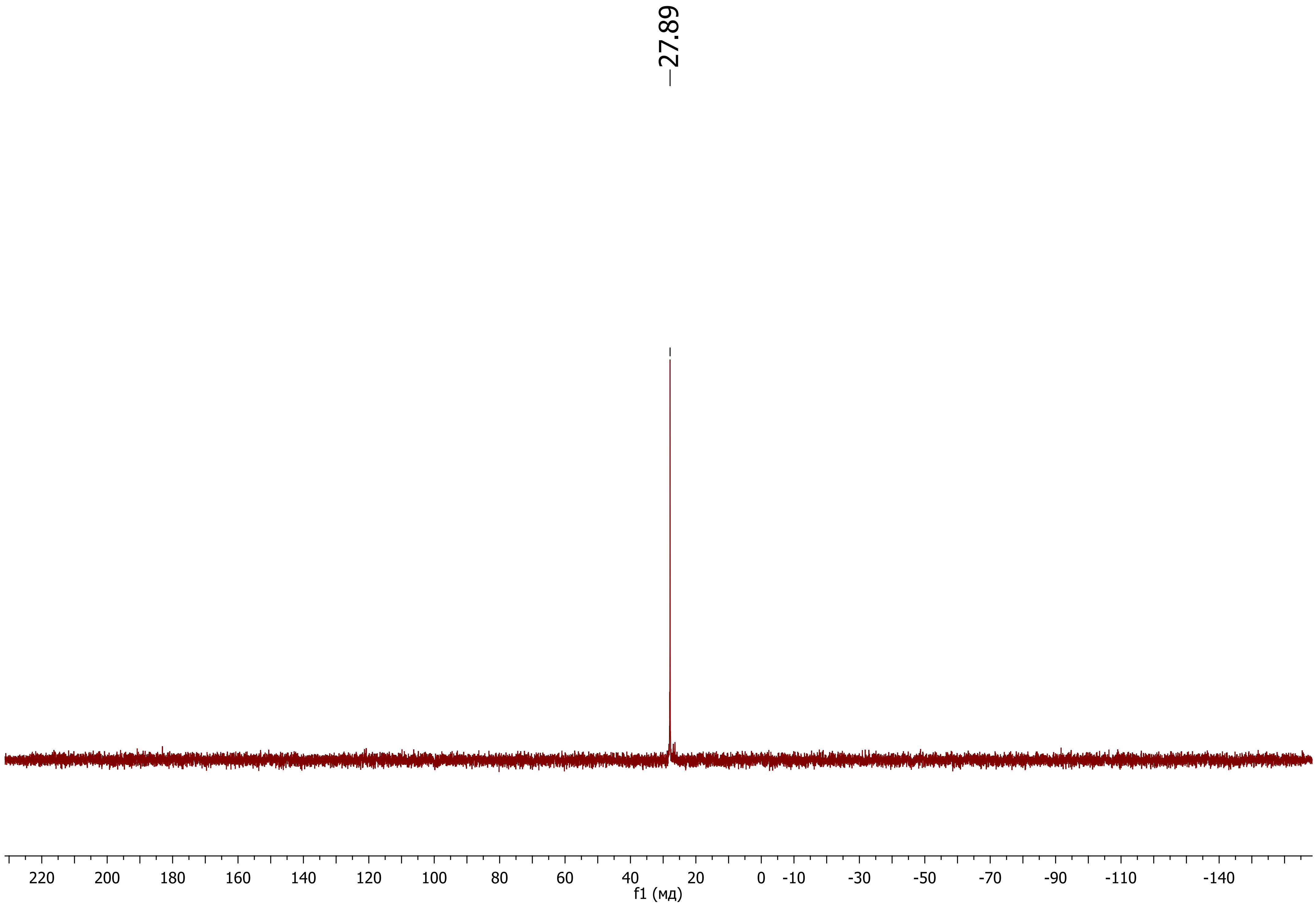

Figure S24. ^{31}P NMR (DMSO-d_6, 162 Hz, 303 K) of compound **4f**

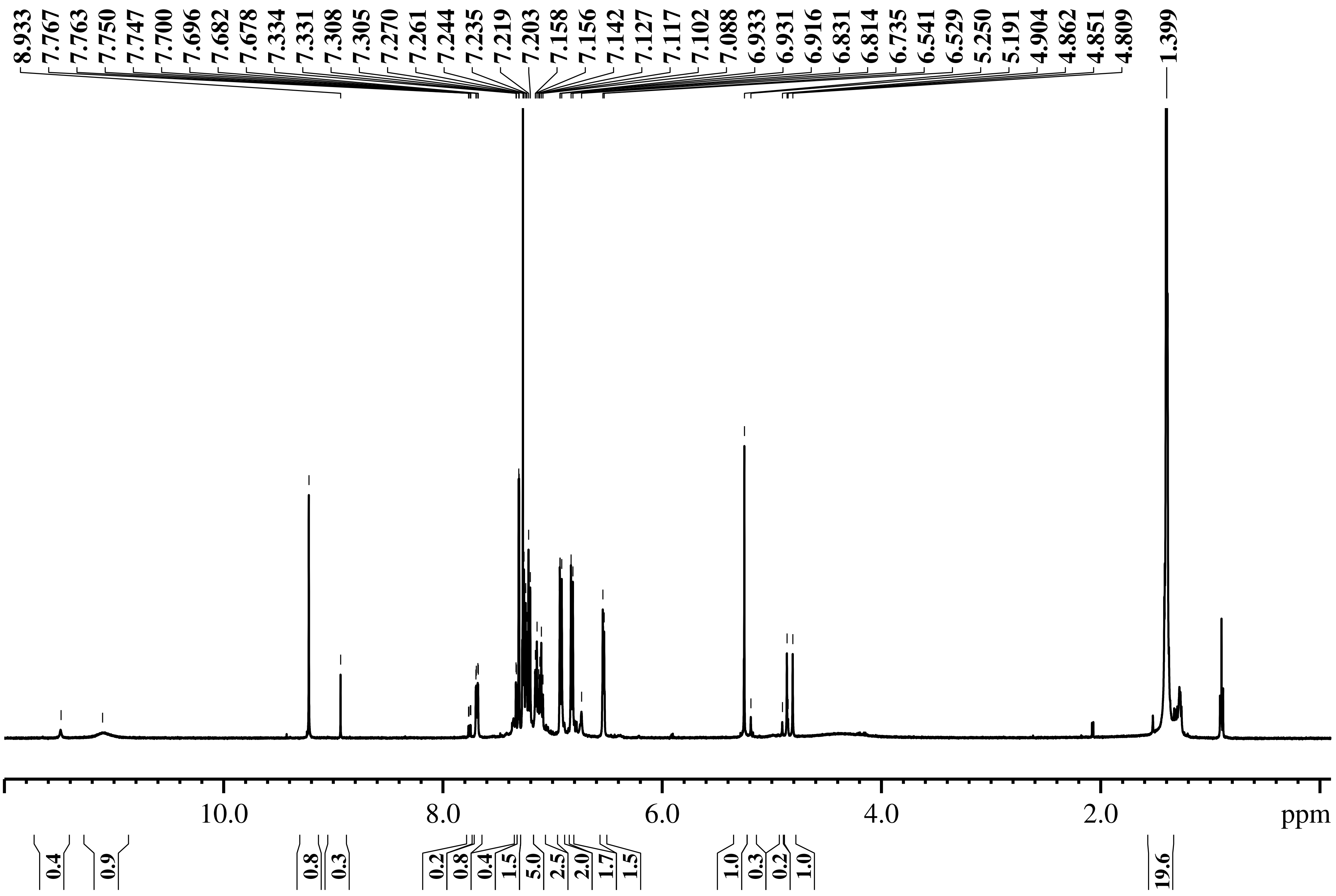

Figure S25. ^{1}H NMR ($CDCl_3$, 500 MHz, 303 K) of compound **4g**

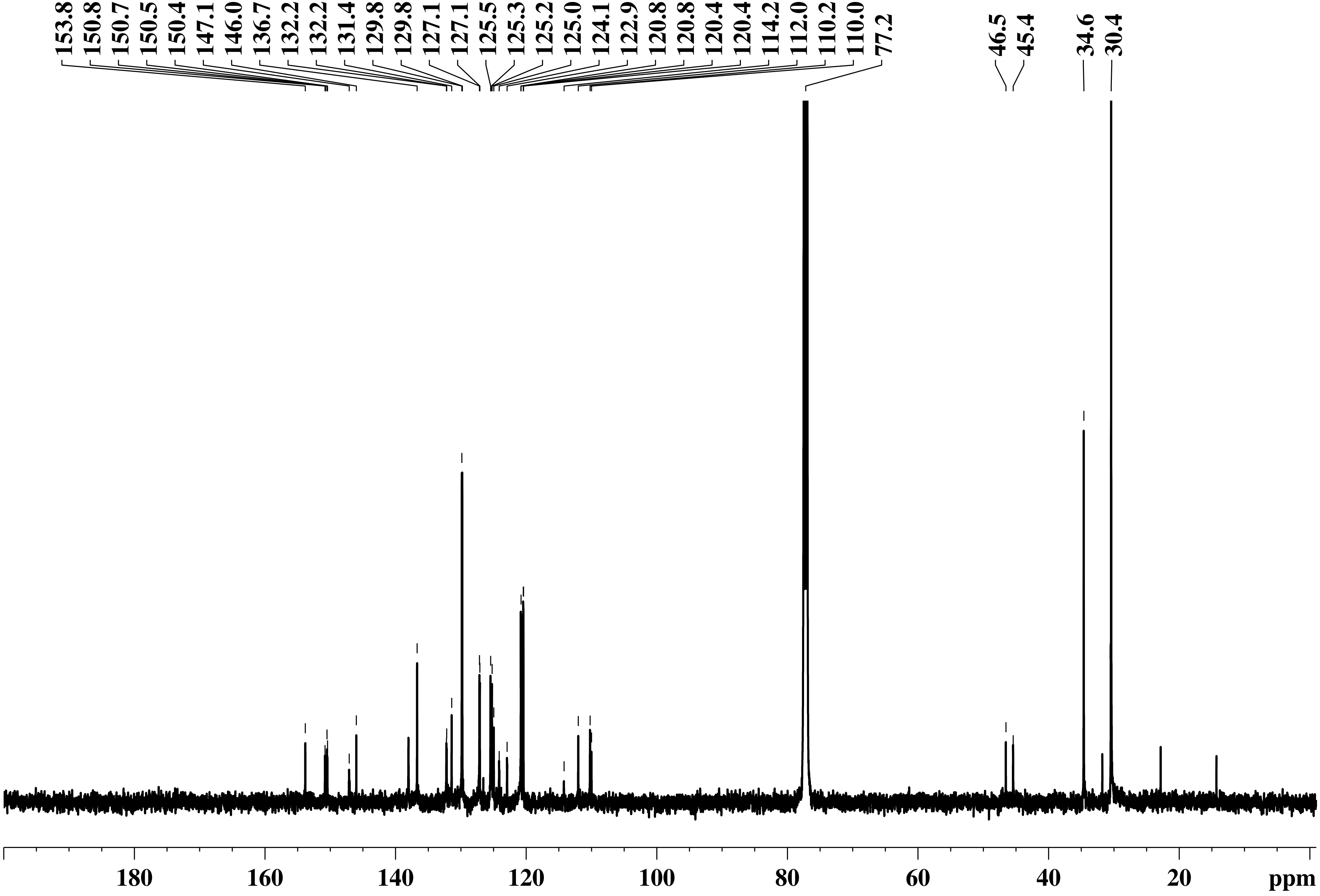

Figure S26. ^{13}C NMR ($CDCl_3$, 126 MHz, 303 K) of compound **4g**

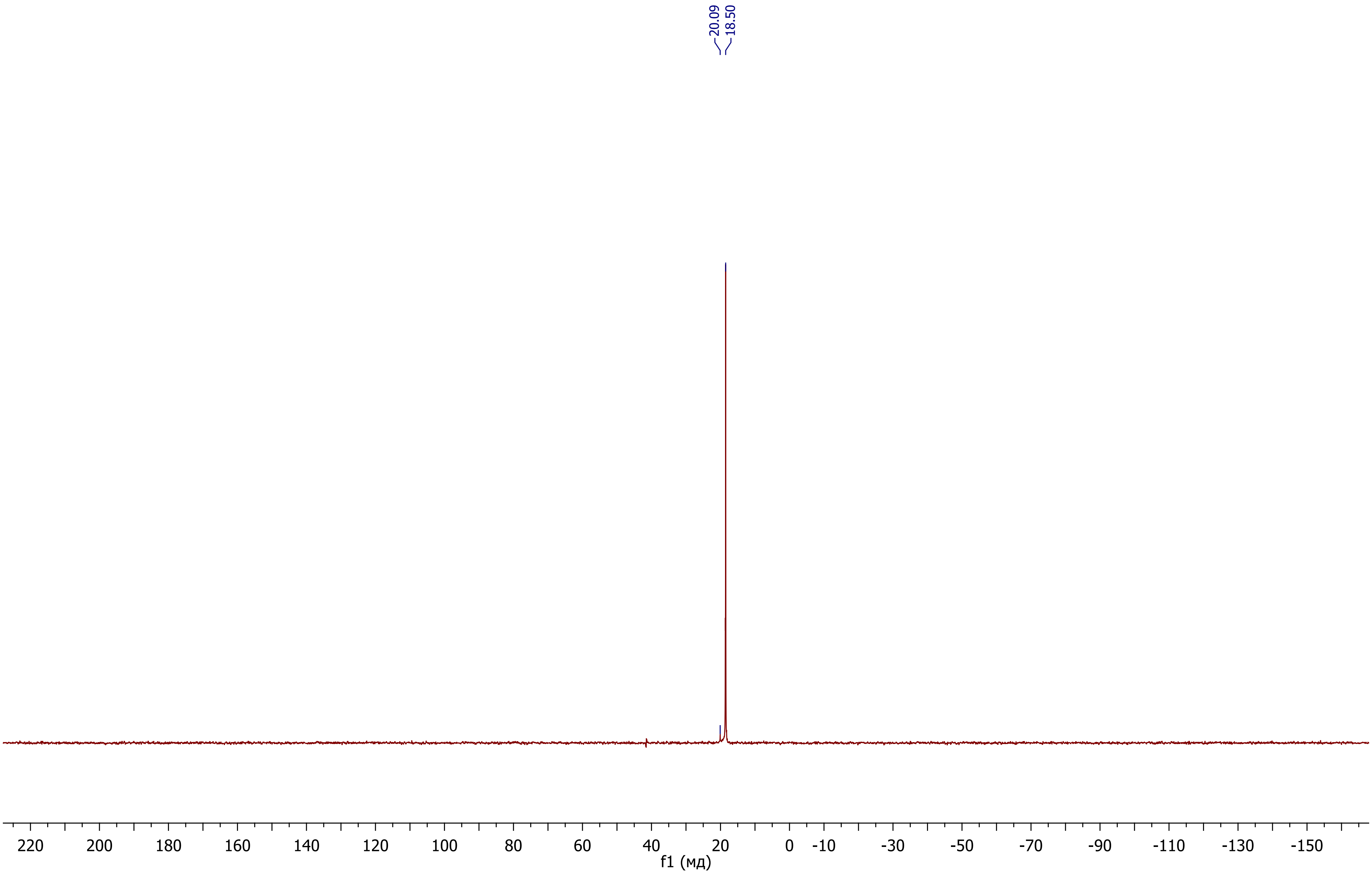

Figure S27. ^{31}P NMR ($CDCl_3$, 162 Hz, 303 K) of compound **4g**

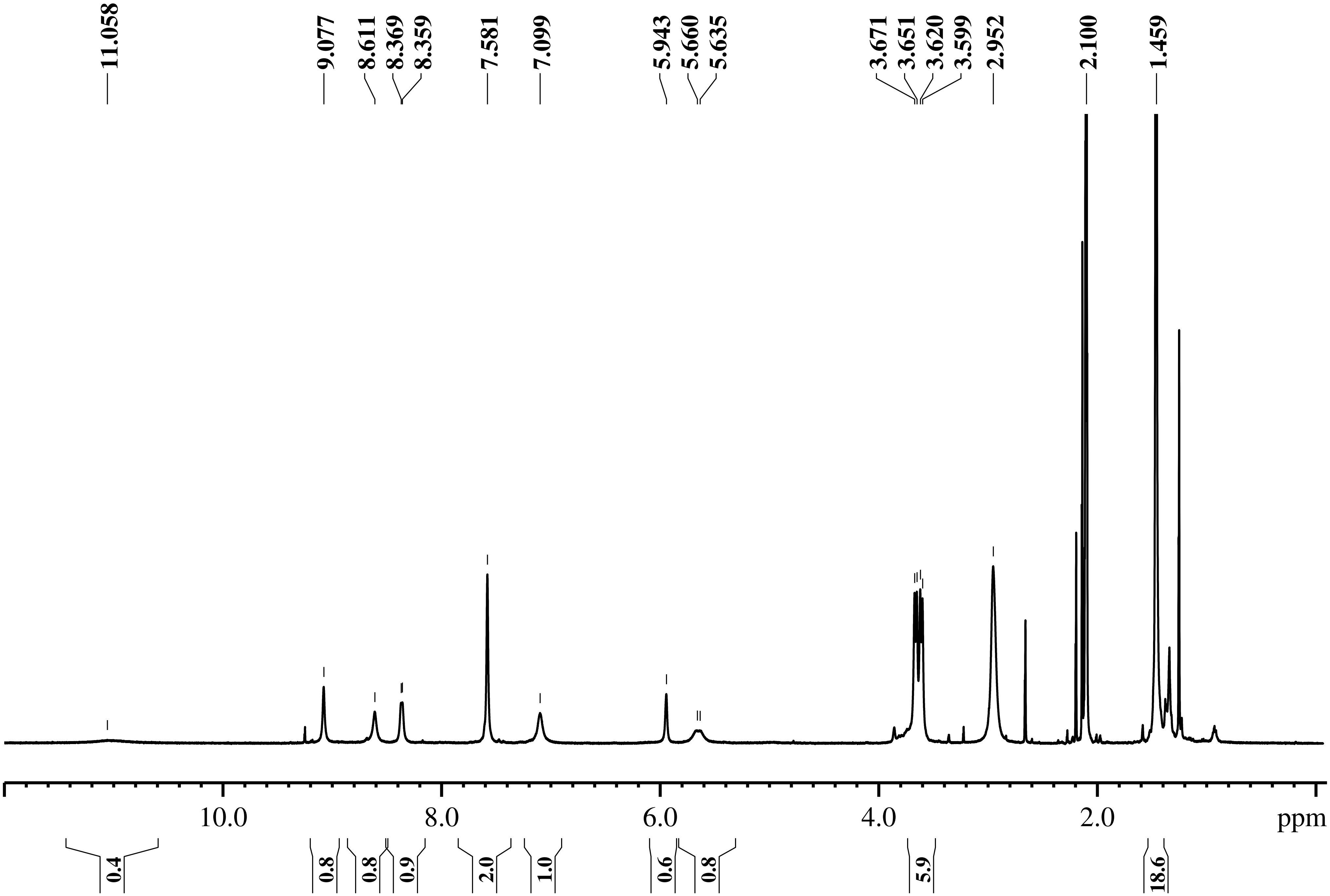

Figure S28. ^{1}H NMR (acetone-d_6, 500 MHz, 303 K) of compound **5a**

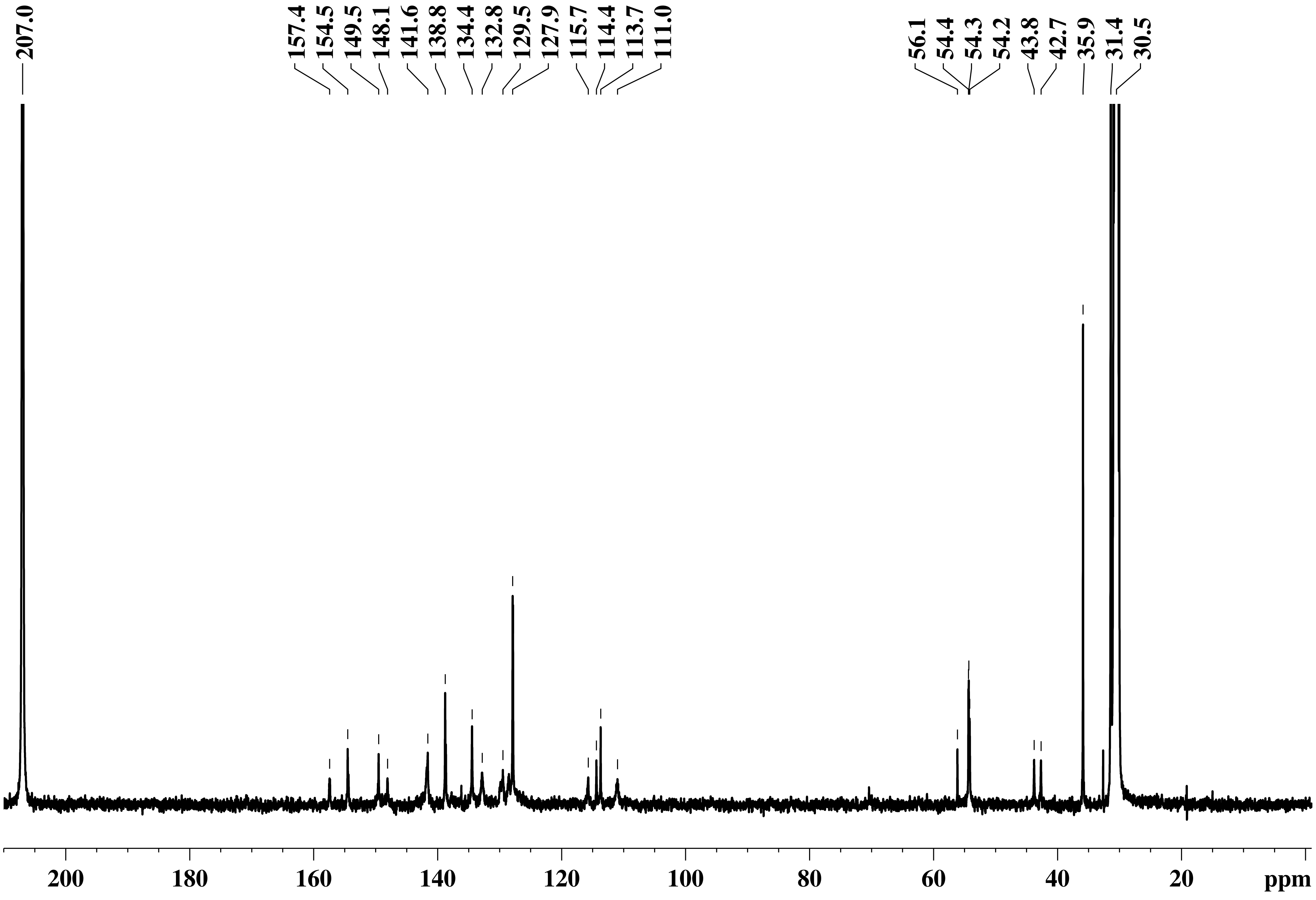

Figure S29. ^{13}C NMR (acetone-d6, 126 MHz, 303 K) of compound **5a**

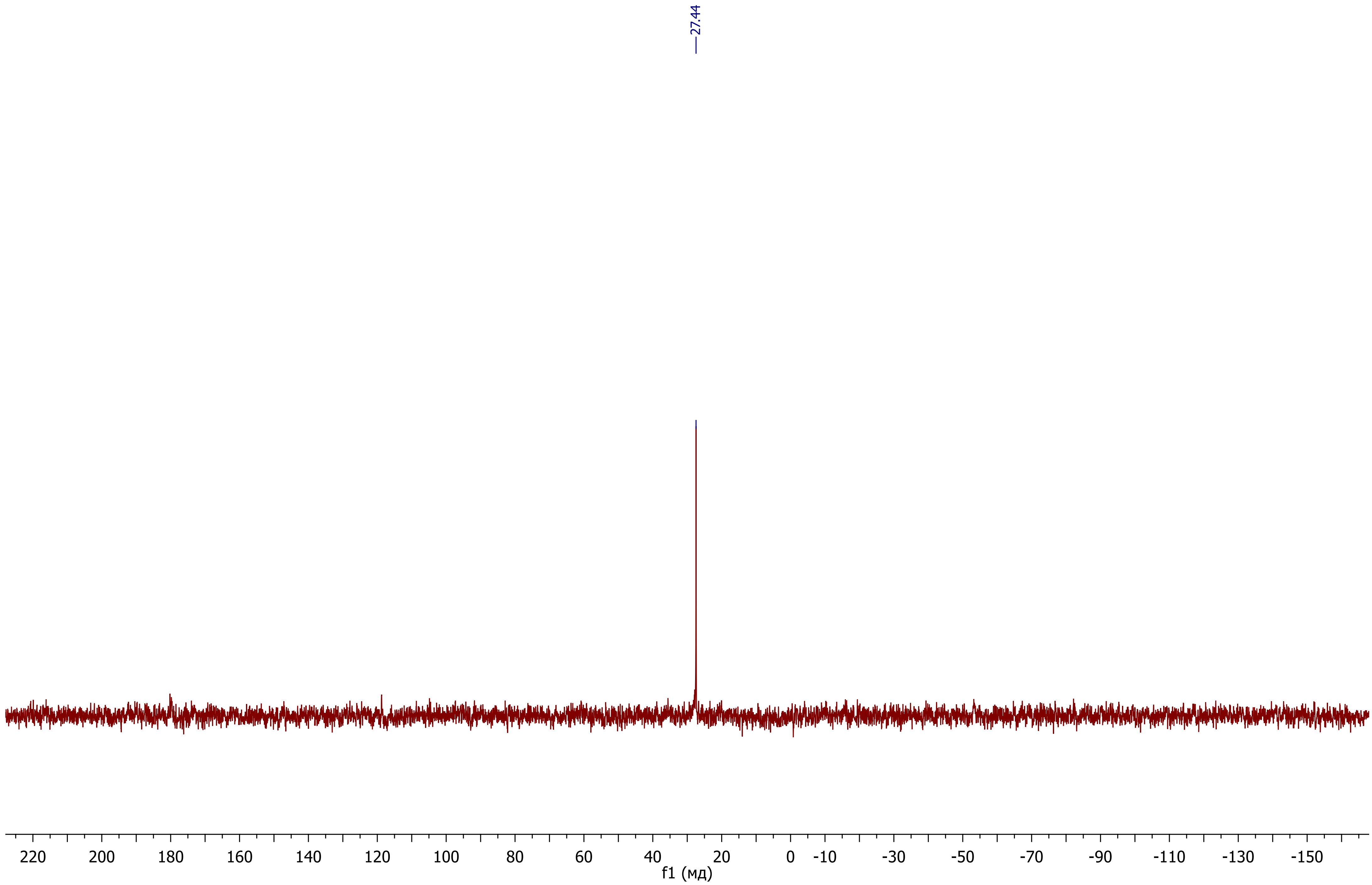

Figure S30. ^{31}P NMR ($CDCl_3$, 162 Hz, 303 K) of compound **5a**

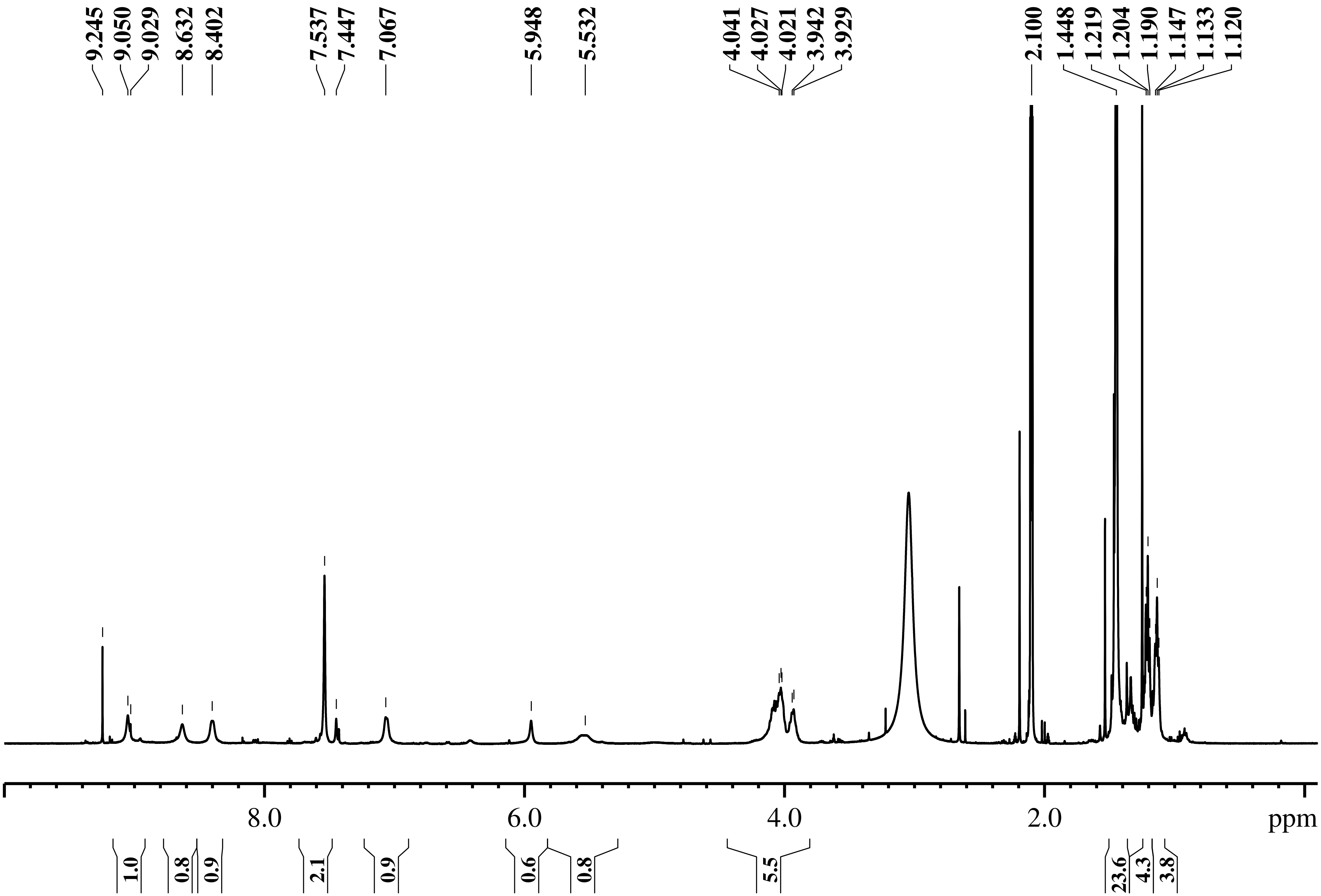

Figure S31. ^{1}H NMR (acetone-d_6, 500 MHz, 303 K) of compound **5b**

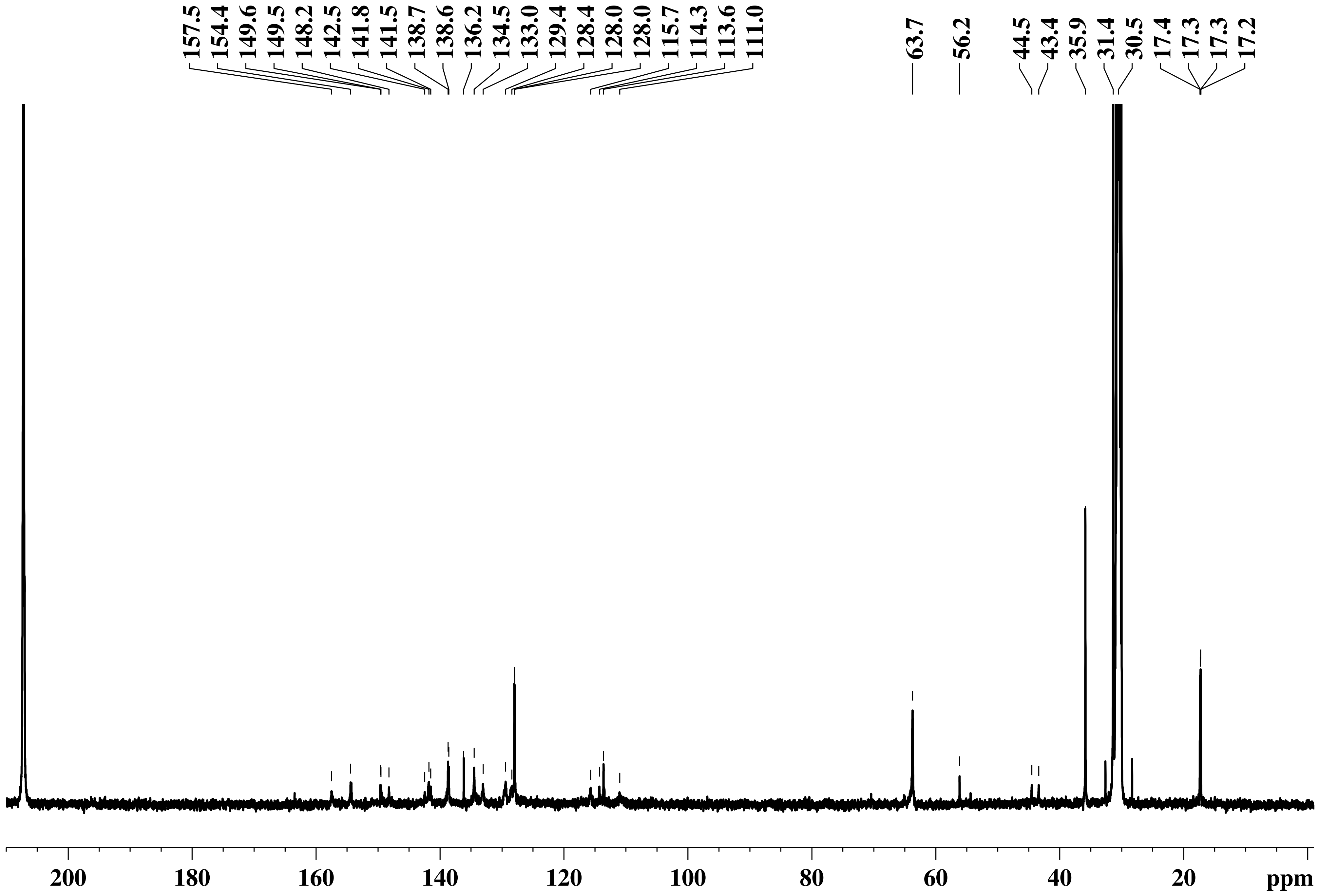

Figure S32. ^{13}C NMR (acetone-d_6, 126 MHz, 303 K) of compound **5b**

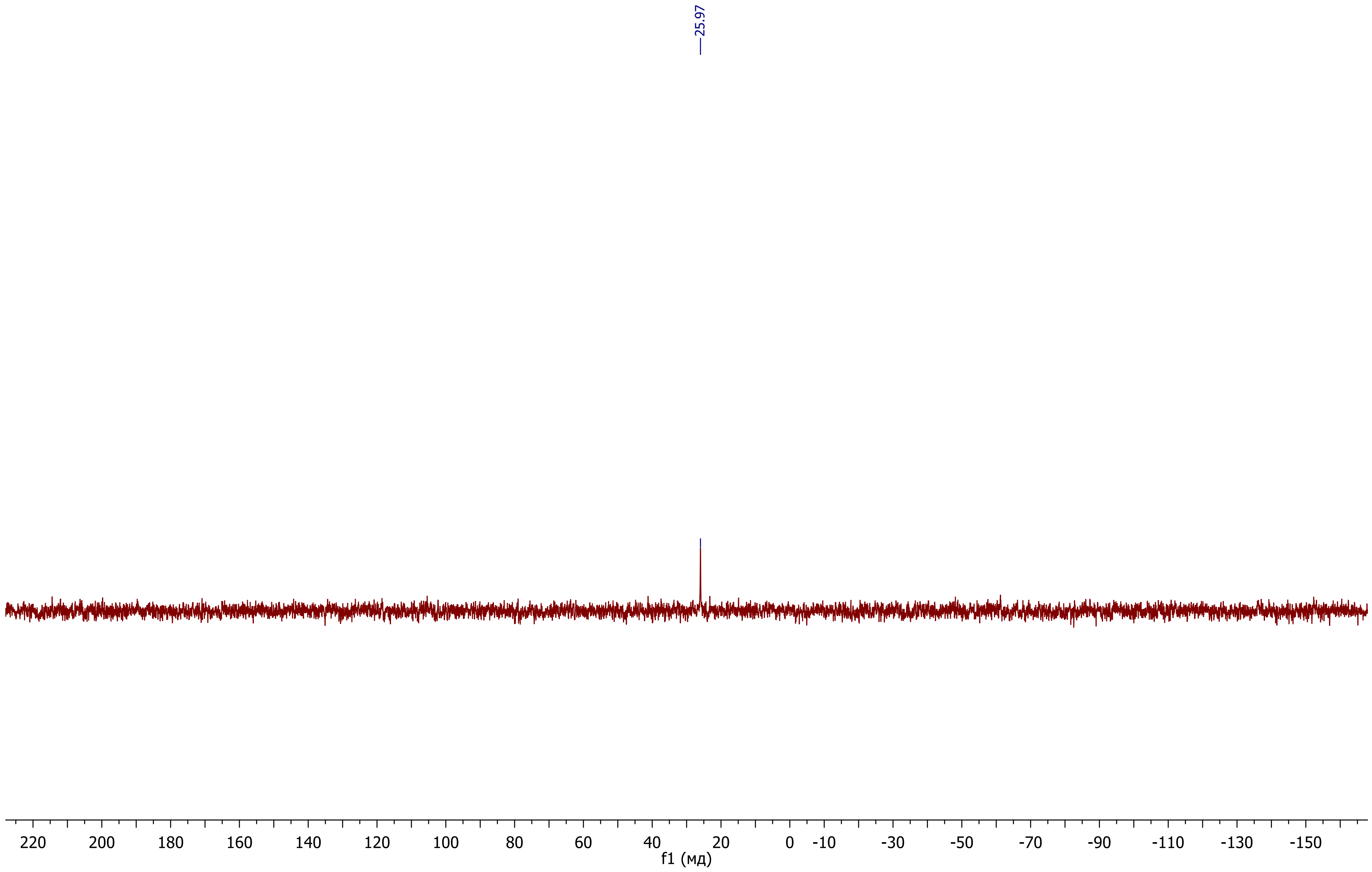

Figure S33. ${}^{31}P$ NMR (acetone-d_6, 162 Hz, 303 K) of compound **5b**

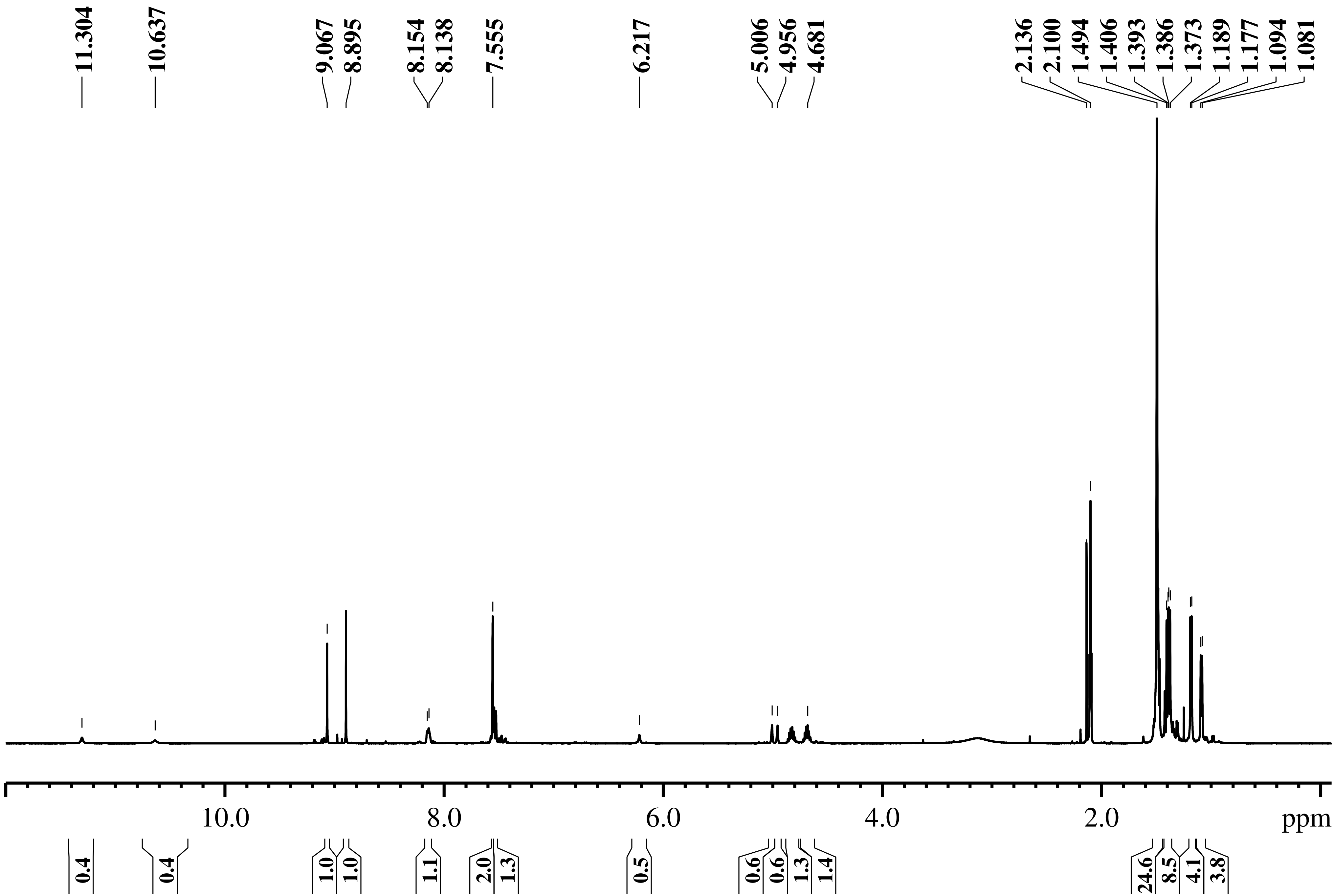

Figure S34. ^{1}H NMR (acetone-d_6, 500 MHz, 303 K) of compound **5c**

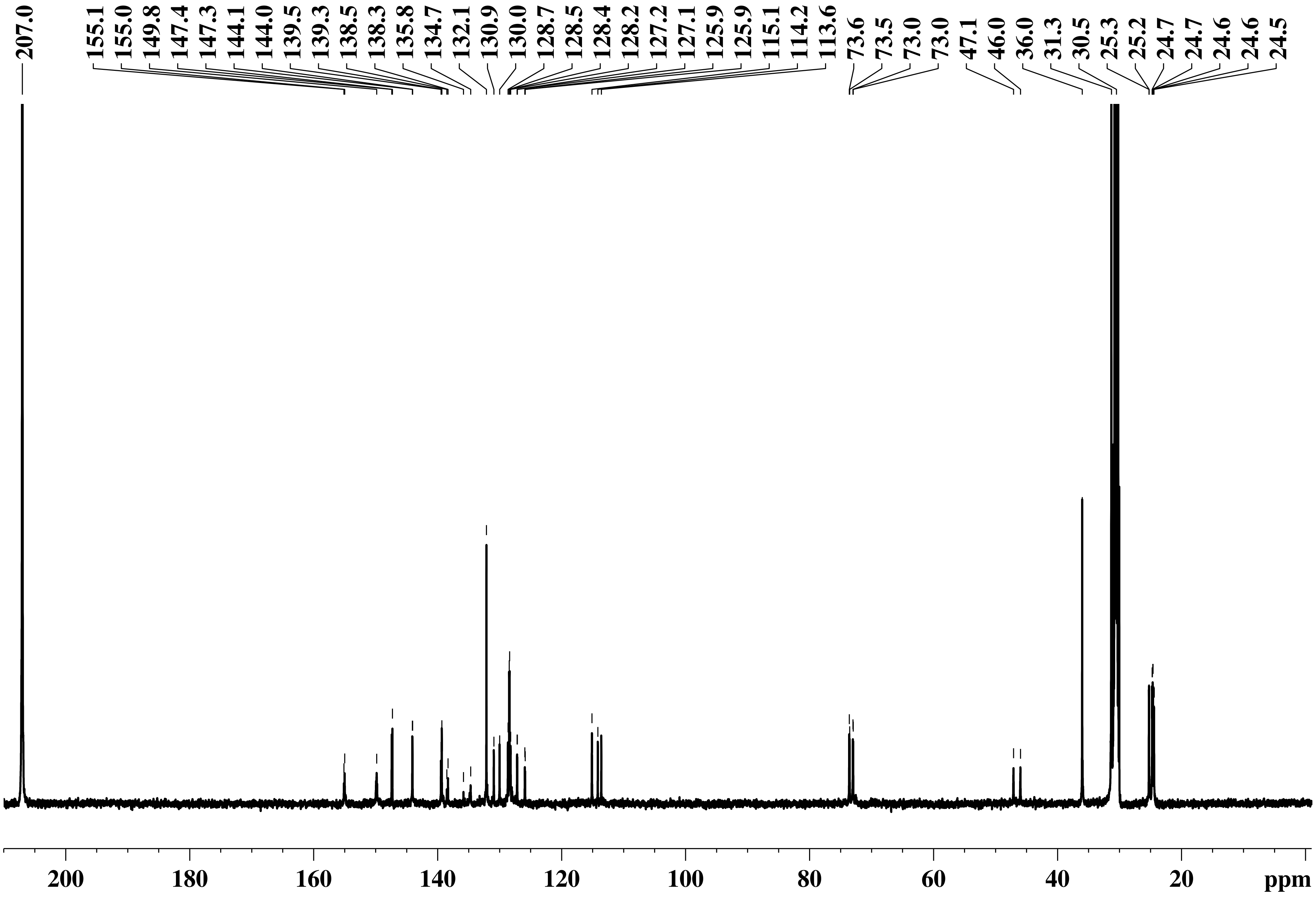

Figure S35. ^{13}C NMR (acetone-d_6, 126 MHz, 303 K) of compound **5c**

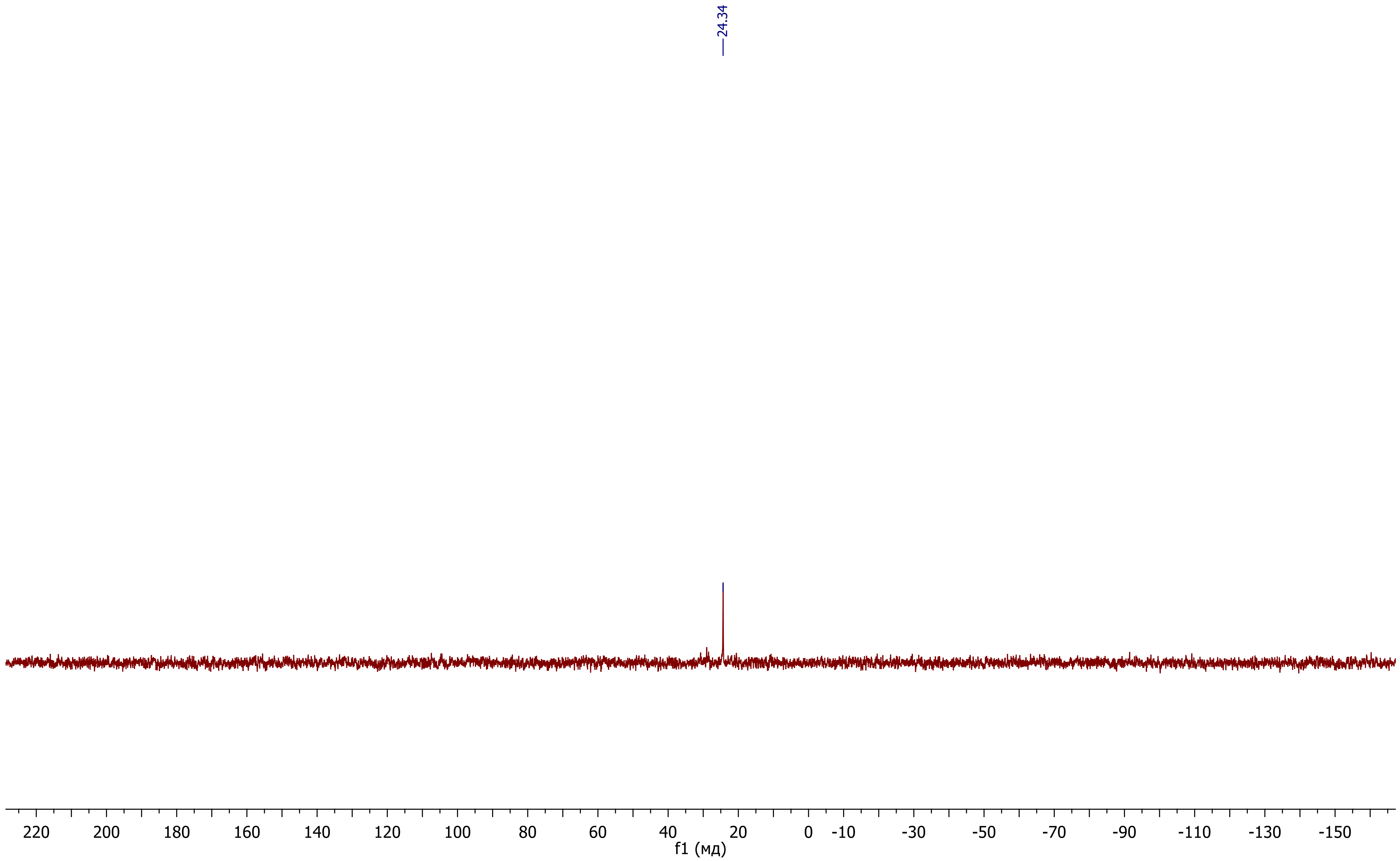

Figure S36. ^{31}P NMR (acetone-d_6, 162 Hz, 303 K) of compound **5c**

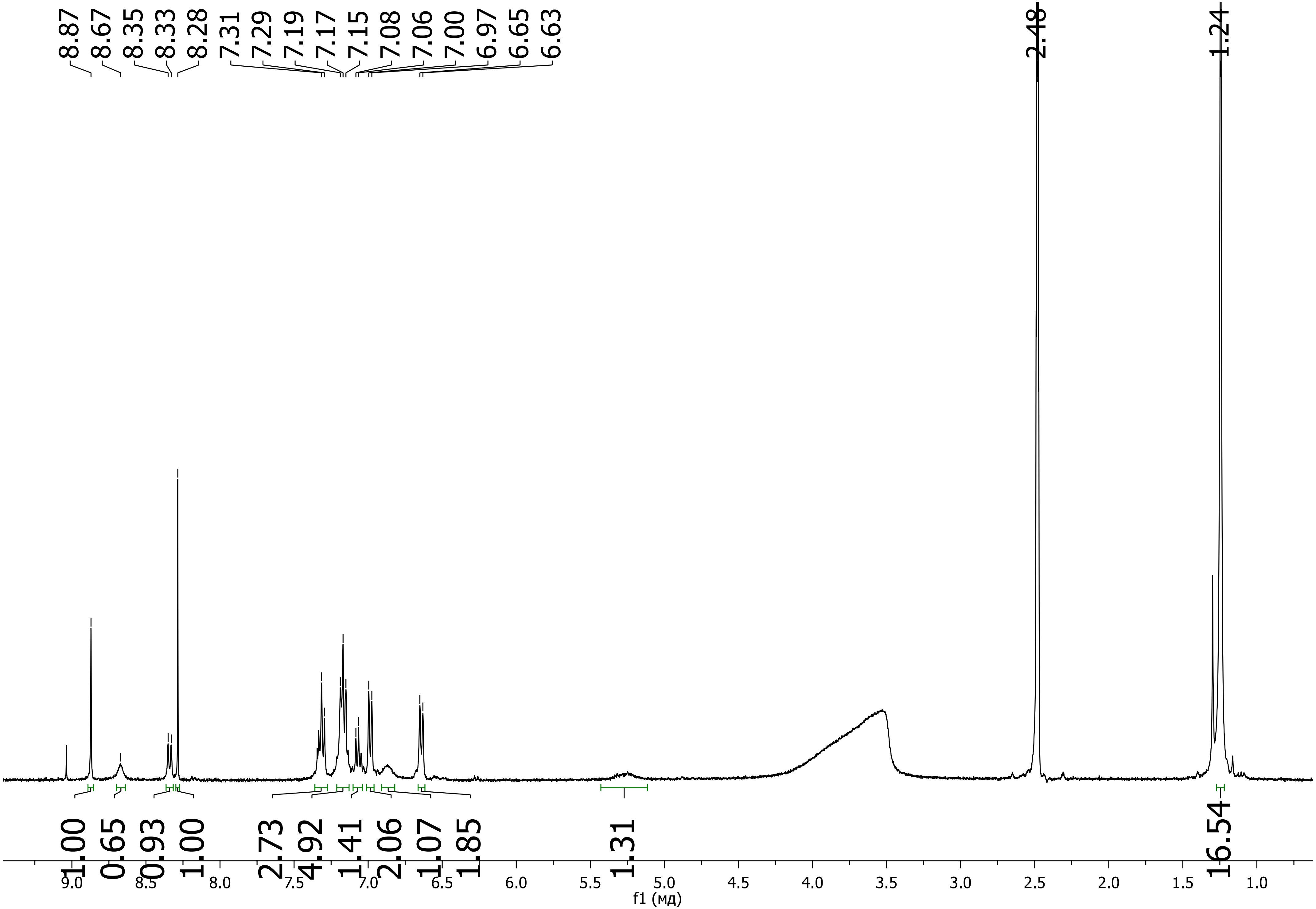

Figure S37. ^{1}H NMR (DMSO-d_6, 400 MHz, 303 K) of compound **5d**

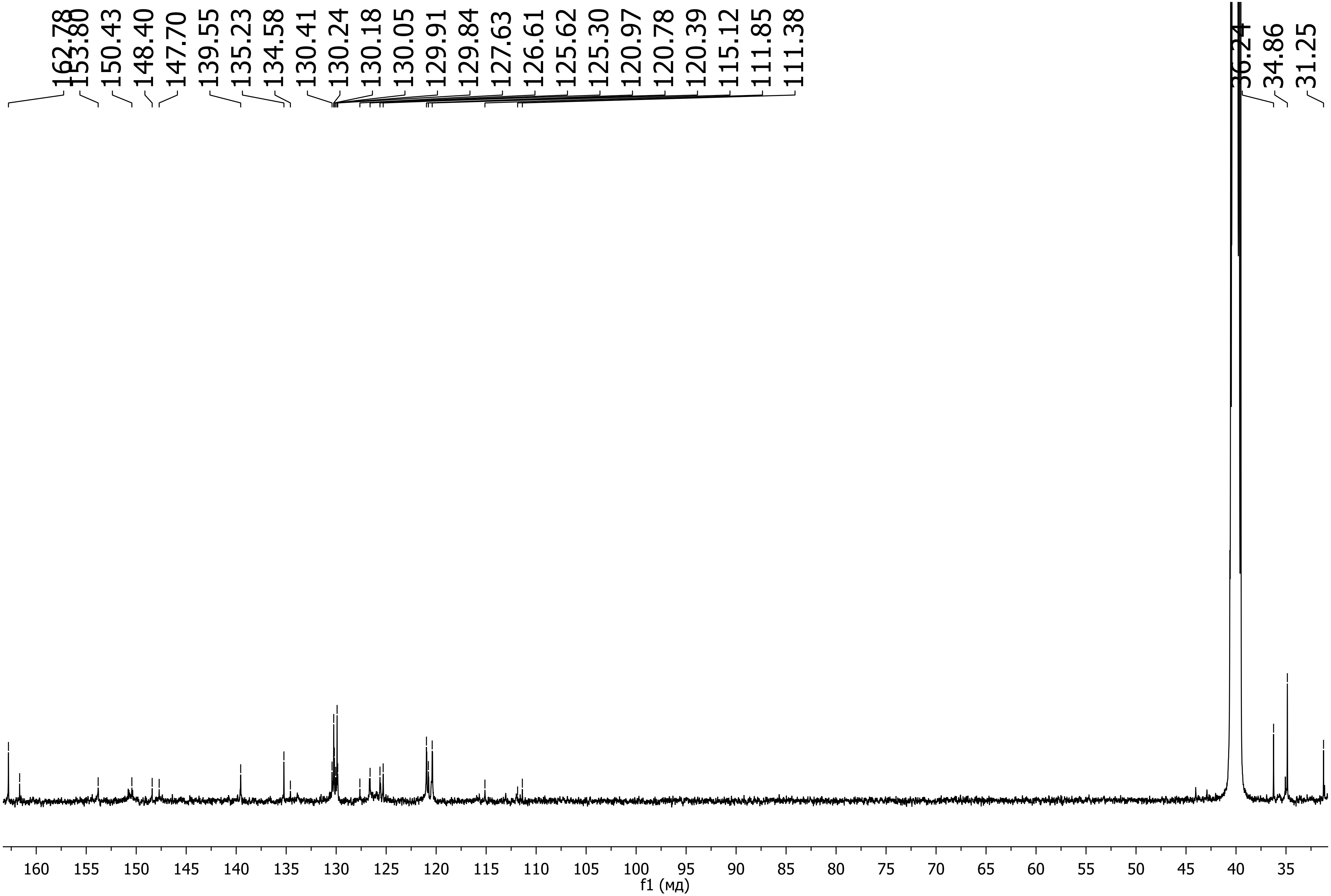

Figure S38. ^{13}C NMR (DMSO-d_6, 126 MHz, 303 K) of compound **5d**

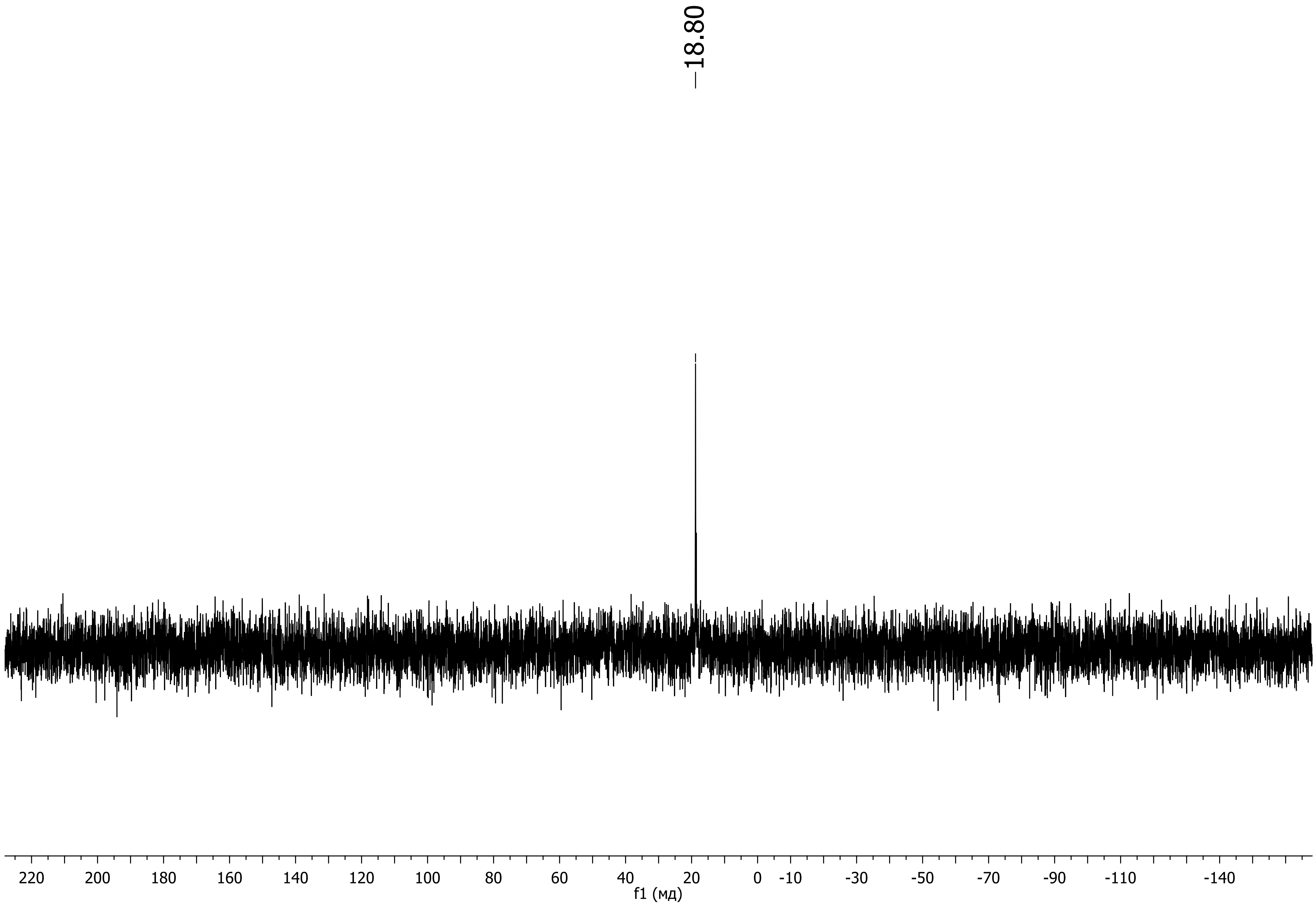

Figure S39. ^{31}P NMR (DMSO-d_6, 162 MHz, 303 K) of compound **5d**

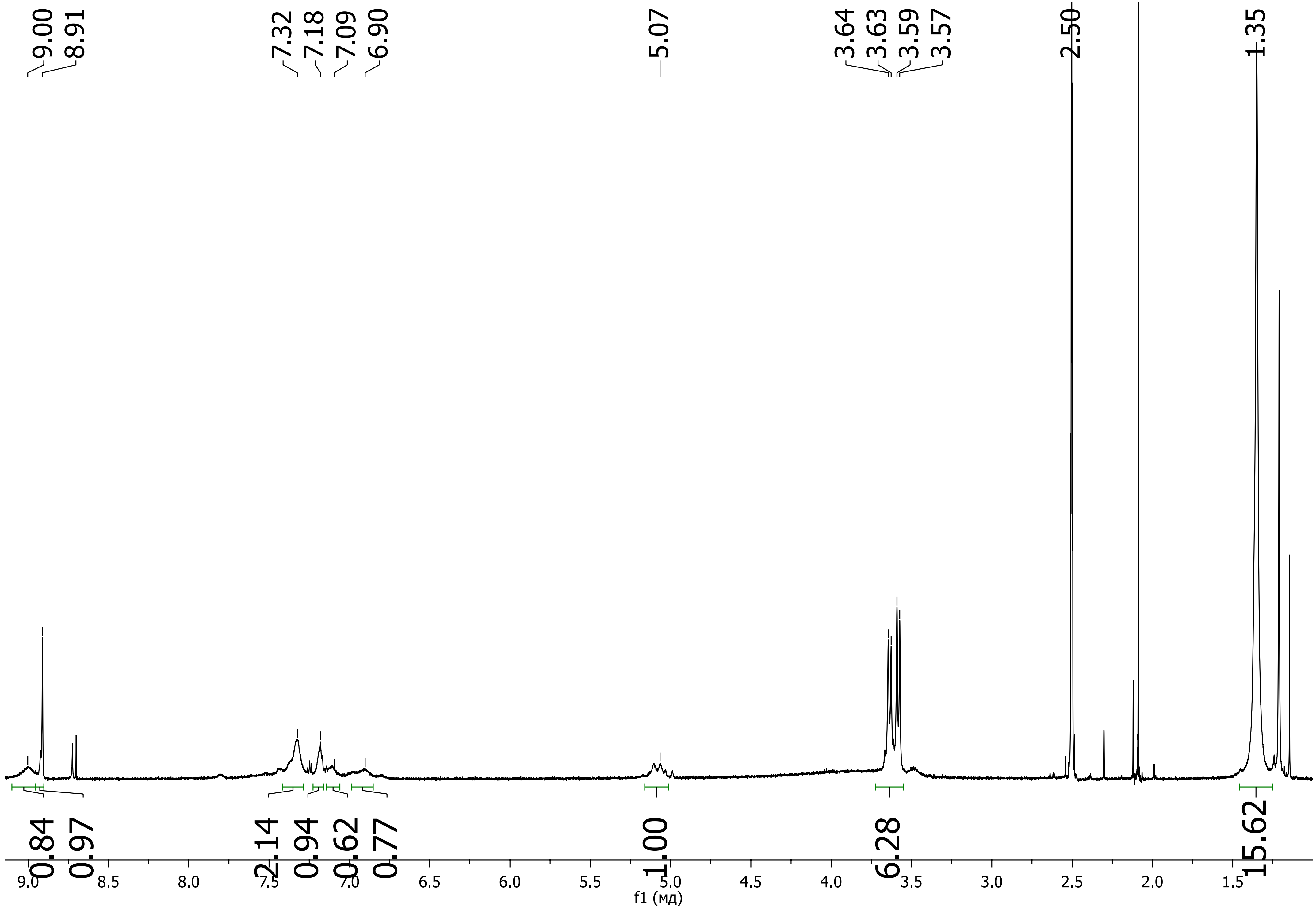

Figure S40. ^{1}H NMR (DMSO-d_6, 600 MHz, 303 K) of compound **5e**

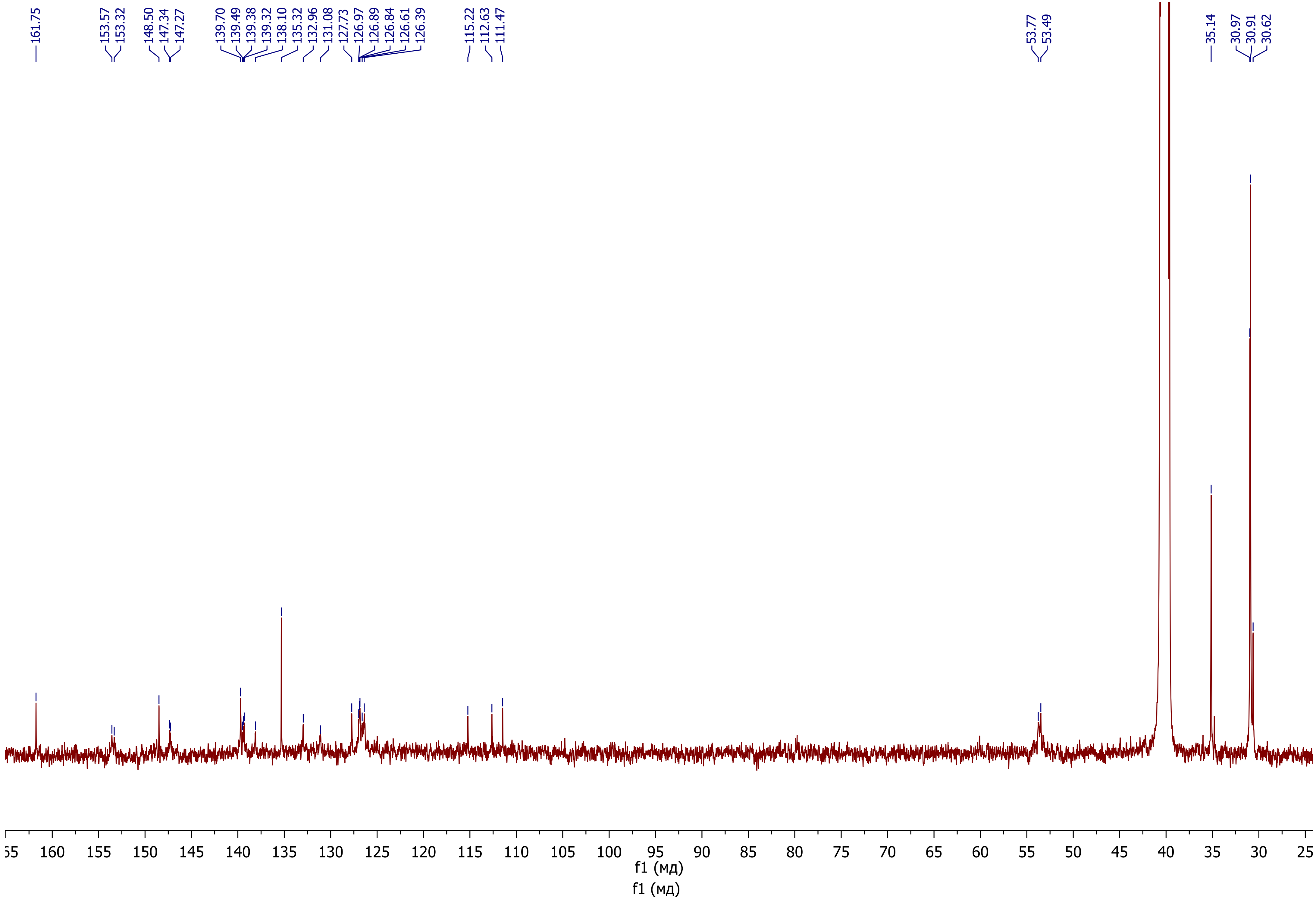

Figure S41. ^{13}C NMR (DMSO-d_6, 126 MHz, 303 K) of compound **5e**

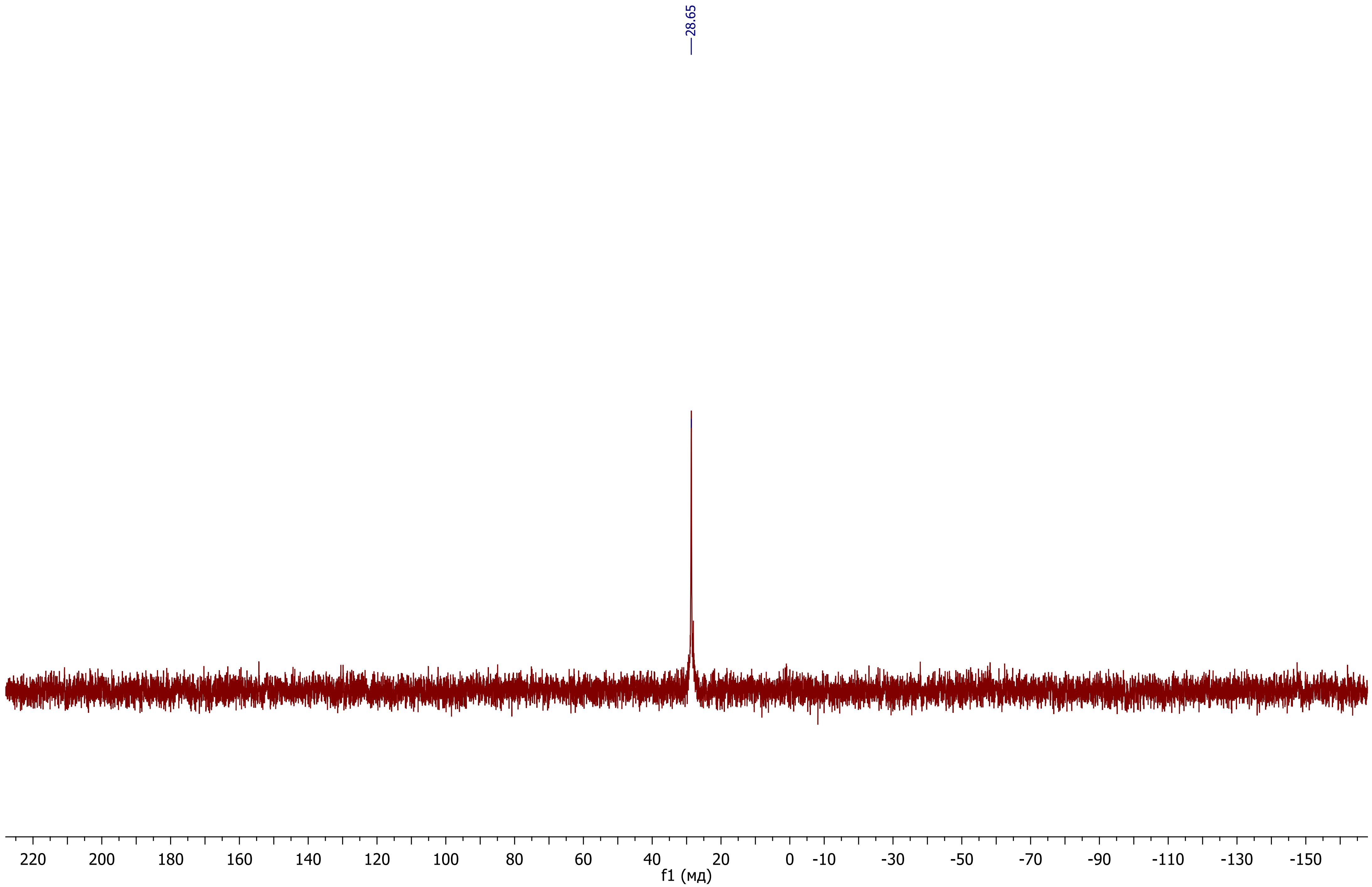

Figure S42 ^{31}P NMR (DMSO-d_6, 162 Hz, 303 K) of compound **5e**

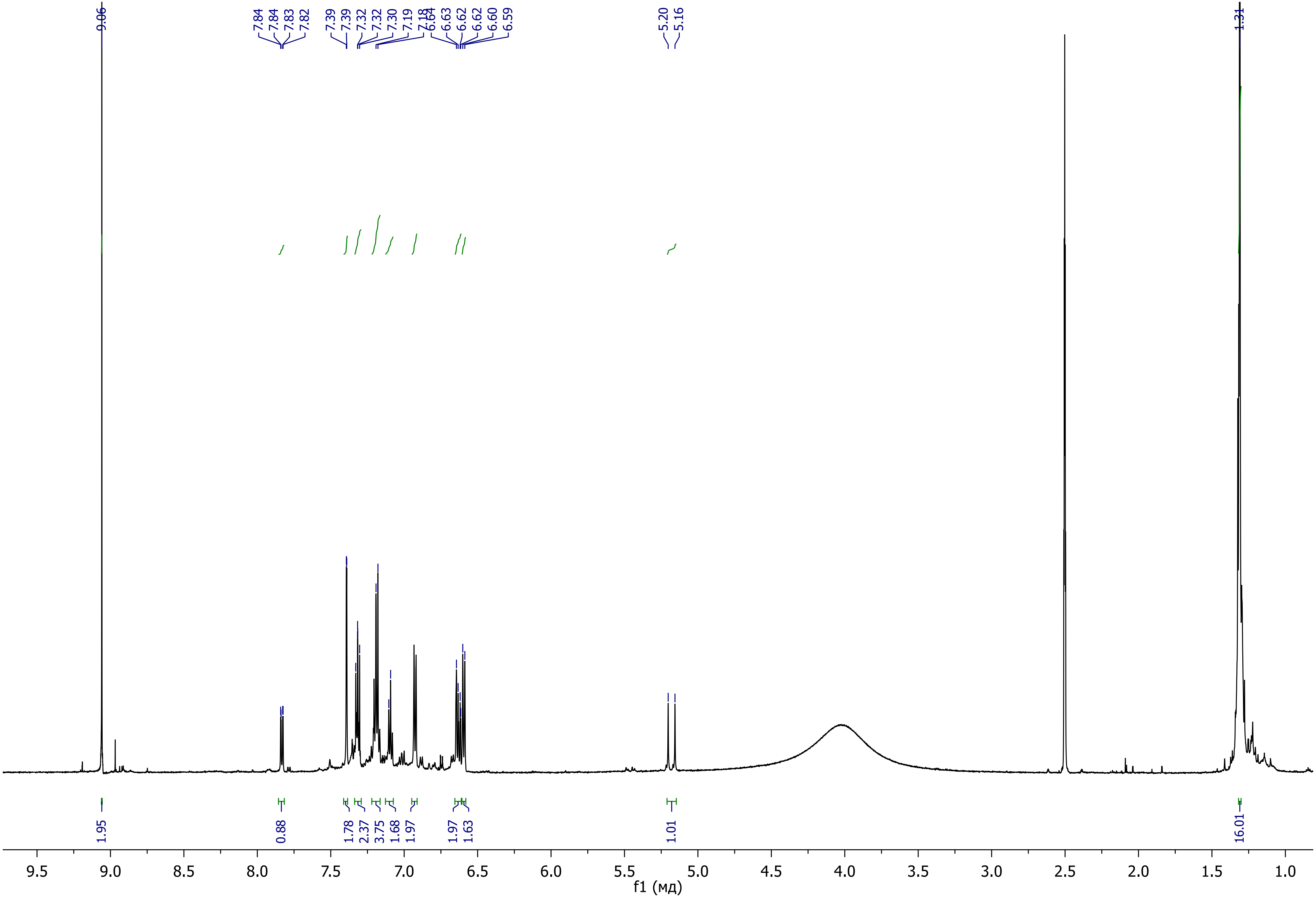

Figure S43. ^{1}H NMR (DMSO-d_6, 400 MHz, 303 K) of compound **5g**

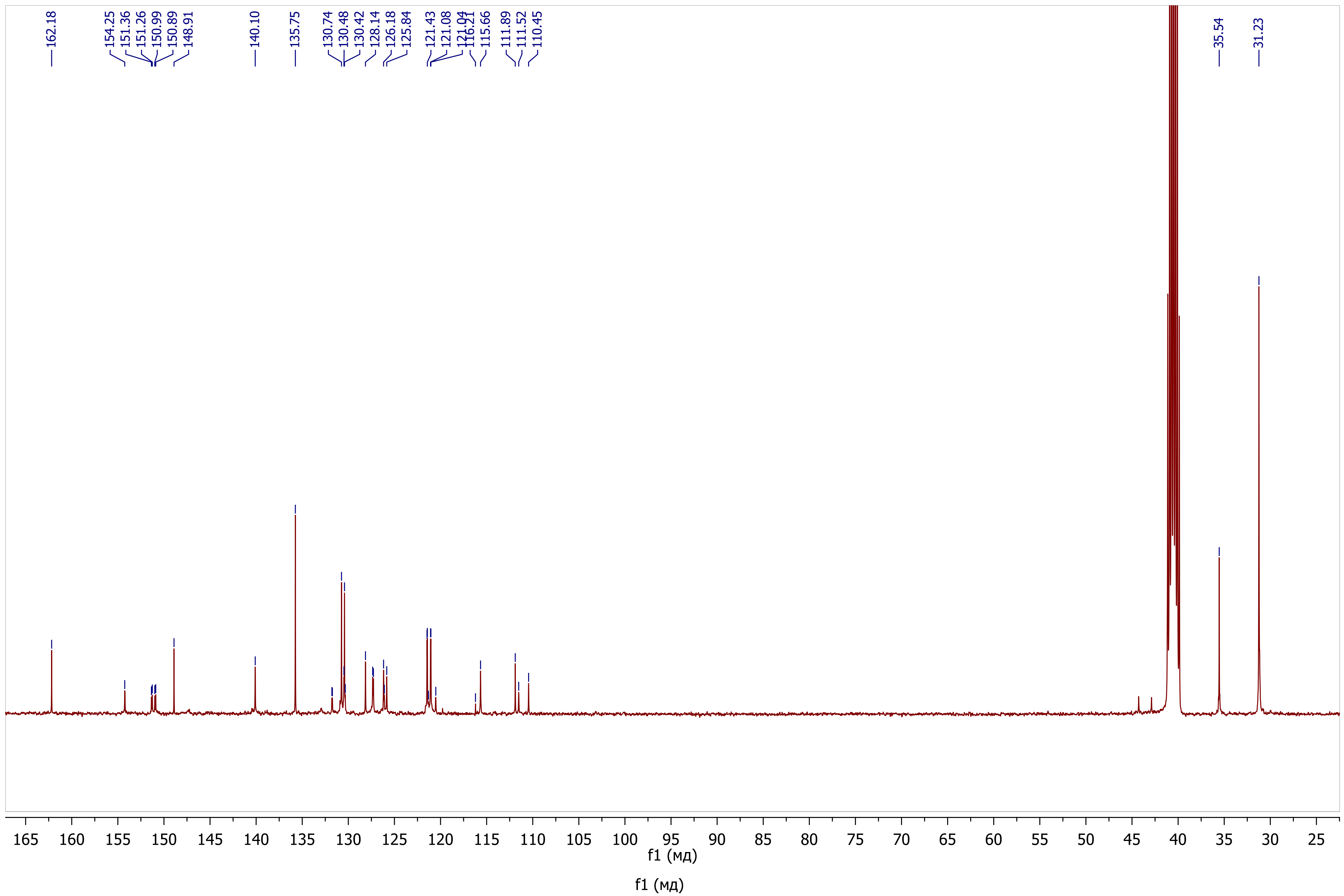

Figure S44. ^{13}C NMR (DMSO-d_6, 126 MHz, 303 K) of compound **5g**

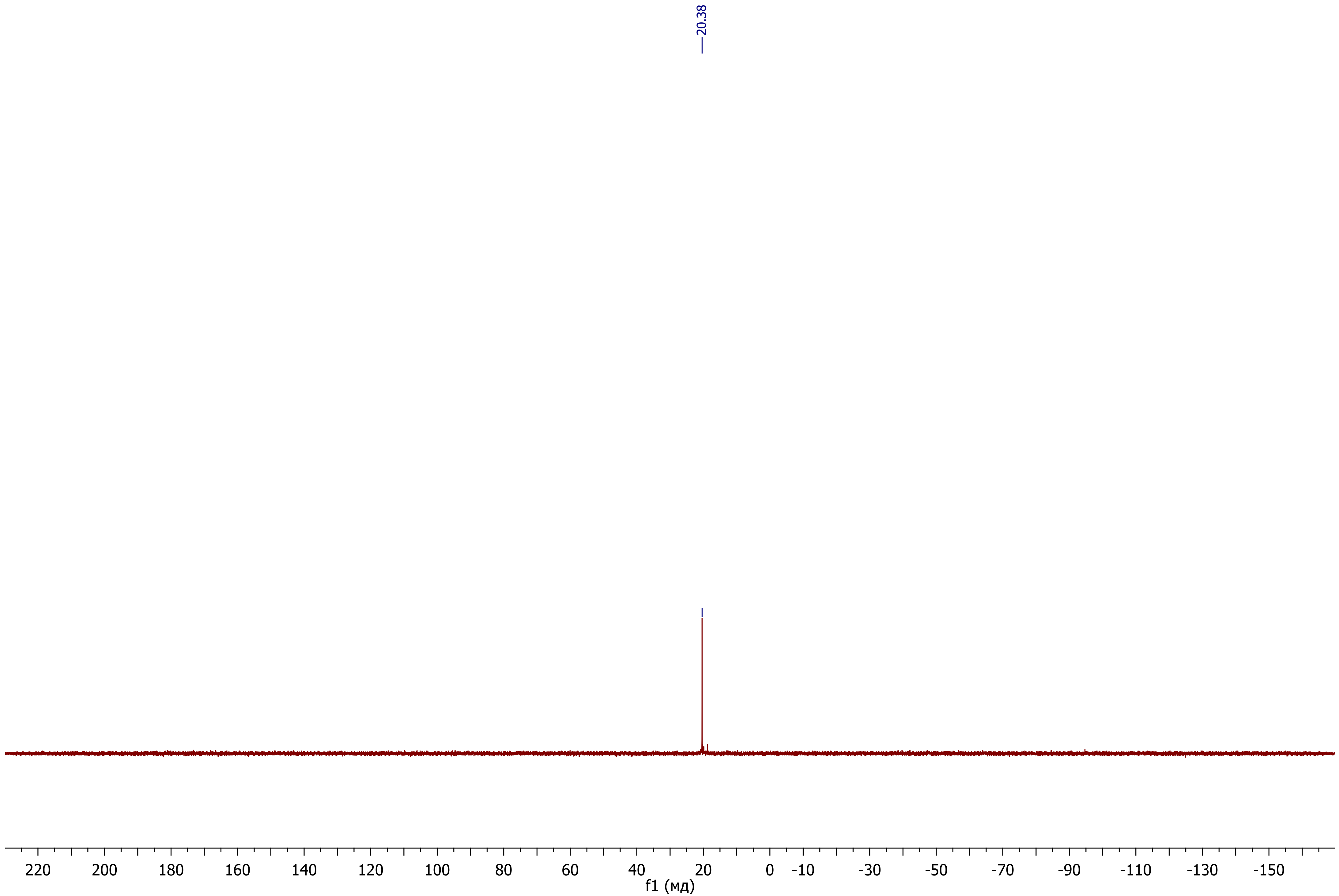

Figure S45. ^{31}P NMR (DMSO-d_6, 162 Hz, 303 K) of compound **5g**

The ability of the test compound to cause destruction of human erythrocytes illustrates its toxic effect on the internal environment of the body. The hemolysis assay is a simple screening test, that can be helpful when studying cytotoxicity in more complex models [1]. In this regard, the hemolytic activity of a new series of compounds in relation to human erythrocytes was investigated. Data on hemolytic and cytotoxic activity are represented by HC_{50} values (the concentration of the test compound that causes 50% hemolysis of erythrocytes in the experiment). It was found that the tested compounds didn't have hemolytic activity (HC_{50} >100 μM), i.e. the synthesized compounds are not capable of destroying human erythrocytes [2,3].

Preclinical studies are carried out on laboratory animals such as mice, rats, etc. Often, it is difficult to withdraw appropriate amount of blood to prepare plasma or serum for the following analysis. Due to this reason, whole blood is the best choice for biological matrix for quantification of a drug candidate and therefore should be used for the development of sample preparation method. Nevertheless, the stability of the agent under investigation in the biological matrix should be taken into account due to the presence of enzymes which can transform xenobiotics even *in vitro* [4], and because of this it may be impossible to develop a bioanalytical method. As such, the stability of test subjects in mouse whole blood was studied. Standard solutions of each substance were prepared in mice whole blood at a concentration of 500 ng/mL. Samples were placed on a shaker and shaken throughout the experiment. Two aliquots (10 μL) were taken from the spikes 5, 15, 30 min, 1, and 2 hours after the start of the experiment. Peaks on the chromatograms corresponding to the substance were integrated, and time dependence of substance peak area on the chromatogram was plotted relative to the initial value obtained for the 5 min point. Figure S46 shows changes in the area of substances **4c** and **5d** peaks relative to the original values.

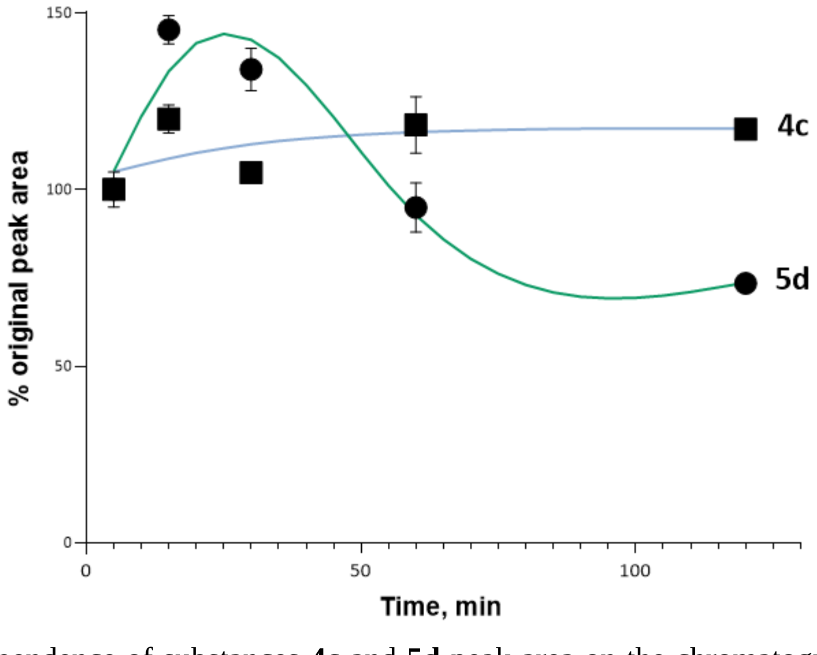

Figure S46. Dependence of substances **4c** and **5d** peak area on the chromatograms of the initial value (5 min).

As can be seen from the figure, for **5d** there is a decrease in the concentration of the substance with time, which indicates a decrease in its concentration in the blood. This indicates that, most probably, this compound is metabolized, but its rate is quite low. Fluctuations in the graph corresponding to the concentration of **5d** in the region of 5–30 min may indicate that in this period of time an equilibrium is established between the substance interacting with blood components (red blood cells and proteins), but subsequently a gradual decrease in its concentration is observed. The concentration of **4c** at the end of the whole experiment was at the same level, which indicates a higher biostability of the substance.

The obtained results are in agreement with general behavior of xenobiotics spiked into a biological matrix. Thus, when a matrix is spiked with some substance, an interaction of the latter with the components of the matrix takes place. In case of whole blood, the compound is adsorbed on the surface of red blood cells as well as its complexes with proteins, primarily albumin and fibrinogen, are formed. The rate of the process depends on the chemical nature of the compound, therefore, the equilibration in the system takes time. As a result, when aliquots of blood are taken and the compound of interest is analyzed, one can observe a significant fluctuation in the obtained results caused with the non-uniformity of the substance concentration in the whole sample. Further, the distribution of the substance between the components of the biological matrix is normalized which can be observed during analysis. Nevertheless, if a compound is not stable in the biological matrix due to some reason (primarily because of its ability to be metabolized with enzymes), further decrease of its concentration will be observed over the time. The decomposition rate will depend on the substance chemical properties, mainly, the presence of chemical groups prone to hydrolysis, e.g. ester ones.

Table S1. *Parameters for detection of compounds by LC-MS/MS in MRM mode.*

Analyte, its parent ion (Q1 m/z, Da)	Fragment ions (Q3 m/z, Da)	DP, V	CE, V	CXP, V
**4c** (714.1)	488,2	-120	-54	-23
	654,2	-5	-34	-27
**5d** (1006.4)	943.2	120	-38	-18
	960.2	120	-40	-19
	989.5	120	-36	-13

References:

1. Tramer, F.; Da Ros, T.; Passamonti, S. Screening of fullerene toxicity by hemolysis assay. *Methods Mol. Biol.* **2012**, *926*, 203–217, doi:10.1007/978-1-62703-002-1_15.

2. Voloshina, A.D.; Gumerova, S.K.; Sapunova, A.S.; Kulik, N. V; Mirgorodskaya, A.B.; Kotenko, A.A.; Prokopyeva, T.M.; Mikhailov, V.A.; Zakharova, L.Y.; Sinyashin, O.G. The structure - Activity correlation in the family of dicationic imidazolium surfactants: Antimicrobial properties and cytotoxic effect. *Biochim. Biophys. acta. Gen. Subj.* **2020**, *1864*, 129728, doi:10.1016/j.bbagen.2020.129728.

3. Voloshina, A.D.; Sapunova, A.S.; Kulik, N. V; Belenok, M.G.; Strobykina, I.Y.; Lyubina, A.P.; Gumerova, S.K.; Kataev, V.E. Antimicrobial and cytotoxic effects of ammonium derivatives of diterpenoids steviol and isosteviol. *Bioorg. Med. Chem.* **2021**, *32*, 115974, doi:https://doi.org/10.1016/j.bmc.2020.115974.

4. Neganova, M.; Aleksandrova, Y.; Suslov, E.; Mozhaitsev, E.; Munkuev, A.; Tsypyshev, D.; Chicheva, M.; Rogachev, A.; Sukocheva, O.; Volcho, K.; и др. Novel Multitarget Hydroxamic Acids with a Natural Origin CAP Group against Alzheimer’s Disease: Synthesis, Docking and Biological Evaluation. *Pharmaceutics* **2021**, *13*, doi:10.3390/pharmaceutics13111893.

Computational SI:

All calculations were conducted using Gaussian16 at the uM06-2X(D3)/6-311++G** level of theory with an ultrafine integral and solvation (SMD=H_2O). The Cartesian coordinates for the optimized structures are provided below.

O_2
E(UM062X) = -150.30956172 a.u.
Imaginary Frequencies = 0
0 3

O	0.00000000	0.00000000	0.59391600
O	0.00000000	0.00000000	-0.59391600

1 2 2.0
2

O_2 Radical Anion
E(UM062X) = -150.45189327 a.u.
Imaginary Frequencies = 0
-1 2

O	0.00000000	0.00000000	0.65881600
O	0.00000000	0.00000000	-0.65881600

1 2 1.0
2

Methylenecyclohexadienone Radical Anion
E(UM062X) = -345.61682357 a.u.
Imaginary Frequencies = 0
-1 2

C	-0.00015600	0.65379000	1.21086700
C	0.00053800	1.41364700	0.00000000
C	-0.00015600	0.65379000	-1.21086700
C	-0.00015600	-0.72119500	-1.21127600
C	0.00001700	-1.48338000	0.00000000
C	-0.00015600	-0.72119500	1.21127600
H	-0.00042300	1.19071500	2.15543000
H	-0.00042300	1.19071500	-2.15543000
H	-0.00026300	-1.26621700	-2.15076400
H	-0.00026300	-1.26621700	2.15076400
C	0.00048400	2.81173200	0.00000000
H	0.00032900	3.36777900	0.92992200
H	0.00032900	3.36777900	-0.92992200
O	-0.00022200	-2.77846000	0.00000000

1 2 1.5 6 2.0 7 1.0
2 3 1.5 11 1.5
3 4 2.0 8 1.0
4 5 1.5 9 1.0
5 6 1.5 14 1.5
6 10 1.0
7

8
9
10
11 12 1.0 13 1.0
12
13
14

Methylenecyclohexadienone

E(UM062X) = -345.49216337 a.u.

Imaginary Frequencies = 0

0 1

C	0.63791500	-1.24809500	0.00000000
C	1.39247100	0.00000000	0.00000000
C	0.63791500	1.24809500	0.00000000
C	-0.70537700	1.25428000	0.00000000
C	-1.46647500	0.00000000	0.00000000
C	-0.70537700	-1.25428000	0.00000000
H	1.20139000	-2.17559200	0.00000000
H	1.20139000	2.17559200	0.00000000
H	-1.27614400	2.17580000	0.00000000
H	-1.27614400	-2.17580000	0.00000000
C	2.73696100	0.00000000	0.00000000
H	3.29549200	-0.92989500	0.00000000
H	3.29549200	0.92989500	0.00000000
O	-2.70120900	0.00000000	0.00000000

1 2 1.0 6 2.0 7 1.0
2 3 1.0 11 2.0
3 4 2.0 8 1.0
4 5 1.0 9 1.0
5 6 1.0 14 2.0
6 10 1.0
7
8
9
10
11 12 1.0 13 1.0
12
13
14

Benzoquinone Radical Anion

E(UM062X) = -381.57245182 a.u.

Imaginary Frequencies = 0

-1 2

C	-0.68193000	1.22684700	0.00000000
C	-1.44141900	0.00000000	0.00000000
C	-0.68193000	-1.22684700	0.00000000
C	0.68193000	-1.22684700	0.00000000
C	1.44141900	0.00000000	0.00000000
C	0.68193000	1.22684700	0.00000000

H	-1.23932100	2.15825100	0.00000000
H	-1.23932100	-2.15825100	0.00000000
H	1.23932100	-2.15825100	0.00000000
H	1.23932100	2.15825100	0.00000000
O	2.71295200	0.00000000	0.00000000
O	-2.71295200	0.00000000	0.00000000

1 2 1.5 6 2.0 7 1.0
2 3 1.5 12 2.0
3 4 2.0 8 1.0
4 5 1.5 9 1.0
5 6 1.5 11 2.0
6 10 1.0
7
8
9
10
11
12

Benzoquinone

E(UM062X) = -381.41377348 a.u.

Imaginary Frequencies = 0

0 1

C	-0.66699400	1.27295900	0.00000000
C	-1.42798700	0.00000000	0.00000000
C	-0.66699400	-1.27295900	0.00000000
C	0.66699400	-1.27295900	0.00000000
C	1.42798700	0.00000000	0.00000000
C	0.66699400	1.27295900	0.00000000
H	-1.25138900	2.18611300	0.00000000
H	-1.25138900	-2.18611300	0.00000000
H	1.25138900	-2.18611300	0.00000000
H	1.25138900	2.18611300	0.00000000
O	2.64626200	0.00000000	0.00000000
O	-2.64626200	0.00000000	0.00000000

1 2 1.0 6 2.0 7 1.0
2 3 1.0 12 2.0
3 4 2.0 8 1.0
4 5 1.0 9 1.0
5 6 1.0 11 2.0
6 10 1.0
7
8
9
10
11
12
